# Are We in Control? How Best to Include a Control Group in Interrupted Time Series Designs: A Simulation Study

**DOI:** 10.1111/jep.70466

**Published:** 2026-05-17

**Authors:** Francesco Manca, Daniel Mackay, Jim Lewsey

**Affiliations:** ^1^ School of Health and Wellbeing University of Glasgow Glasgow Scotland

**Keywords:** autocorrelation, controlled interrupted time series, interrupted time series, parallel trend, simulation studies, spline

## Abstract

**Background:**

While controlled interrupted time series (CITS) are commonly used to evaluate public health policies, how to incorporate control(s) into their statistical modelling has received limited attention. We aimed to compare the statistical performance of different model formulations for including control groups in various segmented regression model specifications (with a particular focus on CITS and Difference‐in‐Difference [DiD] designs) under conditions where their assumptions are met, as well as when they are violated.

**Methods:**

Based on a real‐world dataset, we simulated and compared the statistical performance of four model formulations grounded on segmented regressions for including control groups in a pre‐ and post‐evaluation. The compared model formulations were: (1) CITS segmented regression, (2) DiD segmented regression, (3) single ITS of the difference between control and intervention series, and (4) incorporating the control as a covariate in a single ITS. Models were tested across scenarios challenging assumptions around the control group (e.g., non‐parallel trends ‐challenging DiD assumptions‐, or inconsistent trend difference over time between groups ‐challenging CITS assumption‐) or regression errors (e.g., heteroscedasticity or autocorrelation). We also included models, including restricted cubic splines of time, which may mitigate distortions from assumption violations. Additionally, we tested for detecting non‐parallel trends.

**Results:**

Standard DiD, CITS, and the ITS of the difference between series yielded the lowest bias whenever their design assumptions were satisfied. Overall, including time splines as covariates into ITS of the difference between series achieved the lowest bias and highest coverage also when design assumptions were violated. This makes it a valuable tool for causal inference in settings with parallel, non‐parallel or inconsistent trend patterns between groups. Since violations of the trends assumption are often undetectable, methods robust to such violations are extremely valuable.

**Conclusions:**

Modelling CITS as an ITS of the difference between series is among the most robust methods to embed control series into model specifications. Incorporating time splines as model covariates within an ITS of the difference has the potential of reducing bias from assumption violations (including parallel trends) without negative impacts when assumptions hold.

## 1 Introduction

Interrupted time series (ITS) is a pre‐ and post‐evaluation design becoming increasingly used for evaluation of interventions, especially in clinical research and public health [1]. ITS is closely related to time series, which are sequences of data points representing outcomes at equally spaced time points. While the analysis of time series aims to describe the underlying trend of the series, in ITS designs the focus is on whether the implementation of an intervention happening at a known point in time causes ‘interruptions’ in the underlying trend [2].

ITS are typically analysed using segmented regression (SR) models, which allow estimation of changes in level and slope before and after an intervention. SR applicability is not limited to ITS, but also to other pre‐ and post‐evaluation designs implying a control group, including controlled ITS (CITS) and difference‐in‐difference (DiD), which are among the most used. In this context, a control group is another time series not exposed to the intervention, providing a ‘real‐world’ counterfactual. ITS relies on the pre‐intervention trend for an estimation of the counterfactual, and while they can be judged a robust evaluation method, controlled designs are commonly judged more robust as the real‐world reference of the control can strengthen causal claims [3].

Overall, different designs are based on different assumptions, and such assumptions are reflected on how the SR is parametrised. For instance, DiD main assumption is the parallel trend between groups, meaning that in the absence of the intervention, the difference in outcome level between the trend and control group would have remained the same [4]. In contrast, CITS allows different pre‐intervention trends between groups, assuming that both the change in level and slope of the control group would have been observed in the treated if there was no intervention [4]. (see Supporting Information S1: Appendix A0 for DiD and CITS' SR model formulations).

Therefore, the validity of these evaluations based on time series data is given by a good parametrisation of the underlying process (model specification) as well as on the quality of the control. Indeed, the quality of the control (in terms on how well it is likely to imitate the intervention series) can reduce bias from unmeasured confounders, as it is supposed to incorporate trend changes by co‐occurring post‐intervention events that pre‐intervention trends alone cannot predict [3].

There are many study design considerations in ITS/CITS or DiD. The decision on which statistical model formulation to use should be context‐specific dependent on factors such as: number of data points, type of outcome variable, autocorrelation, seasonality, etc. However, a recent scoping review [1] stressed how ITS has often been modelled using inadequate methods based on such features, leading to potentially biased conclusions about the magnitude and nature of intervention effects [5, 6].

Recent literature offers valuable resources, including tutorials on ITS analysis [2, 7, 8] and comparative analyses of statistical methods [6, 9]. While this growing body of literature is mainly focused on ITS, research on how to compare models for including controls into time series designs remains limited despite their stronger relevance for evidence.

Beyond recommendations on testing assumptions around the control (e.g., parallel trend, heteroscedasticity, autocorrelation, etc.), which are not often performed [1], there are no guidelines on which kind of method to use when there are two or more compared series into a SR framework. Lopez Bernal et al. [3], referring to CITS, distinguished two macro classes of analyses. The first approach is to undertake two single ITS for the control and intervention series and then compare the size of intervention effects in the two series ex post. The second is to include control and intervention series within the same model. They suggested that while the first approach may be easier, the second would account better for unmeasured confounding effects. Despite the general agreement that the second approach is preferable, the literature exhibits considerable diversity in how to implement it. For instance, Lopez Bernal et al. [3] suggested modelling the series as a panel or performing the analysis on a new series of the ratio or of the difference between the intervention and control series at each time point. Most of the papers perform [10, 11] or suggest to perform [3, 7, 12] panel SR or the difference (in outcome) between series. However, alternative methods are common, such as including the control series as a covariate in an ITS design [13, 14, 15, 16], but have received criticisms [7].

Using a large‐scale simulation study, we aim to compare the statistical performance across a range of model formulations for including control groups in SR, as they are the most common modelling framework for estimating intervention effects in time series data. We will compare the performance across different scenarios, challenging ITS assumptions and controls quality.

### 1.1 Motivating Example

The authors were involved in the evaluation of a pilot of a police carriage naloxone intervention intended to reduce overdose deaths in three police areas of Scotland, UK. We followed a CITS study design where the intervention groups were the three police division regions of Scotland implementing the pilot, and the control groups were other Scottish police areas. The outcome variables were fortnightly absolute number of overdose deaths; the intervention covariate was a dummy variable determined by the date of police carriage of naloxone rollout. For each area, we assessed the parallel trend as well as the autocorrelation of the single series and of the difference between series. After evaluating a substantial lack of autocorrelation, we assessed a SR on the level (a DiD, formulated as a panel fixed‐effect (FE)). We performed several alternative statistical models and compared results using root mean squared error. This example informed the parametrisation of the data generating process (DGP) explained in the next sections. To simplify the parametrisation and provide just one intervention and one control group, we used as intervention series the largest police division intervention region (Glasgow) and as a control the most similar area without the policy (Edinburgh). In our simplified model, the overall effect of the policy was a decrease of approximately 2 deaths per unit of time. We also simplified some other assumptions compared to the original motivating example to feed our DGP, see Supporting Information S1: Appendix A2.

## 2 Methods

### 2.1 Simulation Study Design

We undertook a simulation study, comparing the performance of a set of different model formulations to include a control series into a SR. We built the DGP as a CITS with a change in level only. This is one of the most common circumstances that can be considered as an extension of what is a DiD design, which usually involves only one pre and post‐time point. CITS focusing on level change only, or such extended DiD, has two key assumptions: firstly, the allocation of the intervention is not determined by the outcome under study; and secondly, that the difference in level between series before the intervention would have continued unchanged in the absence of the intervention. Each design was then estimated through different regression frameworks. Each model formulation was then fitted across a range of scenarios variating assumptions from the original DGP. The general model specification of DiD and CITS and their references for this study are in Supporting Information S1: Appendix A0.

### 2.2 DGP

Our DGP consisted of a revised version of the model by O'Neill et al. [17]. They designed a simulation study investigating the parallel trend assumption in a standard DiD by including an unobserved component varying over time and between control and intervention groups (equation 1). In our version, we included a common confounder variable (*Post*) in both intervention and control series, activating only in the post‐intervention period, which increased the value of having a control group.

(1) $$Y_{\mathrm{it}}=X_{\mathrm{it}}\beta_{1}+\mathrm{Post}\;\beta_{2}+\tau \left(D\times \mathrm{Post}\right)+\lambda_{t}\mu_{i}+\varepsilon_{\mathrm{it}}$$

Where Y is the outcome variable, *i* represents the group, and *t* denotes the particular time period. *X* is an observed covariate, $\mu_{i}\;$is an unobserved confounder. $\lambda_{t}$ represents the effect of the unobserved covariates ($\mu_{i}$) over time. *D* is a dummy variable being 1 for the treatment group and 0 for the control. *Post* is another dummy variable being 1 in the periods post‐intervention and 0 in those before. Therefore, *τ* represents the ‘effect’ size of the intervention in the DiD design and $\beta_{2}$ the effect of a confounder which equally affects the control and intervention groups. The intervention is assumed to start in the midpoint of the time series. $\varepsilon_{\mathrm{it}}$ is a normally distributed error. The DGP parametrisation was based on the motivating example. The step‐by‐step explanation of the DGP and its parametrisation are in the Supporting Information S1: Appendix A3 and A5. To match the motivating example and reflect [17], only the ‘step change’ *τ* was considered, but not a slope change.

### 2.3 Scenarios

In the baseline scenario,$\;\lambda_{t}$ was equal to 1 for all *t*, and the DGP reflected a DiD with an absence of underlying trend. We then built variations to the DGP in equation 1 creating three alternative scenarios: one introducing diverging underlying trends across groups (ergo, challenging the DiD parallel trend assumption and transforming the equation into a CITS design) and the other two challenging error assumptions (heteroscedasticity and error autocorrelation).

When we challenged the parallel trend assumption, $\lambda_{t}$ varied over time, resulting in a differential pre‐intervention trend and only a post‐intervention level effect.

**Figure 1:**
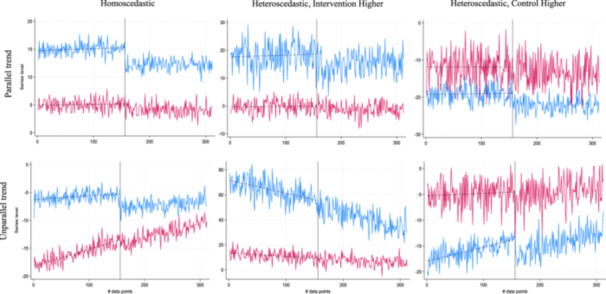
Examples of the six combinations of homoscedastic and parallel trend scenarios with 312 data points and independent errors. Intervention and control series are in light blue and red colours, respectively. Dashed lines represent the pre‐intervention linear trend; the vertical black line is in correspondence with the interruption (at the 156th data point).

The baseline scenario was homoscedastic with intervention group with higher than the control. When we challenged homoscedasticity, the variance of the errors varied over time. To understand whether the relative level of the control or of the intervention group is important to assess a method's performance, we developed two scenarios for heteroscedasticity (one with the intervention group higher than the control and another with the opposite). Errors were normally distributed, with mean 0 and standard deviation (SD) $\sigma_{\varepsilon }$ equal to $\;\sqrt[{2}]{\left|X_{\mathrm{it}}\beta_{1}+\mathrm{Post}\;\beta_{2}+\tau \left(D\times \mathrm{Post}\right)+\lambda_{t}\mu_{i}\right|}$ in the series with higher variance, and variance equal to 0.5*$\sigma_{\varepsilon }$ in the series with lower variance. Although the actual series level was dependent on the random draws, this formula generated a variance in the heteroscedastic scenarios greater than in the homoscedastic, and it increased with the level of the independent variable (see Figure 1 for the six combinations of heteroscedastic and parallel trend scenarios).

The baseline was assuming error independent and identically (normally) distributed (i.i.d.), meaning an absence of autocorrelation (and heteroscedasticity). We built an alternative scenario with $\varepsilon_{\mathrm{it}}=\rho \varepsilon_{t-1}+N(0,\sigma_{\varepsilon }^{2})$, where ρ=0.7. Details of how such scenarios were built in the DGP, and are in Supporting Information S1: Appendix A4.

Scenarios were also combined with each other, forming a total of 12 combinations (3 scenarios on error's variance, 2 for parallel trend, and 2 for error autocorrelation) (Table 1). Each combination was simulated for different total numbers of time points: 24, 32, 40, 48, 56, 72, 88, 120, 184, and 312. For time efficiency reasons, we used a simulation based on 300 datasets rather than the conventional 1000. We checked that 300 was an adequate burn‐in period, producing stable results as done in other simulation studies on time series [6]. The analysis and simulation were run with STATA 18.

**Table 1:** Simulation parameters.

Parameters	Symbol	Parameters value
Intervention effect	τ	2
Time‐dependent unobserved effect (parallel trend parameter)	$\lambda_{t}$	1, dependent on t
Error autocorrelation	ρ	0, 0.7
Error SD*	$\sigma_{\varepsilon }$	0.1, $\sqrt[{2}]{\left|X_{\mathrm{it}}\beta_{1}+\mathrm{Post}\;\beta_{2}+\tau \left(D\times \mathrm{Post}\right)+\lambda_{t}\mu_{i}\right|},$ $0.5*\sqrt[{2}]{\left|X_{\mathrm{it}}\beta_{1}+\mathrm{Post}\;\beta_{2}+\tau \left(D\times \mathrm{Post}\right)+\lambda_{t}\mu_{i}\right|}$
Number of data points		24, 32, 40, 48, 56, 72, 88, 120, 184, 312

*Note:* *Based on SDs we developed three scenarios, first homoscedastic (with error s.d. equal to 0.1), second heteroscedastic with intervention with higher level than control (error s.d. equal to $\sqrt[{2}]{\left|X_{\mathrm{it}}\beta_{1}+\mathrm{Post}\;\beta_{2}+\tau \left(D\times \mathrm{Post}\right)+\lambda_{t}\mu_{i}\right|}$ in the intervention and 0.5*$\sqrt[{2}]{\left|X_{\mathrm{it}}\beta_{1}+\mathrm{Post}\;\beta_{2}+\tau \left(D\times \mathrm{Post}\right)+\lambda_{t}\mu_{i}\right|}$ in the control group); third heteroscedastic with control with higher level than intervention (error s.d. equal to $\sqrt[{2}]{\left|X_{\mathrm{it}}\beta_{1}+\mathrm{Post}\;\beta_{2}+\tau \left(D\times \mathrm{Post}\right)+\lambda_{t}\mu_{i}\right|}$ in the control and 0.5*$\sqrt[{2}]{\left|X_{\mathrm{it}}\beta_{1}+\mathrm{Post}\;\beta_{2}+\tau \left(D\times \mathrm{Post}\right)+\lambda_{t}\mu_{i}\right|}$ in the intervention group).

An additional sensitivity analysis changing the unparallel trend features was run. Here, an additional trend covariate, decreasing and increasing over time at irregular intervals, affected only the control series. This was done to generalise the findings with different kinds of unparallel trends and violating both DiD and CITS standard assumptions. Specifications of this scenario are in Supporting Information S1: Appendix A9.

### 2.4 Performance Measures

The performance of each model formulation was compared using the following performance measures [18] referred to the intervention coefficient (τ in the DGP): bias (in absolute value), the ratio between average model standard error (avgModSE) and the empirical standard error (EmpSE), and the coverage. The bias is the difference between the expected value of a parameter and the actual value of the parameter; the ratio avgModSE/EmpSE measures how much the SEs estimated by the model are similar to the observed SD of the estimate, and it is based on the avgModSE (the average of the standard errors reported across all simulations) and the EmpSE (the SD of the estimator across simulations); the coverage is the probability that the confidence intervals contain the true parameter value. The performance measures are extensively defined in Morris et al. [18].

Also, additional regressions were performed on a dataset of biases to assess how much the bias depended on specific scenario and model characteristics. In these regressions the bias of each statistical technique was the dependent variable, and each scenario's characteristics (e.g., heteroscedasticity or parallel trends) were the independent.

Lastly, we also verified the power of detecting pre‐treatment parallel trends in unparallel scenarios generated by our DGP. We did this by regressing the difference between the control and intervention series in pre‐intervention periods (outcome variable) on time (covariate).

### 2.5 Estimation Methods

While the baseline DGP reflected a DiD with a panel Fixed Effect (FE) model specification with all assumptions met, the scenarios varied these assumptions, extending to unparallel trends and departure from i.i.d. errors. Across all the different scenarios, we focused on comparing different statistical modelling options to include controls as well as using common regression methods within each option. Specifically, the control was inserted in four different ways: (1) a CITS SR—taking into account differences in pre intervention trend—, (2) a DiD SR ‐not considering differences in pre intervention trend—(3) a ITS of the difference between control and intervention—a common alternative way to express a CITS with change in level only—(4) incorporating the control as a covariate in a single ITS (Table 2). In addition, we used an uncontrolled ITS for comparison purposes. All combinations of including the control, were then examined based on different regression methods (i.e., SR was analysed with panel FE, panel FGLS, and others). Each model was then analysed under the 12 scenarios described earlier (Table 2).

**Table 2:** Summary of statistical modelling and estimation methods.

Modelling option to incorporate control	Statistical estimation method
1. CITS segmented regression $Y_{\mathrm{it}}=X_{\mathrm{it}}\beta_{1}+\mathrm{Post}\;\beta_{2}+\mathrm{time}\;\beta_{3}+\tau \left(D\times \mathrm{Post}\right)+{\left(D\times \mathrm{time}\right)\beta_{4}+\alpha_{i}+\varepsilon }_{\mathrm{it}}$	1.1. Panel Fixed Effect: model including both cross sectional and time series components, with unobserved individual time‐invariant effects $\alpha_{i}\;,$ pre‐intervention trend variations and underlying time trend. (FE T)
	1.2. Panel FE, Driscoll‐Kraay: as in 1.1. but with standard errors robust to general forms of cross‐sectional and temporal autocorrelation, it is common when the time dimension is large compared to cross section dimension (DK T)
	1.3. Panel Fixed Effect splnT: as in 1.1. and splines of time as covariates instead of the underlying time trend (splnT)
	1.4. Feasible generalised least squares: as in 1.1. but with an error correction allowing for autocorrelation within panels, cross sectional correlation and heteroscedasticity across panels. (FGLS T)
2. Difference‐in‐difference framework $Y_{\mathrm{it}}=X_{\mathrm{it}}\beta_{1}+\mathrm{Post}\;\beta_{2}+\mathrm{time}\;\beta_{3}+\tau \left(D\times \mathrm{Post}\right){\;+\varepsilon }_{\mathrm{it}}$	2.1. Panel Fixed Effect: model including both cross sectional and time series components, with unobserved individual time‐invariant effects $\alpha_{i}\;$ without underlaying time trend (no $\beta_{3}$). (FE)
	2.2. Panel Fixed Effect: as in 2.1 but with underlying time trend (FE T)
	2.3. Panel FE, Driscoll‐Kraay: as in 2.1. but with standard errors robust to general forms of cross sectional and temporal autocorrelation (see 1.2). (DK T)
	2.4. Panel Fixed Effect: as in 2.1. and splines of time as covariates (splnT)
	2.5. Feasible generalised least squares: as in 2.2. but with an error correction allowing for autocorrelation within panels, cross‐sectional correlation and heteroskedasticity across panels. (FGLS T)
3. ITS of the difference between control and intervention series ${\mathrm{Diff}}_{t}=\mathrm{time}\beta_{1}+\mathrm{diff}X_{t}\beta_{2}+\tau P\mathrm{ost}+\varepsilon_{t}$	3.1 OLS having as independent variable the intervention dummy and not time trend and not difference in covariates. (Diff)
	3.2 OLS: as in 3.1 but including as a covariate the series of the difference between intervention and control's covariates (Diff X)
	3.3 OLS as in 3.1 but including as a covariate the underlying trend (Diff T)
	3.4 OLS as in 3.2 but including as a covariate the underlying trend (Diff X T)
	3.5 OLS as in 3.1 but including splines of time as covariates (Diff splnT)
	3.6 OLS as in 3.2 but including splines of time as covariates (Diff X splnT)
4. ITS of the intervention series and control as a covariate* $Y_{t}={\mathrm{Control}}_{t}\beta_{1}+X_{t}\beta_{2}+\mathrm{time}\beta_{3}+\tau \mathrm{Post}+\varepsilon_{t}$	4.1. OLS having as dependent variable the intervention series and as covariates the intervention covariates and the control series, but not the underlying linear time trend (no $\beta_{3}$). (OLS C)
	4.2 OLS as in 4.1 but including linear underlying time trend (OLS C T)
	4.3 OLS as in 4.1. but including splines of time as covariates (OLS C splnT)
5. Uncontrolled ITS* $Y_{t}=X_{t}\beta_{1}+\mathrm{time}\beta_{2}+\tau \mathrm{Post}+\varepsilon_{t}$	5.1 OLS: single ITS of the intervention group with intervention covariates with no underlying linear time trend (no $\beta_{2}$). (OLS)
	5.2 OLS as in 5.1 but with underlying linear time trend (OLS T)
	5.3 OLS as in 5.1 but with splines of time as covariates (OLS T splnT)

*Note:* *In the error autocorrelated scenario, this models had error autocorrelated correction following the ARMA framework to model the form $\varepsilon_{\mathrm{it}}=\rho \varepsilon_{t-1}+N(0,\sigma_{\varepsilon }^{2})$. In brackets there is the abbreviation used for reference to Supporting Information S1: Appendix Tables and Figures.

While the motivating example did not have an underlying trend, we included covariates allowing for linear or curvilinear trends commonly used in epidemiological research (e.g., linear interaction terms of treatment groups and time or restricted cubic spline [19] of time), see Table 2. The aim was to use such terms to counterbalance the potential distortion coming from some of the violated assumptions. Splines' knot placement was based on Harrell's recommendations [20], as per STATA default option; the number of knots was based on information criteria. For scenarios challenging the assumption of homoscedasticity, any technique not already employing error adjustments adopted the White estimator. The White estimator is supposed to produce heteroskedasticity‐consistent standard errors while leaving the coefficient estimates unchanged.

For the base case, we also undertook two single ITS analyses for the control and intervention series and estimated the intervention effect (and the resulting bias) by subtracting one from the other. As mentioned, this method is less recommended [3]. For this reason, it is reported only for completeness regarding bias in Supporting Information S1: Appendix A10.

## 3 Results

**Figure 2:**
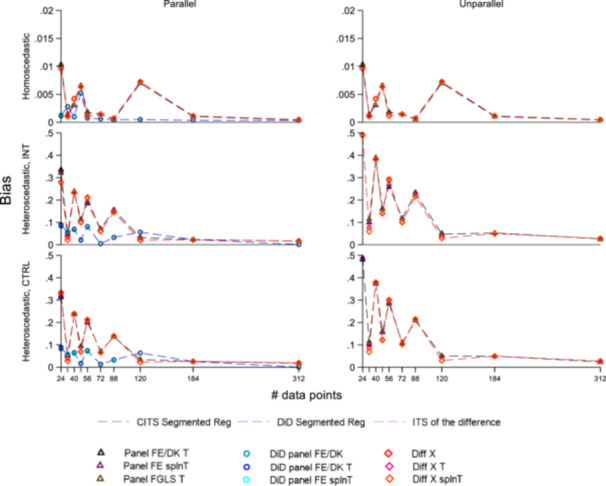
Panel FE and DK have the same bias as the difference in the statistical regression only concerns SEs, so Panel FE without error correction and DK are shown together in Figure 2. Many modelling estimations appear overlapped, for detailed identification of bias see Tables Supporting Information S1: Appendix A1a‐1b. Estimates with bias higher than 0.5 (corresponding to a 25% of the coefficient of interest) were removed from Figure 2 for presentation purposes ‐this includes also control as a covariate and uncontrolled models‐. As per Table 2, abbreviations are: within CITS—segmented regression: DK = Driscoll‐Kraay standard error, Panel FE T = panel fixed effect errors with linear time trend in the model, Panel FE splnT = panel fixed effect and spline of time, Panel FGLS T = panel with feasible generalised least squares and linear time trend in the model. Within the DiD—segmented regression: DK = Driscoll‐Kraay standard error without linear time trend as covariate, panel FE = panel fixed effect errors without linear time trend as covariate. Within the ITS of the difference: Diff X = difference including the series of the difference of the control and intervention's covariates as a covariate; Diff X T: as Diff X but including a linear time trend as a covariate; Diff X splnT: as Diff X and spline of time as covariate. *Note:* the scale for the top panels is different than the scale for the rest of the figure. The relative peak in CITS and ITS of the difference at 120 was likely due to the number of 300 simulations, increasing it produced a smoother bias profile.

**Figure 3:**
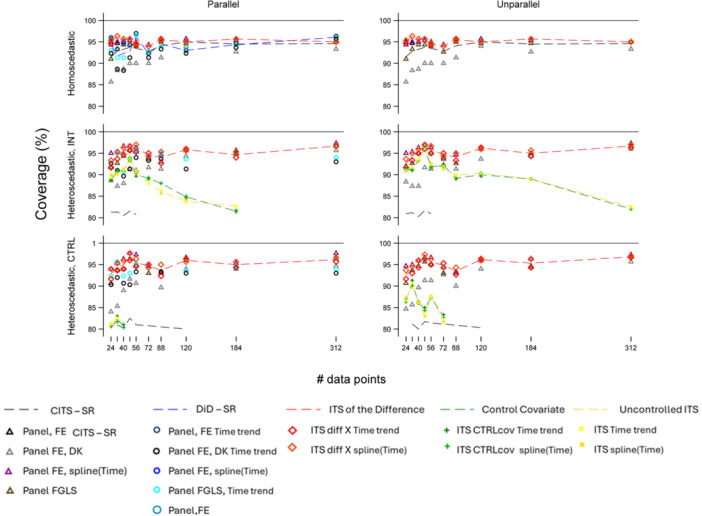
Coverage across different parallel and heteroscedastic scenarios for Error uncorrelated scenario. For abbreviations, see Table 2 and Figure 2.

Performance measures for the independent error scenarios are described in Figures 2, 3. Corresponding figures and tables on scenario analysing autocorrelated errors are in Supporting Information S1: Appendix A1b; description and representation of the ratio avgMSE/EmpSE results are in Supporting Information S1: Appendix A6a‐c. For conciseness, in autocorrelated scenarios single ITS including control as a covariate and uncontrolled ITS were represented exclusively with ARMA components. This choice reflects the well‐recognised fact that incorporating ARMA structures into temporally correlated data provides more accurate standard error estimation compared to specifications without such components.

### 3.1 Bias

#### 3.1.1 Bias and Sample Size

In general, when models correctly reflected the DGP, increasing the number of observations reduced the bias with differences across estimation methods (Supporting Information S1: Appendix A1a‐b). Overall, while in a few cases, misspecified models may outperform specifications reflecting DGP in small samples (e.g., ITS with control as a covariate and uncontrolled ITS), their bias remained constant or increased as the number of observations grew.

#### 3.1.2 Independent Errors

##### 3.1.2.1 Homoscedastic

Under *parallel trends*, all methods reflecting the DGP—including CITS, DiD, and ITS of the difference—demonstrated low bias (maximum of 0.07 across all sample sizes—3.5% of the effect size—). The lowest bias occurred in models such as DiD with no time trend or a linear trend only; adding extra time terms had a negligible impact in large samples and is only minorly noticeable in smaller ones, shifting bias by approximately 0.0001 (0.055% vs. 0.06% of the effect size). With *unparallel trends*, the DGP had a linear divergence between series, and including a time component was crucial to achieve a similar low bias to the parallel scenario. Standard DiD estimation models had higher biasCITS and ITS of the difference, which remained the most reliable designs, provided they embed time components and differenced covariates. There was no consistently significant preference between linear trends and splines across different sample sizes.

Regarding ITS of the difference, in both scenarios, including differenced covariates led models to consistently outperform those without them. In contrast, designs misspecified—such as using the control as a covariate or uncontrolled series—maintained high bias, ranging from 0.994 (49.7%) to 1.003 (50.1%) in parallel trends. This bias increased in non‐parallel scenarios.

##### 3.1.2.2 Heteroscedasticity

Overall, heteroscedasticity increased the bias across all models. In *parallel* settings, the best performing models were standard DiD (with or without time covariates) and ITS of the difference (without time trends)—with bias reaching 0.01% and 0.16% of the effect, respectively with *n* = 312—. The second‐best models were CITS and DiD with time trends (including splines); while these models had relevant bias in small samples (all between 14% and 16% at *n* = 24), they showed a decreasing bias in larger samples (between 0.97% and 0.86% at *n* = 312). In *unparallel* scenarios, the preferred designs were CITS and ITS of the difference, provided they included time components.

Which group (control vs. intervention) had higher variance was not a major diriver of the bias and its relevance changed across methods. In paralell scenario, standard panel DiD frameworks had lower bias when the controlled series had higher variance; similarly in unparallel scenario, CITS had lower bias when control had higher bias.

#### 3.1.3 Autocorrelated Errors

Overall, autocorrelated errors did not affect the bias; the ranking of the preferred models largely reflected the independent errors scenario.

OLS regressions using bias as the dependent variable (Supporting Information S1: Appendix A4) confirmed most of these results. Specifically, the CITS and ITS of the difference (including time components) proved to be the most resilient designs, with no scenario significantly impacting their bias. In contrast, the regressions analysing DiD models were sensitive to both high variance in the control series and unparallel scenarios. Similarly, while models using a control as a covariate and uncontrolled models were sensitive to these same scenarios, they also exhibited higher baseline biases, as indicated by higher regression intercepts.

### 3.2 Coverage

#### 3.2.1 Independent Errors

##### 3.2.1.1 Homoscedastic

Under *parallel trends*, all models aligned with the DGP had the highest coverage. Specifically, DiD and ITS of the difference specifications ‐particularly in the absence of splines‐ exceeded 96% at *n* = 312. Although specifications CITS and ITS of the difference incorporating time components (linear or splines), maintained coverage above 94% across all sample sizes, they were not the optimal choices in this scenario. Conversely, in non‐parallel contexts, CITS and ITS of the difference models featuring time components (linear or splines) were the preferred specifications. For smaller sample sizes (*n* ≤ 48), the ITS of the difference was preferred, whereas no distinct model preference emerged in larger samples.

##### 3.2.1.2 Heteroscedastic

In parallel scenarios, CITS models with splines and all specifications of the ITS of the difference—regardless of the choice of time covariate—were the preferred models, maintaining an average coverage exceeding 94%. In non‐parallel scenarios, CITS and ITS of the difference models with splines outperformed all alternative specifications, with coverage exceeding 93% at *n* = 24% and 97% at *n* = 324.

Overall, whether the higher variance was in the intervention or the control series had no significant impact on the results, with the exception of single ITS models. These models exhibited higher coverage when the intervention series had higher variance; while their performance improved with the inclusion of time covariates, their coverage remained consistently lower than that of other model specifications.

#### 3.2.2 Autocorrelated Errors

Generally, coverage was lower with autocorrelated errors. In *parallel* scenarios, DiD models with Driscoll‐Kraay (DK) standard errors were the most robust alternative with highest coverage. In contrast, in *unparallel* scenarios, the use of DK errors within CITS designs was the best alternative. While in these scenarios the ITS of the difference was not the best specification, it kept coverage consistently exceeding 81% in small samples and reaching 87% in larger samples—provided that linear or spline time covariates were included—. Finally, single ITS models incorporating ARMA components exhibited high coverage (exceeding 90% at *n* = 312) this was limited to unparallel trends and only when the intervention series had higher variance.

### 3.3 Detection of Unparallel Trend

With homoscedasticity and unparallel settings, 80% and 95% of simulations rejected the null hypothesis of parallel trend in small (24) and bigger samples (312), respectively (Supporting Information S1: Appendix A8). In contrast, percentages were substantially lower in unparallel and heteroscedastic settings (which implied a higher overall variance in our DGP), being 24%−25% and 65%−68% in small and big samples, respectively.

### 3.4 Alternative Unparallel Scenario

When groups have irregular non‐linear unparallel trends, the ITS of the difference with a spline of time was a superior model specification (Supporting Information S1: Appendix A9b). This approach demonstrated a bias that decreased with sample size. While the bias was relevant in low samples, it reached 0.0117 with *n* = 312 (0.6% of the true effect). In contrast, CITS models generally showed bias exceeding 1.0, surpassing the true effect by more than 50%. While misspecified models—such as uncontrolled ITS or models using the control as a covariate—occasionally yielded low bias in small samples, their bias remained constant or grew in larger samples.

Regarding coverage, the ITS of the difference, including spline (without adjusted covariates) was the only specification that produced both precise variance estimates and low bias producing always a coverage ≥ 82%; when time covariates were added there was an overestimation of the variance producing wider confidence intervals and then coverage reaching 95%. CITS models occasionally had higher bias (61% with *n* = 24% and 79% with *n* = 312 when splines were added) however they could achieve high coverage. This was primarily driven by an overestimation of the variance, which artificially inflated the confidence intervals.

## 4 Discussion

SRs are the most common statistical models estimating CITS and DiD designs for causal inference when evaluating interventions using real‐world data. However, the assumptions underlying these designs are often untested, despite being likely violated in real‐world settings—an oversight with potential policy implications. We investigated the performance of traditional regression methods for incorporating controls, both with and without tools to alleviate the effect of potential confounders (splines), when key assumptions (parallel trends, homoscedasticity and error autocorrelation) are violated.

Overall, we found that whenever designs (CITS, DiD and ITS of the series' differences) and estimation methods (e.g., different regression estimators) reflected the baseline DGP, accommodating assumptions on trends and deviations from errors i.i.d, they performed well in terms of low bias, high coverage and accurate variance. In addition, while some designs or estimation methods were not the first best in terms of performance as they did not strictly match DGP regarding designs or error's form, they may still have good performance, which in certain circumstances can also exceed that of correctly matching specifications. This is the case of the inclusion of time components (linear or splines) in parallel scenarios (which did not exhibit a time trend), where DiD using time components and ITS of the differences using splines had comparable bias and often wider coverage compared to DiD models without time components (the exact DGP specification). This happened more often in high variance contexts. In such cases —despite a slight increase in bias (e.g., reaching 0.017%–0.85% of the effect size in parallel scenarios with a higher intervention variance and *n* = 312)— the coverage was higher.

The advantage of incorporating time components became evident when dealing with non‐parallel trends, and especially in high variance contexts. While standard DiD models had poor performance even when incorporating time components, designs such as CITS and ITS of the difference explicitly matching these contexts provided the lowest biases and highest coverages. The way time was parametrised (linear or non‐linear through splines) was relevant in unparallel contexts facing high variance where, on average, splines in CITS and ITS of the difference were superior across most of the sample sizes (especially in high dimension samples). Specifications including splines of time were also preferred in the alternative scenario challenging both CIST and DiD trend assumptions.

Regarding designs, ITS of the difference is a simpler approach compared to panel‐based specifications. In our scenarios with higher variance, on average, it showed more robust performance than CITS (under both parallel and non‐parallel trends) and DiD (under parallel trends); its performance was comparable only to CITS using splines. This likely reflects the fact that reducing the analysis to a single differenced series can simplify the error structure. Specifically, gains from using a single series of the difference may arise when the treated and control series are highly positively correlated. In this case the overall variance of the difference would be lower than the sum of the variances (this is plausible as control series are usually selected to match as close as possible the pattern of the intervention series). The lower variance can reduce the finite samples bias through gains in precision. In high variance contexts and big samples, splines in CITS design may do the same job by allowing for more flexible baseline trend and alleviating the impact of noise on estimates. In smaller samples, beyond the variance itself, the ITS of the difference could also lead to gains in variance estimation as it avoids the need to model both within—and between—series dependence.

The combination of a flexible design with flexible time covariates (splines) was the real advantage in contexts where both CITS and DiD assumptions were violated. Indeed, in the unparallel trend scenario—where the control series exhibited a trend that changed over time—the ITS of the difference using splines was the only method achieving low bias in large samples (*n* > 56) and high coverage across all samples. These findings have significant policy implications, as violations of the parallel trends assumption—especially in heteroscedastic/high variance or small sample settings—are frequently undetected (Supporting Information S1: Appendix A8). Furthermore, CITS's assumptions on constant differences in slope may be even harder to detect. We argue that prioritising the prevention of the effect of these violations is a valuable trade‐off in contexts where there is no high confidence in assumptions holding. Although this may lead to a marginal loss in efficiency or precision under ideal conditions, it may serve as an insurance strategy, reducing the bias from misspecification and providing information on the treatment effect (while maintaining a cautious stance on its exact magnitude, especially in small samples).

### 4.1 CITS and ITS of the Difference, Model Comparison

Overall, the ITS of the difference is often preferable to CITS, as the latter relies on assumptions that are both fragile and difficult to verify. Specifically, in scenarios involving irregular slope trends between series, CITS models, which include additional coefficients, are inherently less flexible and can produce bias even when splines are included. Specifically, while DiD assumptions are partially testable, CITS assumptions allowing for unparallel trends but only through constantly diverging slopes, are difficult to test (and potentially rare in the real world). The ITS of the difference between the two series, analysing only a change in level, provides a unified reparameterization of these two designs in a univariate time‐series setting, with simpler specification. Adding a spline of time can further increase flexibility in settings where assumptions on the linearity or parallel trends are challenged. This provides a significant advantage over CITS, as it avoids modelling the two series separately within a panel framework.

Recent evidence [21] suggests that parallel trend assumptions are often highly implausible in the literature. Furthermore, when testing how robust findings are to potential violations of parallel trends, treatment effect estimates can be statistically indistinguishable from realistic trend violations or sampling error. In this context, ITS of the difference with splines of time should be preferred, especially in large samples and high variance frameworks, when there is no strong confidence in linear parallel trends.

Despite the flexibility of the ITS of the difference with splines, it is worth noting that this strategy can face limitations, specifically regarding serial correlation and sample size. In our scenario of severe autocorrelation (ρ = 0.7), the ITS of the difference was not the best strategy for coverage, as it had an underestimation of the variance (ratio < 1, Supporting Information S1: Appendix A6). This was likely due to a complex error structure resulting from differencing two series with autocorrelated errors. In such cases, CITS or DiD specifications utilising DK error corrections provided better estimation of the variance (and coverage), especially in large samples. Additionally, while splines are more efficient in large samples, they are sensitive to noise in small‐sample contexts. Consequently, the trade‐off between spline complexity (kind of spline and number of knots) and linear trend should be guided by established strategies, such as using information criteria (e.g., AIC or BIC) to avoid overfitting.

### 4.2 Alternative Methods

While inserting the control as a covariate is seemingly straightforward and sometimes applied, it was not an effective design strategy for minimising bias, even under ideal Gauss‐Markov conditions. Adding a time spline sometimes provided higher coverage but was not consistently the optimal choice. While such a model specification could improve estimation of the variance (especially under an error autocorrelated scenario, by using ARMA components), it cannot correct bias coming from a wrong functional form. Specifically, this design fails to facilitate a proper comparison between groups, focusing instead on the treatment series. The increased bias observed when the control series has higher variance confirms that these models are prone to error by incorporating noise directly into the independent covariates, as previously suggested [7].

### 4.3 Implications for Practice

As in all simulation studies, our findings are applicable to our ‘parameter space’ and may not apply to other observational studies with different characteristics proved to affect overall model performance, such as non‐stationary or different autocorrelation structures. However, our parameter values were based on a real‐world data set, and we applied several variations to our base case scenario. Therefore, we believe that a few insights and suggestions can be provided for cases similar to our modelled scenarios:

- ITS using differences between series and including time splines generally yields low bias and high coverage, making it a valuable tool for causal inference in models with either parallel or non‐parallel trends. When all assumptions are violated, it is the only model with the potential of allowing high coverage and low bias in big samples, thanks to its simplicity and high flexibility. This strategy could be used as a complementary analysis when it is not already the base case.
- Violations of parallel trends are often statistically undetectable: the overall parallel trend assumption is only partially testable [7] (the hypothesis requiring that trends would be parallel in the post‐intervention period if there were no interventions is untestable). In high variance or low sample scenarios, an unparallel trend is not often detectable due to power. Therefore, acting preventively using methods with the potential of being robust to unparallel settings can be a safe strategy to triangulate results, even when tests and theory suggest parallel trends or constant difference between slopes.
- Although severe errors' correlation does not affect the bias, it can impact the estimation of the variance (with repercussions on the coverage). While in this context the ITS of the difference does not inherently keep or increase the original error correlation, it may have repercussions on other error features, necessitating a rigorous analysis of the resulting error patterns. Such analysis could indicate the application of estimators robust to both heteroscedasticity and autocorrelation (e.g., Newey‐West).
- When there is high confidence of parallel settings, and there is a presence of both heteroscedasticity and autocorrelated errors, controlled SR with DK or FGLS error correction provides the most robust and efficient estimates. In unparallel settings, difference with splines is the most robust option. However, it may benefit from estimators robust to heteroscedasticity and autocorrelation, and the comparison with other methods remains practical.

### 4.4 Limitations

While we considered several variations to the original DGP [17], the range of scenarios could still be expanded. For instance, a DGP generated on count variables may reflect public health situations. Similarly, generating a non‐linear DGP and fitting both linear and non‐linear models, as in [9], could have offered insights relevant to similar real‐world settings. However, we chose to focus on how additional components commonly used in epidemiology, such as splines, can be incorporated into linear models to address assumption violations. In addition, while the choice of the number and placement of knots is crucial, especially in unparallel patterns, we used restricted cubic splines with Harrell's recommended percentiles technique to choose the knot location. However, a different choice of locating the knots driven by the theory's expectations, such as placing the knot at the intervention point, may produce different results. Indeed, while this choice may capture the structural break of the intervention, it could also produce a sort of ‘confirmation bias' by forcing a significant change at the intervention time and then ‘confirming’ a hypothesis of an intervention effect.

An additional limitation of this study is that we explored only frameworks with one intervention and one control group series. While panel SR can allow for multiple groups, the conclusions can be different based on the correlation within and between intervention and controlled groups. We decided to provide a study with only one control and one intervention to reflect most of the studies. Finally, following [6], we adopted 300 simulations. Even if we tested this assumption and they were sufficient for general trend identification, in specific circumstances, the simulation variability may generate peaks in bias (e.g., see relative peak in some methods at specific sample sizes Figure 2), and a higher number of simulations may be needed to smooth the overall bias profile. Moreover, the Monte Carlo Standard Error of the bias (Supporting Information S1: Appendix A12) indicated that the simulation provided a more stable estimate of the bias at larger sample sizes, even with a constant number of iterations, 300.

We generated a DGP with a level change only. In many cases, level change only specifications are preferred over models including both level and slope changes, as they can focus just on counterfactual differences and on a single coefficient rather than two. However, in contexts where the underlying process also includes a change in slopes, utilising models identifying only a change in level is not incorrect. However, level‐only specifications estimate the average treatment effect over the observation period for the intervention group, rather than the effect at the end of the observation period.

Overall, we excluded further complexity to frame simple, easy‐to‐implement, and readily understandable adjustments and scenarios. This is because ITS designs are frequently employed by researchers without specialised statistical training due to their relative ease of understanding and implementation, and splines are relatively common in epidemiology. Furthermore, including more scenarios or complex non‐linear approaches like Generalised Additive Models would not only have increased computational demands but also added complexity to both the DGP design and understanding.

## 5 Conclusion

The CITS and ITS of the difference between intervention and control series are the most robust methods for embedding a control group into a segmented regression when there are only two available series. Regarding ITS of the difference, the integration of additional components to such standard segmented regression, such as covariates modelling curvilinear time‐outcome relationships using splines, may reduce bias from assumption violations which are often unobserved (e.g., variation of parallel trends over time) without negative impacts when such assumptions are met.

## Author Contributions

Conceptualisation: Jim Lewsey, Daniel Mackay, and Francesco Manca. Data curation: Francesco Manca. Formal analysis: Francesco Manca. Funding acquisition: Jim Lewsey (lead), Daniel Mackay. Methodology: Francesco Manca, Jim Lewsey. Supervision: Jim Lewsey (lead), Daniel Mackay (support). Visualisation: Francesco Manca. Validation: Daniel Mackay. Writing (original draft): Francesco Manca. Writing (review/editing): Francesco Manca and Jim Lewsey.

## Conflicts of Interest

The authors declare no conflicts of interest.

## Supporting information

Supporting File

## Data Availability

Methodological details of the simulation study are found in the main text and supporting materials. STATA code for the base case of the simulation study is provided in Supporting Information S1: Appendix A11; variations to the base case can be derived by changing parameters or models as described in the main text and Supporting Information Material. The data that support the findings of this study are available in the supporting material of this article.
