# Supplementary material for: Are We in Control? How Best to Include a Control Group in Interrupted Time Series Designs: A Simulation Study
Source: J Eval Clin Pract. 2026 May 17;32:e70466. doi: 10.1111/jep.70466 (PMC13180462; doi:10.1111/jep.70466)
Supplement: Supplementary file 1 — Supporting File [file JEP-32-0-s001.docx]

**Appendix**

[A0. CITS and DiD model specification, and relationship with the DGP 2](#_Toc226637361)

[A1a. Bias Models with independent errors 4](#_Toc226637362)

[A1b. Bias Models with autocorrelated errors 5](#_Toc226637363)

[A2. Bias- error autocorrelated scenario.* 6](#_Toc226637364)

[A3. DGP correspondence with motivating example 7](#_Toc226637365)

[A4. OLS regression on bias 8](#_Toc226637366)

[A5. Data generation process. 16](#_Toc226637367)

[A6a. Ratio average model S.E. and empirical S.E. 18](#_Toc226637368)

[A6b. Figure of ratio avgModelSE/EmpiricalSE – independent errors 19](#_Toc226637370)

[A6c. Figure of ratio avgModelSE/EmpiricalSE – errors autocorrelated * 20](#_Toc226637371)

[A7a. Coverage Independent error scenario 21](#_Toc226637372)

[A7b. Coverage autocorrelated error scenario * 22](#_Toc226637373)

[A7c. Figure of Coverage for error autocorrelated scenario* 23](#_Toc226637374)

[A8. Unparallel trend detection. 23](#_Toc226637375)

[A9a. DGP with alternative unparallel trend 24](#_Toc226637376)

[A9b. Results on different unparallel trend (independent error scenario) – sensitivity analysis 24](#_Toc226637377)

[A10. Estimation of the effect (and bias) from the separate analysis of the intervention and control series. 25](#_Toc226637378)

[A11. STATA code for base case scenario and 24 observations: parallel trend, no error autocorrelation, homoscedastic 26](#_Toc226637379)

[A12. Montecarlo SE of Bias - independent errors scenario. 32](#_Toc226637380)

## A0. CITS and DiD model specification, and relationship with the DGP

**Standard Controlled Interrupted Time Series**

Segmented regression is the common estimation method used for CITS. The main assumption of CITS is that, in the absence of the treatment, the treatment group would have followed the same change in level and slope of the control group. It allows for different pre-intervention trends and the intervention effect is the difference in slope plus the difference in level between the intervention groups. The standard CITS segmented regression model is:

𝑌_i𝑡_ = $X_{\mathrm{it}}$𝛽_0_ + 𝑇𝑖𝑚𝑒 𝛽_1_ + 𝑃𝑜𝑠𝑡 𝛽_2_ + D 𝛽_3_ + 𝛽_4_(𝑃𝑜𝑠𝑡 $\times$ 𝑇𝑖𝑚𝑒) + 𝛽_5_ (𝑃𝑜𝑠𝑡 $\times$ D) + 𝛽_6_ (D $\times$ 𝑇𝑖𝑚𝑒) +

+ 𝛽_7_ (𝑃𝑜𝑠𝑡$\times$D$\times$𝑇𝑖𝑚𝑒)+ $\varepsilon_{it}$

Where: *i* represents group, *t* denotes the particular time period. D is a dichotomous variable representing treatment/intervention groups (0=control, 1=treated), Post is a dichotomous variable for periods (0=pre-intervention, 1=post-intervention). $X$ is the set of all other observed covariates over time (which are supposed to adjust for observable confounders). Regarding coefficients interpretation: 𝛽_1_ is the underlying time slope from post-intervention period; *β₂* is the level change in correspondence to the intervention time; *β_3_* is the baseline level difference (treated vs control); *β_4_* is the average change in slope after the intervention across groups; *β_5_* is the difference between intervention and treated groups in change in level after the intervention; *β_6_* is the pre-intervention slope difference (treated vs control); *β_7_* is the difference between the intervention and treated group in change in slope after the intervention. Effect size of the intervention is then *β_5 +_ β_7_.*

**Controlled Interrupted Time Series with level-change only**

Commonly, only the average difference in the post-intervention period is wanted (which it signifies the average post-intervention change in levels). The advantage of this is enclosing an average treatment effect in only one coefficient. In that way ,the CITS with level change only is:

𝑌_i𝑡_ = $X_{\mathrm{it}}$𝛽_0_ + 𝑇𝑖𝑚𝑒 𝛽_1_ + 𝑃𝑜𝑠𝑡 𝛽_2_ + D 𝛽_3_ + 𝛽_5_ (𝑃𝑜𝑠𝑡 $\times$ D) + 𝛽_6_ (D $\times$ 𝑇𝑖𝑚𝑒) +$\varepsilon_{it}$

Effect size of the intervention is then *β_5_.*

**Standard Difference-in-Difference**

The main assumption of a DiD is that, in the pre-intervention periods, the control and intervention groups have a parallel trend; and in the absence of the treatment, the treatment group would have followed the same change in level of the control group (there is no modelling of the trend, which is supposed to be kept parallel and then unchanged).

𝑌_i𝑡_ = $X_{\mathrm{it}}$𝛽_0_ + 𝑃𝑜𝑠𝑡 𝛽_2_ + D 𝛽_3_ + 𝛽_5_ (𝑃𝑜𝑠𝑡 $\times$ D) +$\varepsilon_{it}$

**Standard Difference-in-Difference - DGP notation**

Panel two-way fixed effect segmented regression is the common estimation method for DiD, which giving the assumptions around the error in the panel regression, it can be rewritten as:

𝑌_i𝑡_ = $X_{\mathrm{it}}$𝛽_0_ + 𝑃𝑜𝑠𝑡 𝛽_2_ + 𝛽_5_ (𝑃𝑜𝑠𝑡 $\times$ D) +$\mu_{i}+\varepsilon_{it}$

This is the baseline of our DGP, where $\mu_{i}$is the individual fixed effect, interpretable as the baseline level across groups.

**Deviation from standard Difference-in-Difference – DGP’s Scenarios**

𝑌_i𝑡_ = $X_{\mathrm{it}}$𝛽_0_ + 𝑃𝑜𝑠𝑡 𝛽_2_ + 𝛽_5_ (𝑃𝑜𝑠𝑡 $\times$ D) +${\lambda_{t}\mu}_{i}+\varepsilon_{it}$

By adding $\lambda_{t}$which is a coefficient with a component changing with time, and making it interacting with the individual fixed effect $\mu_{i}$, the model is allowing for pre-intervention different trend (overcoming DiD parallel assumption). In other words, it is like expanding the DiD design into a CITS scenario focused on post-intervention level change-only. Which is the scenario analysis of our DGP allowing for unparallel trend.

## A1a. Bias Models with independent errors

| Heteroscedastic scenario | Parallel scenario | # data points | **Controlled segmented regression (CITS)** | | | | **DiD framework segmented regression** | | | | | **ITS of the difference** | | | | | | | **control as covariate** | | | | **Uncontrolled ITS** | | | |
| --- | --- | --- | --- | --- | --- | --- | --- | --- | --- | --- | --- | --- | --- | --- | --- | --- | --- | --- | --- | --- | --- | --- | --- | --- | --- | --- |
|  |  |  | panel FE/DK* T | Panel FE splnT | panel FGLS T | panel FE/DK* | | panel FE/DK* T | Panel FE splnT | panel FGLS T | diff | | diff X | diff T | diff X T | diff splnT | diff X splnT | Ols C | | Ols C T | ols C splnT | Ols | | Ols T | ols splnT |  |
| Homoscedastic | Parallel trend | 24 | 0.0102 | 0.0103 | 0.0104 | **0.0011** | | 0.0012 | 0.0012 | 0.0013 | 0.023 | | 0.0012 | 0.0699 | 0.0096 | 0.0699 | 0.0096 | 0.9997 | | 0.9947 | 0.9948 | 0.9992 | | 0.9954 | 0.9952 |  |
|  |  | 32 | 0.0015 | 0.0013 | 0.0012 | 0.0028 | | 0.0028 | 0.0028 | 0.0028 | 0.0089 | | 0.0029 | 0.0108 | 0.0013 | 0.0108 | **0.001** | 1.0017 | | 1.0033 | 1.0036 | 1.0017 | | 1.0032 | 1.0031 |  |
|  |  | 40 | 0.003 | 0.003 | 0.003 | **0.001** | | **0.001** | **0.001** | 0.0011 | 0.0264 | | 0.0013 | 0.053 | 0.0043 | 0.053 | 0.0042 | 1.0025 | | 1.0064 | 1.0061 | 1.0023 | | 1.0062 | 1.0062 |  |
|  |  | 48 | 0.0066 | 0.0066 | 0.0066 | **0.0052** | | **0.0052** | **0.0052** | 0.0053 | 0.0244 | | 0.0056 | 0.0294 | 0.0064 | 0.0294 | 0.0063 | 0.9957 | | 0.9975 | 0.9972 | 0.9952 | | 0.9972 | 0.9972 |  |
|  |  | 56 | 0.0018 | 0.0018 | 0.0019 | 0.0007 | | 0.0008 | 0.0008 | 0.0008 | 0.0126 | | **0.0006** | 0.0353 | 0.0011 | 0.0353 | 0.0012 | 0.9977 | | 0.9971 | 0.9971 | 0.9991 | | 0.9985 | 0.9984 |  |
|  |  | 72 | 0.0015 | 0.0015 | 0.0015 | **0.0006** | | **0.0006** | **0.0006** | 0.0006 | 0.0048 | | 0.0008 | 0.0201 | 0.0015 | 0.0201 | 0.0014 | 0.9989 | | 1.0024 | 1.0026 | 0.9991 | | 1.0022 | 1.0022 |  |
|  |  | 88 | 0.0005 | 0.0005 | 0.0005 | 0.0005 | | 0.0005 | 0.0005 | **0.0005** | 0.0145 | | 0.0006 | 0.0413 | 0.0007 | 0.0413 | 0.0007 | 1.0005 | | 0.999 | 0.999 | 1.0004 | | 0.9987 | 0.9987 |  |
|  |  | 120 | 0.007 | 0.007 | 0.007 | 0.0005 | | 0.0005 | 0.0005 | 0.0005 | 0.0145 | | **0.0004** | 0.0492 | 0.0072 | 0.0492 | 0.0073 | 1.0002 | | 1.0037 | 1.0038 | 1 | | 1.0031 | 1.0031 |  |
|  |  | 184 | 0.001 | 0.001 | 0.0011 | **0.0003** | | 0.0004 | **0.0003** | 0.0003 | 0.0052 | | **0.0003** | 0.0227 | 0.0011 | 0.0227 | 0.0011 | 1.0009 | | 1.0004 | 1.0003 | 1.0008 | | 1.0003 | 1.0003 |  |
|  |  | 312 | 0.0004 | 0.0004 | 0.0004 | **0.0003** | | **0.0003** | **0.0003** | 0.0003 | 0.0154 | | **0.0003** | 0.051 | 0.0005 | 0.051 | 0.0005 | 0.9994 | | 0.999 | 0.999 | 0.9993 | | 0.9989 | 0.9989 |  |
|  | Unparallel trend | 24 | 0.0102 | 0.0103 | 0.0104 | 6.5647 | | 6.5774 | 6.5774 | 6.5768 | 6.5526 | | 6.5813 | 0.0699 | **0.0096** | 0.0699 | **0.0096** | 1.5818 | | 0.9947 | 0.9948 | 8.1801 | | 0.9954 | 0.9952 |  |
|  |  | 32 | 0.0015 | 0.0013 | 0.0012 | 7.4379 | | 7.4576 | 7.4574 | 7.4629 | 7.4518 | | 7.4527 | 0.0108 | 0.0013 | 0.0108 | **0.001** | 1.6589 | | 1.0033 | 1.0036 | 8.3408 | | 1.0032 | 1.0031 |  |
|  |  | 40 | **0.003** | **0.003** | **0.003** | 7.4246 | | 7.4182 | 7.4181 | 7.4204 | 7.3934 | | 7.4132 | 0.053 | 0.0043 | 0.053 | 0.0042 | 1.755 | | 1.0064 | 1.0061 | 8.6301 | | 1.0062 | 1.0062 |  |
|  |  | 48 | 0.0066 | 0.0066 | 0.0066 | 7.3029 | | 7.3022 | 7.3021 | 7.3030 | 7.3325 | | 7.3029 | 0.0294 | 0.0064 | 0.0294 | **0.0063** | 2.1226 | | 0.9975 | 0.9972 | 9.1044 | | 0.9972 | 0.9972 |  |
|  |  | 56 | 0.0018 | 0.0018 | 0.0019 | 7.2397 | | 7.2528 | 7.2529 | 7.2509 | 7.2625 | | 7.2572 | 0.0353 | **0.0011** | 0.0353 | 0.0012 | 1.8216 | | 0.9971 | 0.9971 | 6.1678 | | 0.9985 | 0.9984 |  |
|  |  | 72 | 0.0015 | 0.0015 | 0.0015 | 7.086 | | 7.0842 | 7.0842 | 7.0873 | 7.0831 | | 7.0802 | 0.0201 | 0.0015 | 0.0201 | **0.0014** | 1.6045 | | 1.0024 | 1.0026 | 8.6273 | | 1.0022 | 1.0022 |  |
|  |  | 88 | **0.0005** | **0.0005** | **0.0005** | 7.017 | | 7.0284 | 7.0285 | 7.0270 | 7.0419 | | 7.03 | 0.0413 | 0.0007 | 0.0413 | 0.0007 | 1.8534 | | 0.999 | 0.999 | 8.9866 | | 0.9987 | 0.9987 |  |
|  |  | 120 | **0.007** | **0.007** | **0.007** | 7.367 | | 7.369 | 7.369 | 7.3703 | 7.3854 | | 7.3672 | 0.0492 | 0.0072 | 0.0492 | 0.0073 | 1.9911 | | 1.0037 | 1.0038 | 9.719 | | 1.0031 | 1.0031 |  |
|  |  | 184 | **0.001** | **0.001** | 0.0011 | 7.1089 | | 7.1148 | 7.1148 | 7.1147 | 7.1091 | | 7.115 | 0.0227 | 0.0011 | 0.0227 | 0.0011 | 1.8857 | | 1.0004 | 1.0003 | 7.1826 | | 1.0003 | 1.0003 |  |
|  |  | 312 | **0.0004** | **0.0004** | **0.0004** | 6.8628 | | 6.8634 | 6.8634 | 6.8632 | 6.848 | | 6.8638 | 0.051 | 0.0005 | 0.051 | 0.0005 | 1.8733 | | 0.999 | 0.999 | 9.0329 | | 0.9989 | 0.9989 |  |
| Heteroscedastic (control higher variance) | Parallel trend | 24 | 0.313 | 0.3207 | 0.3325 | 0.082 | | 0.0836 | 0.0891 | 0.0836 | **0.0655** | | 0.0939 | 0.287 | 0.3321 | 0.287 | 0.3344 | 1.0896 | | 1.1144 | 1.1079 | 1.0871 | | 1.1187 | 1.114 |  |
|  |  | 32 | 0.0518 | 0.0536 | 0.0584 | 0.0581 | | 0.0558 | 0.0559 | 0.0617 | 0.0581 | | **0.0276** | 0.0633 | 0.0394 | 0.0633 | 0.0281 | 0.9727 | | 0.8349 | 0.8358 | 0.9846 | | 0.8571 | 0.8563 |  |
|  |  | 40 | 0.2358 | 0.2354 | 0.2387 | 0.0636 | | 0.0656 | 0.0645 | 0.0597 | **0.0431** | | 0.053 | 0.2936 | 0.2398 | 0.2936 | 0.2393 | 0.9857 | | 0.8766 | 0.8779 | 0.9907 | | 0.8747 | 0.8757 |  |
|  |  | 48 | 0.0936 | 0.0934 | 0.0955 | 0.017 | | 0.0162 | 0.0179 | 0.0154 | 0.0522 | | **0.0083** | 0.1366 | 0.0709 | 0.1366 | 0.0696 | 1.0389 | | 0.9879 | 0.9904 | 1.0426 | | 0.9833 | 0.9855 |  |
|  |  | 56 | 0.2031 | 0.2038 | 0.1984 | 0.0748 | | 0.074 | 0.0735 | 0.0699 | **0.0635** | | 0.0814 | 0.2179 | 0.2097 | 0.2179 | 0.2126 | 0.9932 | | 1.062 | 1.0642 | 0.9836 | | 1.0517 | 1.0532 |  |
|  |  | 72 | 0.0701 | 0.0704 | 0.073 | 0.0129 | | 0.0135 | 0.0133 | 0.0128 | **0.0038** | | 0.0135 | 0.102 | 0.0666 | 0.102 | 0.0658 | 1.0135 | | 0.9899 | 0.9887 | 1.0192 | | 1.0094 | 1.0089 |  |
|  |  | 88 | 0.1362 | 0.1357 | 0.1361 | 0.0329 | | 0.0336 | 0.0329 | **0.0290** | 0.0441 | | 0.0324 | 0.1897 | 0.1389 | 0.1897 | 0.1395 | 0.9755 | | 0.9278 | 0.9279 | 0.9767 | | 0.9268 | 0.9273 |  |
|  |  | 120 | 0.0334 | 0.0334 | 0.036 | 0.0639 | | 0.0637 | 0.0636 | 0.0640 | 0.0788 | | 0.0661 | **0.0027** | 0.022 | **0.0027** | 0.0226 | 1.0066 | | 1.0448 | 1.0449 | 1.0077 | | 1.048 | 1.048 |  |
|  |  | 184 | 0.0268 | 0.0265 | 0.0256 | 0.0257 | | 0.0258 | 0.0257 | 0.0251 | 0.0182 | | 0.0272 | **0.0061** | 0.0265 | **0.0061** | 0.0263 | 1.0018 | | 1.0265 | 1.0261 | 1.0024 | | 1.0238 | 1.0236 |  |
|  |  | 312 | 0.0172 | 0.0172 | 0.0192 | 0.0003 | | **0.0002** | **0.0002** | 0.0019 | 0.0147 | | 0.0032 | 0.0677 | 0.0194 | 0.0677 | 0.0199 | 0.9897 | | 0.9676 | 0.9676 | 0.9908 | | 0.9682 | 0.9682 |  |
|  | Unparallel trend | 24 | 0.4807 | 0.4904 | 0.5115 | 6.4731 | | 6.4583 | 6.4504 | 6.4542 | 6.4704 | | 6.4485 | 0.4715 | 0.5171 | 0.4715 | 0.521 | **0.2115** | | 1.1678 | 1.1584 | 1.7526 | | 1.182 | 1.175 |  |
|  |  | 32 | 0.1028 | 0.1053 | 0.1149 | 7.3805 | | 7.3643 | 7.3635 | 7.3535 | 7.3605 | | 7.4043 | 0.119 | 0.0849 | 0.119 | **0.0688** | 0.4838 | | 0.7375 | 0.7386 | 0.8618 | | 0.778 | 0.7767 |  |
|  |  | 40 | 0.3739 | 0.3729 | 0.3796 | 7.2844 | | 7.2877 | 7.2891 | 7.2971 | 7.311 | | 7.3028 | 0.434 | 0.3795 | 0.434 | 0.3795 | **0.2763** | | 0.8114 | 0.8137 | 1.2075 | | 0.8036 | 0.805 |  |
|  |  | 48 | 0.1566 | 0.1563 | 0.1608 | 7.275 | | 7.2769 | 7.2742 | 7.2789 | 7.2369 | | 7.2891 | 0.2051 | 0.1236 | 0.2051 | **0.1215** | 0.2135 | | 0.9821 | 0.9855 | 1.8639 | | 0.9728 | 0.9764 |  |
|  |  | 56 | 0.2874 | 0.2882 | **0.2827** | 7.3595 | | 7.3449 | 7.3443 | 7.3383 | 7.3329 | | 7.361 | 0.2971 | 0.2968 | 0.2971 | 0.3017 | 1.438 | | 1.0995 | 1.1025 | 1.1011 | | 1.0793 | 1.0816 |  |
|  |  | 72 | 0.1078 | 0.1083 | 0.1117 | 7.0757 | | 7.0764 | 7.0767 | 7.0792 | 7.0918 | | 7.0741 | 0.1452 | 0.1039 | 0.1452 | **0.1029** | 0.1654 | | 0.9848 | 0.9827 | 1.5787 | | 1.0154 | 1.0146 |  |
|  |  | 88 | 0.2087 | 0.208 | 0.2079 | 6.9758 | | 6.9632 | 6.9643 | 6.9691 | 6.9532 | | 6.9663 | 0.2679 | 0.2137 | 0.2679 | 0.2149 | **0.2074** | | 0.8874 | 0.8871 | 1.9194 | | 0.8831 | 0.884 |  |
|  |  | 120 | 0.0488 | 0.0488 | 0.0525 | 7.2791 | | 7.2775 | 7.2777 | 7.2771 | 7.2633 | | 7.2723 | **0.0156** | 0.0296 | **0.0156** | 0.0304 | 0.6645 | | 1.0655 | 1.0654 | 2.3629 | | 1.0719 | 1.072 |  |
|  |  | 184 | 0.0499 | 0.0495 | 0.0484 | 7.086 | | 7.0799 | 7.08 | 7.0813 | 7.0882 | | 7.0785 | **0.0302** | 0.0499 | **0.0302** | 0.0496 | 0.5965 | | 1.0413 | 1.0408 | 0.0715 | | 1.0367 | 1.0364 |  |
|  |  | 312 | **0.023** | **0.023** | 0.0266 | 6.8581 | | 6.8574 | 6.8573 | 6.8597 | 6.8715 | | 6.8624 | 0.0742 | 0.0269 | 0.0742 | 0.0276 | 0.4536 | | 0.9495 | 0.9495 | 2.1548 | | 0.9508 | 0.9507 |  |
| Heteroscedastic (intervention higher variance) | Parallel trend | 24 | 0.3363 | 0.3279 | 0.3204 | 0.0905 | | 0.0895 | 0.0838 | 0.0889 | 0.0948 | | **0.0729** | 0.3619 | 0.2784 | 0.3619 | 0.2799 | 0.9679 | | 0.7954 | 0.7878 | 1.0106 | | 0.7885 | 0.7926 |  |
|  |  | 32 | 0.0468 | 0.0449 | 0.0567 | 0.0559 | | 0.0575 | 0.0562 | 0.0614 | 0.0853 | | 0.0314 | 0.0419 | 0.0337 | 0.0419 | **0.022** | 0.9109 | | 0.7934 | 0.8014 | 0.9272 | | 0.7667 | 0.7731 |  |
|  |  | 40 | 0.2352 | 0.2352 | 0.2403 | 0.0702 | | 0.0681 | 0.0693 | 0.0670 | 0.1053 | | **0.0613** | 0.1788 | 0.2337 | 0.1788 | 0.2336 | 0.9394 | | 1.1501 | 1.1555 | 0.9378 | | 1.1339 | 1.1371 |  |
|  |  | 48 | 0.1137 | 0.1131 | 0.1148 | 0.0216 | | 0.022 | 0.0202 | 0.0223 | **0.0009** | | 0.0146 | 0.096 | 0.1013 | 0.096 | 0.1001 | 1.031 | | 0.8758 | 0.881 | 1.0152 | | 0.8429 | 0.8454 |  |
|  |  | 56 | 0.1853 | 0.185 | 0.1936 | 0.08 | | 0.0809 | 0.0816 | **0.0785** | 0.0887 | | 0.0818 | 0.1505 | 0.209 | 0.1505 | 0.2123 | 1.0659 | | 0.8376 | 0.8392 | 1.0597 | | 0.8572 | 0.8543 |  |
|  |  | 72 | 0.0723 | 0.0717 | 0.072 | 0.0053 | | 0.0047 | 0.0052 | **0.0036** | 0.0099 | | 0.0115 | 0.0523 | 0.0609 | 0.0523 | 0.0602 | 1.0148 | | 1.075 | 1.0756 | 1.0186 | | 1.0729 | 1.0722 |  |
|  |  | 88 | 0.1571 | 0.1573 | 0.1511 | 0.0331 | | 0.0325 | 0.0332 | 0.0316 | **0.0183** | | 0.0368 | 0.1099 | 0.1451 | 0.1099 | 0.1457 | 0.9354 | | 0.7687 | 0.7725 | 0.9435 | | 0.775 | 0.777 |  |
|  |  | 120 | 0.0305 | 0.0304 | 0.0357 | 0.0563 | | 0.0565 | 0.0567 | 0.0560 | 0.0397 | | 0.057 | 0.0802 | **0.0203** | 0.0802 | 0.0209 | 0.9589 | | 1.0957 | 1.0972 | 0.9578 | | 1.0942 | 1.0951 |  |
|  |  | 184 | 0.0232 | 0.0235 | 0.0246 | 0.0232 | | 0.0231 | 0.0232 | 0.0227 | 0.0283 | | 0.026 | 0.0451 | 0.0221 | 0.0451 | **0.0219** | 0.9833 | | 1.0024 | 1.003 | 0.9782 | | 0.9993 | 0.9989 |  |
|  |  | 312 | 0.0168 | 0.0168 | 0.0141 | 0.0009 | | 0.0008 | 0.0008 | 0.0025 | 0.0181 | | **0.0006** | 0.0358 | 0.017 | 0.0358 | 0.0174 | 0.9792 | | 0.9748 | 0.9752 | 0.9896 | | 0.9845 | 0.9848 |  |
|  | Unparallel trend | 24 | 0.5664 | 0.5566 | 0.5422 | 6.4129 | | 6.4265 | 6.4347 | 6.4280 | 6.4295 | | 6.4567 | 0.5673 | **0.4882** | 0.5673 | 0.4917 | 4.876 | | 0.6568 | 0.6406 | 8.1893 | | 0.6302 | 0.6336 |  |
|  |  | 32 | 0.1009 | 0.0986 | 0.1152 | 7.3551 | | 7.3729 | 7.3751 | 7.3709 | 7.3402 | | 7.408 | 0.094 | 0.0761 | 0.094 | **0.0596** | 4.919 | | 0.6553 | 0.6604 | 8.2347 | | 0.6018 | 0.6112 |  |
|  |  | 40 | 0.3849 | 0.3853 | 0.3908 | 7.2989 | | 7.2955 | 7.2939 | 7.2974 | 7.2541 | | 7.3 | **0.3268** | 0.3827 | **0.3268** | 0.3829 | 5.4837 | | 1.2552 | 1.2662 | 8.5153 | | 1.2232 | 1.2286 |  |
|  |  | 48 | 0.1615 | 0.1607 | 0.1622 | 7.2792 | | 7.278 | 7.2806 | 7.2786 | 7.2982 | | 7.2912 | 0.152 | 0.1423 | 0.152 | **0.1402** | 5.4112 | | 0.8246 | 0.8297 | 9.1374 | | 0.7692 | 0.7723 |  |
|  |  | 56 | 0.2582 | 0.2579 | 0.2711 | 7.342 | | 7.3568 | 7.3578 | 7.3511 | 7.3598 | | 7.3636 | **0.2214** | 0.288 | **0.2214** | 0.2932 | 4.717 | | 0.7902 | 0.7918 | 6.238 | | 0.8054 | 0.8011 |  |
|  |  | 72 | 0.1192 | 0.1184 | 0.1183 | 7.0894 | | 7.0885 | 7.088 | 7.0899 | 7.0841 | | 7.0747 | **0.0992** | 0.1013 | **0.0992** | 0.1003 | 5.5181 | | 1.1291 | 1.1293 | 8.6665 | | 1.12 | 1.1188 |  |
|  |  | 88 | 0.2342 | 0.2346 | 0.2246 | 6.9583 | | 6.9708 | 6.9698 | 6.9718 | 6.9836 | | 6.9662 | **0.1839** | 0.215 | **0.1839** | 0.2163 | 5.0314 | | 0.6472 | 0.6529 | 8.8868 | | 0.6564 | 0.6595 |  |
|  |  | 120 | 0.0441 | 0.0441 | 0.0531 | 7.2809 | | 7.2825 | 7.2822 | 7.2846 | 7.3022 | | 7.2809 | 0.0997 | **0.0291** | 0.0997 | 0.0298 | 6.1024 | | 1.1361 | 1.1384 | 9.6535 | | 1.1358 | 1.1373 |  |
|  |  | 184 | 0.0519 | 0.0523 | 0.0541 | 7.0755 | | 7.0817 | 7.0815 | 7.0818 | 7.0763 | | 7.0777 | 0.0736 | 0.05 | 0.0736 | **0.0497** | 5.2824 | | 0.9802 | 0.9805 | 7.1506 | | 0.9826 | 0.9819 |  |
|  |  | 312 | 0.0278 | 0.0278 | **0.0235** | 6.8583 | | 6.859 | 6.8591 | 6.8566 | 6.8402 | | 6.8614 | 0.026 | 0.0276 | 0.026 | 0.0283 | 5.4355 | | 0.9675 | 0.968 | 9.0146 | | 0.9791 | 0.9796 |  |

*Panel FE and DK have the same bias as the difference in the statistical regression only concerns standard errors. Colours rank the performance relative to each sample size, from green (bias closest to 0 -optimal situation) to red (bias furthest from 0). Figures in bold represent the lowest bias related to the specific sample size. Abbreviations follow notation of Table 2 in the main text.

## A1b. Bias Models with autocorrelated errors

| Heteroscedastic scenario | Parallel scenario | # data points | | **Controlled segmented regression (CITS)** | | | **DiD framework segmented regression** | | | | **ITS of the difference** | | | **control as covariate** | | | **Uncontrolled ITS** | | |
| --- | --- | --- | --- | --- | --- | --- | --- | --- | --- | --- | --- | --- | --- | --- | --- | --- | --- | --- | --- |
|  |  |  |  | panel FE/DK* T | Panel FE splnT | panel FGLS T | panel FE/DK* | panel FE/DK* T | Panel FE splnT | panel FGLS T | diff X | diff X T | diff X splnT | Ols C | Ols C T | ols C splnT | Ols | Ols T | ols splnT |
| Homoscedastic | Parallel trend | | 24 | 0.0125 | 0.0125 | 0.0131 | 0.0027 | 0.0028 | 0.0028 | 0.0029 | **0.0026** | 0.0122 | 0.0121 | 0.9958 | 0.9882 | 0.9880 | 0.9971 | 0.9881 | 0.9895 |
|  |  |  | 32 | 0.0083 | 0.0082 | 0.0081 | 0.0071 | 0.0071 | 0.0071 | 0.0070 | **0.0069** | 0.0076 | 0.0073 | 1.0042 | 1.0006 | 1.0006 | 1.0038 | 1.0011 | 1.0008 |
|  |  |  | 40 | 0.0045 | 0.0045 | 0.0045 | 0.0018 | 0.0018 | **0.0017** | 0.0018 | 0.0020 | 0.0059 | 0.0057 | 1.0048 | 1.0115 | 1.0112 | 1.0043 | 1.0119 | 1.0116 |
|  |  |  | 48 | 0.0084 | 0.0085 | 0.0083 | 0.0081 | 0.0082 | 0.0080 | 0.0082 | 0.0084 | **0.0068** | 0.0083 | 0.9915 | 0.9938 | 0.9932 | 0.9909 | 0.9926 | 0.9925 |
|  |  |  | 56 | 0.0032 | 0.0031 | 0.0034 | **0.0013** | 0.0014 | 0.0014 | 0.0014 | 0.0014 | 0.0031 | 0.0032 | 0.9961 | 0.9903 | 0.9905 | 0.9971 | 0.9926 | 0.9929 |
|  |  |  | 72 | 0.0027 | 0.0028 | 0.0029 | 0.0008 | 0.0008 | 0.0008 | **0.0007** | 0.0009 | 0.0030 | 0.0030 | 0.9996 | 1.0061 | 1.0064 | 0.9993 | 1.0050 | 1.0051 |
|  |  |  | 88 | 0.0022 | 0.0022 | 0.0022 | 0.0013 | 0.0013 | **0.0013** | 0.0013 | 0.0014 | 0.0025 | 0.0025 | 1.0006 | 0.9993 | 0.9989 | 1.0007 | 0.9994 | 0.9993 |
|  |  |  | 120 | 0.0130 | 0.0131 | 0.0131 | 0.0006 | 0.0008 | 0.0006 | 0.0007 | **0.0005** | 0.0128 | 0.0132 | 1.0002 | 1.0042 | 1.0043 | 0.9996 | 1.0037 | 1.0038 |
|  |  |  | 184 | 0.0014 | 0.0014 | 0.0014 | 0.0008 | 0.0008 | 0.0008 | 0.0008 | **0.0008** | 0.0014 | 0.0014 | 1.0008 | 0.9992 | 0.9992 | 1.0011 | 0.9995 | 0.9995 |
|  |  |  | 312 | 0.0010 | 0.0010 | 0.0010 | **0.0006** | 0.0006 | 0.0006 | 0.0006 | 0.0006 | 0.0010 | 0.0010 | 0.9986 | 0.9974 | 0.9973 | 0.9987 | 0.9975 | 0.9975 |
|  | Unparallel trend | | 24 | 0.0125 | 0.0125 | 0.0131 | 6.5663 | 6.5790 | 6.5790 | 6.5786 | 6.5827 | 0.0122 | **0.0121** | 1.5620 | 0.9882 | 0.9880 | 7.2740 | 0.9881 | 0.9895 |
|  |  |  | 32 | 0.0083 | 0.0082 | 0.0081 | 7.4423 | 7.4619 | 7.4617 | 7.4673 | 7.4567 | 0.0076 | **0.0073** | 1.7700 | 1.0006 | 1.0006 | 7.4787 | 1.0011 | 1.0008 |
|  |  |  | 40 | 0.0045 | **0.0045** | 0.0045 | 7.4254 | 7.4190 | 7.4188 | 7.4213 | 7.4138 | 0.0059 | 0.0057 | 1.9623 | 1.0115 | 1.0112 | 7.7974 | 1.0119 | 1.0116 |
|  |  |  | 48 | 0.0084 | 0.0085 | **0.0083** | 7.2875 | 7.2994 | 7.2862 | 7.3001 | 7.2873 | 0.0084 | 0.0093 | 2.4286 | 0.9933 | 0.9921 | 8.3206 | 0.9924 | 0.9917 |
|  |  |  | 56 | 0.0032 | 0.0031 | 0.0034 | 7.2403 | 7.2535 | 7.2535 | 7.2515 | 7.2580 | **0.0031** | 0.0032 | 2.2072 | 0.9903 | 0.9905 | 5.6626 | 0.9926 | 0.9929 |
|  |  |  | 72 | **0.0027** | 0.0028 | 0.0029 | 7.0858 | 7.0840 | 7.0840 | 7.0871 | 7.0800 | 0.0030 | 0.0030 | 1.9515 | 1.0061 | 1.0064 | 7.9945 | 1.0050 | 1.0051 |
|  |  |  | 88 | 0.0022 | **0.0022** | 0.0022 | 7.0162 | 7.0276 | 7.0276 | 7.0262 | 7.0291 | 0.0025 | 0.0025 | 2.2309 | 0.9993 | 0.9989 | 8.3684 | 0.9994 | 0.9993 |
|  |  |  | 120 | **0.0130** | 0.0131 | 0.0131 | 7.3670 | 7.3689 | 7.3689 | 7.3703 | 7.3671 | 0.0131 | 0.0132 | 2.5789 | 1.0043 | 1.0043 | 9.1162 | 1.0038 | 1.0038 |
|  |  |  | 184 | **0.0014** | 0.0014 | 0.0014 | 7.1094 | 7.1153 | 7.1153 | 7.1152 | 7.1155 | 0.0014 | 0.0014 | 2.6936 | 0.9992 | 0.9992 | 6.8344 | 0.9995 | 0.9995 |
|  |  |  | 312 | 0.0010 | 0.0010 | 0.0010 | 6.8625 | 6.8631 | 6.8631 | 6.8629 | 6.8635 | **0.0010** | 0.0010 | 2.4905 | 0.9974 | 0.9973 | 8.6785 | 0.9975 | 0.9975 |
| Heteroscedastic (control higher variance) | Parallel trend | | 24 | 0.2059 | 0.2164 | 0.2077 | 0.0725 | 0.0737 | 0.0812 | **0.0700** | 0.0813 | 0.1721 | 0.1737 | 1.1275 | 1.2039 | 1.1597 | 1.0994 | 1.1780 | 1.1544 |
|  |  |  | 32 | 0.0079 | 0.0048 | **0.0035** | 0.0794 | 0.0764 | 0.0767 | 0.0843 | 0.0486 | 0.0334 | 0.0464 | 0.9683 | 0.8480 | 0.8552 | 0.9816 | 0.8498 | 0.8542 |
|  |  |  | 40 | 0.5316 | 0.5308 | 0.5435 | 0.0741 | 0.0772 | 0.0753 | 0.0683 | **0.0587** | 0.5264 | 0.5252 | 0.9542 | 0.7297 | 0.7319 | 0.9765 | 0.7567 | 0.7482 |
|  |  |  | 48 | 0.2208 | 0.2209 | 0.2206 | 0.0419 | 0.0404 | 0.0430 | 0.0417 | **0.0228** | 0.1868 | 0.1845 | 1.1025 | 1.1230 | 1.1216 | 1.1116 | 1.1018 | 1.0942 |
|  |  |  | 56 | 0.3544 | 0.3549 | 0.3571 | 0.1054 | 0.1041 | 0.1034 | **0.0983** | 0.1141 | 0.3574 | 0.3631 | 0.9803 | 1.0872 | 1.0941 | 0.9743 | 1.1064 | 1.1112 |
|  |  |  | 72 | 0.0876 | 0.0884 | 0.0913 | 0.0037 | 0.0047 | 0.0044 | **0.0018** | 0.0046 | 0.0877 | 0.0866 | 1.0067 | 0.9186 | 0.9241 | 1.0147 | 0.9484 | 0.9519 |
|  |  |  | 88 | 0.2484 | 0.2477 | 0.2443 | 0.0578 | 0.0589 | 0.0577 | **0.0538** | 0.0626 | 0.2598 | 0.2608 | 0.9485 | 0.8659 | 0.8672 | 0.9483 | 0.8590 | 0.8603 |
|  |  |  | 120 | 0.0804 | 0.0804 | 0.0831 | 0.0965 | 0.0961 | 0.0958 | 0.0963 | 0.0974 | **0.0740** | 0.0754 | 1.0112 | 1.0549 | 1.0550 | 1.0147 | 1.0564 | 1.0571 |
|  |  |  | 184 | 0.0502 | 0.0498 | 0.0491 | 0.0414 | 0.0416 | **0.0414** | 0.0414 | 0.0446 | 0.0519 | 0.0513 | 1.0015 | 1.0282 | 1.0277 | 1.0026 | 1.0276 | 1.0271 |
|  |  |  | 312 | 0.0261 | 0.0261 | 0.0280 | 0.0031 | 0.0032 | 0.0033 | 0.0016 | **0.0000** | 0.0295 | 0.0303 | 0.9805 | 0.9373 | 0.9372 | 0.9818 | 0.9376 | 0.9375 |
|  | Unparallel trend | | 24 | 0.6822 | 0.7004 | 0.6868 | 0.0031 | 6.3904 | 0.0033 | 6.3851 | **0.0000** | 0.6804 | 0.0303 | 0.9805 | 1.3843 | 0.9372 | 0.9818 | 1.3650 | 0.9375 |
|  |  |  | 32 | 0.0254 | 0.0300 | 0.0329 | 7.3419 | 7.3266 | 7.3253 | 7.3122 | 7.3647 | **0.0097** | 0.0283 | 0.0829 | 0.7746 | 0.8152 | 0.9075 | 0.7730 | 0.7797 |
|  |  |  | 40 | 0.8368 | 0.8351 | 0.8569 | 7.2502 | 7.2519 | 7.2542 | 7.2668 | 7.2766 | 0.8266 | 0.8257 | **0.1921** | 0.5926 | 0.6022 | 1.1941 | 0.6206 | 0.6107 |
|  |  |  | 48 | 0.3616 | 0.3616 | 0.3612 | 7.2292 | 7.2321 | 7.2280 | 7.2310 | 7.2591 | 0.3128 | **0.3090** | 0.9054 | 1.1937 | 1.1804 | 1.9363 | 1.1522 | 1.1427 |
|  |  |  | 56 | **0.5059** | 0.5065 | 0.5120 | 7.3939 | 7.3785 | 7.3776 | 7.3693 | 7.3995 | 0.5080 | 0.5175 | 1.1213 | 1.1525 | 1.1638 | 0.8911 | 1.1641 | 1.1713 |
|  |  |  | 72 | **0.1424** | 0.1436 | 0.1476 | 7.0944 | 7.0944 | 7.0949 | 7.1007 | 7.0921 | 0.1444 | 0.1430 | 0.7441 | 0.8676 | 0.8773 | 1.5326 | 0.9196 | 0.9249 |
|  |  |  | 88 | 0.3857 | 0.3846 | **0.3780** | 6.9285 | 6.9153 | 6.9172 | 6.9222 | 6.9114 | 0.4024 | 0.4046 | 0.8404 | 0.7921 | 0.7933 | 1.8558 | 0.7775 | 0.7794 |
|  |  |  | 120 | 0.1218 | 0.1219 | 0.1252 | 7.2318 | 7.2306 | 7.2310 | 7.2302 | 7.2273 | **0.1116** | 0.1135 | 1.4389 | 1.0743 | 1.0741 | 2.3418 | 1.0828 | 1.0840 |
|  |  |  | 184 | 0.0924 | 0.0917 | **0.0911** | 7.0658 | 7.0595 | 7.0599 | 7.0601 | 7.0554 | 0.0965 | 0.0957 | 0.2551 | 1.0452 | 1.0444 | 0.1513 | 1.0420 | 1.0413 |
|  |  |  | 312 | 0.0343 | **0.0343** | 0.0375 | 6.8491 | 6.8484 | 6.8483 | 6.8508 | 6.8536 | 0.0399 | 0.0411 | 1.2223 | 0.9015 | 0.9014 | 2.1492 | 0.9030 | 0.9028 |
| Heteroscedastic (intervention higher variance) | Parallel trend | | 24 | 0.3890 | 0.3743 | 0.3829 | 0.1348 | 0.1346 | 0.1251 | 0.1244 | **0.1156** | 0.3638 | 0.3656 | 1.0188 | 0.8927 | 0.9650 | 1.0625 | 0.9120 | 0.9133 |
|  |  |  | 32 | 0.0464 | 0.0496 | **0.0276** | 0.0663 | 0.0692 | 0.0676 | 0.0719 | 0.0529 | 0.0372 | 0.0508 | 0.9005 | 0.8690 | 0.8475 | 0.9374 | 0.8111 | 0.7733 |
|  |  |  | 40 | 0.5278 | 0.5277 | 0.5332 | 0.0788 | 0.0758 | 0.0778 | 0.0774 | **0.0713** | 0.5187 | 0.5181 | 0.9688 | 1.2070 | 1.2209 | 0.9495 | 1.1536 | 1.1978 |
|  |  |  | 48 | 0.2504 | 0.2495 | 0.2512 | 0.0423 | 0.0433 | 0.0404 | 0.0450 | **0.0341** | 0.2438 | 0.2416 | 1.0166 | 0.6425 | 0.6987 | 0.9665 | 0.5850 | 0.6154 |
|  |  |  | 56 | 0.3541 | 0.3538 | 0.3572 | **0.1089** | 0.1102 | 0.1113 | 0.1105 | 0.1160 | 0.3545 | 0.3609 | 1.0787 | 0.8321 | 0.8245 | 1.0881 | 0.8262 | 0.8151 |
|  |  |  | 72 | 0.0910 | 0.0900 | 0.0886 | 0.0071 | 0.0080 | 0.0074 | 0.0095 | **0.0016** | 0.0801 | 0.0792 | 1.0360 | 1.1443 | 1.1608 | 1.0316 | 1.1466 | 1.1655 |
|  |  |  | 88 | 0.2630 | 0.2635 | 0.2560 | 0.0672 | 0.0662 | 0.0675 | **0.0636** | 0.0710 | 0.2696 | 0.2708 | 0.8989 | 0.6343 | 0.6547 | 0.9038 | 0.6425 | 0.6452 |
|  |  |  | 120 | 0.0671 | 0.0671 | 0.0719 | 0.0852 | 0.0871 | 0.0858 | 0.0875 | 0.0812 | 0.0712 | **0.0663** | 0.9449 | 1.2171 | 1.2025 | 0.9471 | 1.2132 | 1.1997 |
|  |  |  | 184 | 0.0425 | 0.0430 | 0.0438 | 0.0413 | 0.0411 | 0.0413 | **0.0403** | 0.0431 | 0.0458 | 0.0453 | 0.9729 | 1.0038 | 1.0037 | 0.9686 | 1.0058 | 1.0051 |
|  |  |  | 312 | 0.0221 | 0.0222 | 0.0198 | 0.0064 | 0.0064 | 0.0062 | 0.0080 | **0.0041** | 0.0262 | 0.0270 | 0.9673 | 0.9534 | 0.9537 | 0.9796 | 0.9646 | 0.9649 |
|  | Unparallel trend | | 24 | 0.6748 | 0.6565 | 0.6666 | 6.3385 | 6.3507 | 6.3643 | 6.3628 | 6.3868 | **0.6469** | 0.6511 | 5.0019 | 0.9421 | 0.9830 | 7.1575 | 0.7076 | 0.7667 |
|  |  |  | 32 | 0.0299 | 0.0341 | **0.0030** | 7.3368 | 7.3530 | 7.3557 | 7.3535 | 7.3733 | 0.0202 | 0.0391 | 5.4545 | 0.7607 | 0.7911 | 7.3012 | 0.7142 | 0.6118 |
|  |  |  | 40 | 0.8476 | 0.8480 | 0.8558 | 7.2737 | 7.2720 | 7.2689 | 7.2717 | 7.2730 | 0.8408 | **0.8321** | 5.9070 | 1.3029 | 1.3046 | 7.7999 | 1.2387 | 1.2739 |
|  |  |  | 48 | 0.3565 | 0.3553 | 0.3564 | 7.2475 | 7.2414 | 7.2497 | 7.2412 | 7.2608 | 0.3573 | 0.3444 | 6.0942 | 0.4159 | 0.5269 | 8.3086 | **0.3269** | 0.3977 |
|  |  |  | 56 | 0.4981 | 0.4978 | 0.5042 | 7.3745 | 7.3900 | 7.3916 | 7.3882 | 7.4042 | **0.4933** | 0.5033 | 5.1156 | 0.7502 | 0.7694 | 5.7439 | 0.7693 | 0.7566 |
|  |  |  | 72 | 0.1585 | 0.1572 | 0.1541 | 7.1115 | 7.1113 | 7.1105 | 7.1140 | 7.0927 | 0.1414 | **0.1402** | 6.5326 | 1.2368 | 1.2657 | 8.1370 | 1.2479 | 1.2588 |
|  |  |  | 88 | 0.3927 | 0.3935 | 0.3819 | 6.9033 | 6.8935 | 6.9145 | 6.8968 | 6.9110 | 0.4173 | 0.4051 | 6.0729 | **0.3749** | 0.4014 | 8.3642 | 0.4461 | 0.4519 |
|  |  |  | 120 | 0.1028 | **0.1028** | 0.1109 | 7.2338 | 7.2351 | 7.2346 | 7.2355 | 7.2416 | 0.1107 | 0.1125 | 7.5748 | 1.3170 | 1.3168 | 9.2852 | 1.3104 | 1.3083 |
|  |  |  | 184 | **0.0925** | 0.0931 | 0.0941 | 7.0497 | 7.0560 | 7.0556 | 7.0568 | 7.0539 | 0.0976 | 0.0969 | 6.2494 | 0.9660 | 0.9654 | 6.9459 | 0.9745 | 0.9733 |
|  |  |  | 312 | 0.0356 | 0.0356 | **0.0318** | 6.8477 | 6.8484 | 6.8486 | 6.8460 | 6.8520 | 0.0419 | 0.0431 | 7.0375 | 0.9330 | 0.9334 | 8.8419 | 0.9480 | 0.9484 |

*Panel FE and DK have the same bias as the difference in the statistical regression only concerns standard errors. Colours rank the performance relative to each sample size, from green (bias closest to 0 -optimal situation) to red (bias furthest from 0). Figures in bold represent the lowest bias related to the specific sample size. Abbreviations follow notation of Table 2 in the main text. Control covariate and uncontrolled ITS models in this scenario include ARMA components for autocorrelated error structure

## A2. Bias- error autocorrelated scenario.*

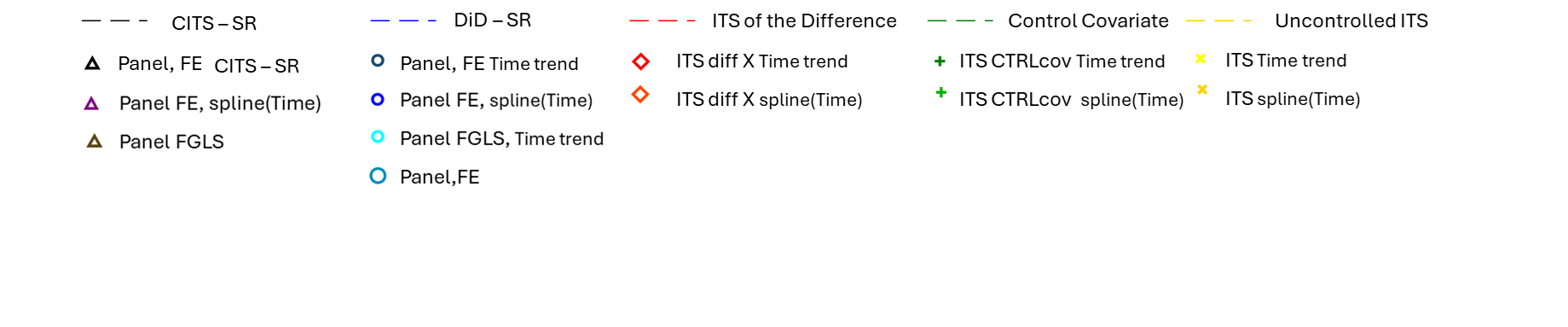


*= Control covariate and uncontrolled ITS models in this scenario include ARMA components for autocorrelated error structure

## A3. DGP correspondence with motivating example

| **Feature** | **Motivating example** | **DGP** |
| --- | --- | --- |
| n. of Treatment and Control groups | 1 treated group (Glasgow), 26 controls (all Scottish City Councils except those with low populations and opiate deaths). | 1 treatment, 1 control group (Choosing features from most similar control group -Edinburgh- from the motivating example) |
| Type of dependent variable | Count and rate | Continuous |
| Average difference between treatment and control groups | 7.32 | 7.3 |
| n. of independent variables | 2 | 1 |
| Mean and sd of treatment group | 10.335 , 3.64 | 10 , 3.5 (mean and s.d. of main covariate) |
| Correlation between series | 0.2 | 0.2 (correlation of the two independent variables, final correlations of the series is higher) |
| # Pre and post intervention observations | 120 fortnightly observations:  82 (pre) and 38 (post) | 24: 12 (pre), 12 (post) 32: 16 (pre), 16 (post) 40: 20 (pre), 20 (post) 48: 24 (pre), 24(post) 56: 28 (pre), 28 (post) 72: 36 (pre), 36 (post) 88: 44 (pre), 44 (post) 120: 60 (pre), 60 (post)  184: 92 (pre), 92 (post) 312: 156 (pre), 156 (post) |
| Error autocorrelation ρ coefficient | 0.27,0.28 | Base case: 0  autocorrelated error scenario: 0.7 |
| Sd of the model error | 2.16(for count -panel poisson regression-), .59(for rates -panel Fe-) | 0.1 |
| Estimated intervention effect | .96 (IRR -poisson panel model) continuous model using panel linear regression: -1.63 | -2 |

## A4. OLS regression on bias

CITS segmented regression

-----------------------------------------------------------------------------------

**Panel FE** | Coefficient Std. err. t P>|t| [95% conf. interval]

------------------+----------------------------------------------------------------

sigma2_ |

heteroINT | .0673591 .0516012 1.31 0.194 -.034853 .1695712

heteroCTRL | -.0614925 .0516012 -1.19 0.236 -.1637046 .0407196

|

1.delta_parallel_ | -.0041884 .0421322 -0.10 0.921 -.0876443 .0792674

1.ar | -.0000671 .0421322 -0.00 0.999 -.0835229 .0833888

_cons | .0020092 .0471053 0.04 0.966 -.0912973 .0953156

-----------------------------------------------------------------------------------

**panelFE spline** | Coefficient Std. err. t P>|t| [95% conf. interval]

------------------+----------------------------------------------------------------

sigma2_ |

heteroINT | .0658096 .0515497 1.28 0.204 -.0363005 .1679197

heteroCTRL | -.0629352 .0515497 -1.22 0.225 -.1650453 .0391749

|

1.delta_parallel_ | -.0044896 .0420902 -0.11 0.915 -.0878622 .078883

1.ar | -.0005712 .0420902 -0.01 0.989 -.0839438 .0828013

_cons | .0024405 .0470583 0.05 0.959 -.0907729 .0956539

-----------------------------------------------------------------------------------

**Panel FGLS** | Coefficient Std. err. t P>|t| [95% conf. interval]

------------------+----------------------------------------------------------------

sigma2_ |

heteroINT | .0670936 .0519705 1.29 0.199 -.0358499 .1700371

heteroCTRL | -.0614051 .0519705 -1.18 0.240 -.1643486 .0415385

|

1.delta_parallel_ | -.0043226 .0424337 -0.10 0.919 -.0883756 .0797305

1.ar | .0017544 .0424337 0.04 0.967 -.0822986 .0858074

_cons | .0012036 .0474423 0.03 0.980 -.0927706 .0951777

Diff in Diff

-----------------------------------------------------------------------------------

**Panel FE** | Coefficient Std. err. t P>|t| [95% conf. interval]

------------------+----------------------------------------------------------------

sigma2_ |

heteroINT | .0457826 .7650691 0.06 0.952 -1.469672 1.561237

heteroCTRL | 6.93997 .7650691 9.07 0.000 5.424515 8.455424

|

1.delta_parallel_ | -2.484815 .6246763 -3.98 0.000 -3.722179 -1.247452

1.ar | -.1063494 .6246763 -0.17 0.865 -1.343713 1.131014

_cons | -2.274807 .6984094 -3.26 0.001 -3.658222 -.8913927

-----------------------------------------------------------------------------------

**panelFE_Trend** | Coefficient Std. err. t P>|t| [95% conf. interval]

------------------+----------------------------------------------------------------

sigma2_ |

heteroINT | .0464236 .7686562 0.06 0.952 -1.476136 1.568984

heteroCTRL | 7.100089 .7686562 9.24 0.000 5.577529 8.622649

|

1.delta_parallel_ | -2.383595 .6276052 -3.80 0.000 -3.62676 -1.14043

1.ar | .0007081 .6276052 0.00 0.999 -1.242457 1.243873

_cons | -2.382083 .7016839 -3.39 0.001 -3.771983 -.9921818

-----------------------------------------------------------------------------------

**Panel FE spline** | Coefficient Std. err. t P>|t| [95% conf. interval]

------------------+----------------------------------------------------------------

sigma2_ |

heteroINT | .0442702 .7650844 0.06 0.954 -1.471215 1.559755

heteroCTRL | 6.939338 .7650844 9.07 0.000 5.423853 8.454823

|

1.delta_parallel_ | -2.490437 .6246888 -3.99 0.000 -3.727826 -1.253049

1.ar | -.1061635 .6246888 -0.17 0.865 -1.343552 1.131225

_cons | -2.274889 .6984233 -3.26 0.001 -3.658331 -.8914464

-----------------------------------------------------------------------------------

**Panel FGLS Trend**| Coefficient Std. err. t P>|t| [95% conf. interval]

------------------+----------------------------------------------------------------

sigma2_ |

heteroINT | .0464145 .7686637 0.06 0.952 -1.47616 1.568989

heteroCTRL | 7.100863 .7686637 9.24 0.000 5.578288 8.623437

|

1.delta_parallel_ | -2.384184 .6276113 -3.80 0.000 -3.627361 -1.141006

1.ar | .0002749 .6276113 0.00 1.000 -1.242902 1.243452

_cons | -2.381999 .7016907 -3.39 0.001 -3.771914 -.9920851

ITS of the difference

-----------------------------------------------------------------------------------

**Diff** | Coefficient Std. err. t P>|t| [95% conf. interval]

------------------+----------------------------------------------------------------

sigma2_ |

heteroINT | .0392315 1.102308 0.04 0.972 -2.168957 2.24742

heteroCTRL | 7.106822 1.102308 6.45 0.000 4.898633 9.315011

|

1.delta_parallel_ | -2.383026 .9000309 -2.65 0.011 -4.186005 -.5800476

0.ar | 0 (omitted)

_cons | -2.380614 .9000309 -2.65 0.011 -4.183593 -.5776355

-----------------------------------------------------------------------------------

**Diff X** | Coefficient Std. err. t P>|t| [95% conf. interval]

------------------+----------------------------------------------------------------

sigma2_ |

heteroINT | .0393876 .7653469 0.05 0.959 -1.476617 1.555392

heteroCTRL | 6.946811 .7653469 9.08 0.000 5.430806 8.462816

|

1.delta_parallel_ | -2.489856 .6249031 -3.98 0.000 -3.727669 -1.252043

1.ar | -.1053571 .6249031 -0.17 0.866 -1.34317 1.132456

_cons | -2.275325 .6986629 -3.26 0.001 -3.659242 -.8914079

-----------------------------------------------------------------------------------

**Diff Trend** | Coefficient Std. err. t P>|t| [95% conf. interval]

------------------+----------------------------------------------------------------

sigma2_ |

heteroINT | .061039 .0536945 1.14 0.260 -.046524 .1686021

heteroCTRL | -.063168 .0536945 -1.18 0.244 -.170731 .0443951

|

1.delta_parallel_ | -.0006414 .0438414 -0.01 0.988 -.0884662 .0871835

0.ar | 0 (omitted)

_cons | .0053963 .0438414 0.12 0.902 -.0824286 .0932211

-----------------------------------------------------------------------------------

**Diff X trend** | Coefficient Std. err. t P>|t| [95% conf. interval]

------------------+----------------------------------------------------------------

sigma2_ |

heteroINT | .064706 .0508221 1.27 0.206 -.0359627 .1653748

heteroCTRL | -.0587642 .0508221 -1.16 0.250 -.159433 .0419045

|

1.delta_parallel_ | -.0048607 .0414961 -0.12 0.907 -.0870564 .077335

1.ar | .004567 .0414961 0.11 0.913 -.0776287 .0867627

_cons | -.0001046 .046394 -0.00 0.998 -.0920022 .091793

-----------------------------------------------------------------------------------

-----------------------------------------------------------------------------------

**Diff Spline** | Coefficient Std. err. t P>|t| [95% conf. interval]

------------------+----------------------------------------------------------------

sigma2_ |

heteroINT | .061039 .0536945 1.14 0.260 -.046524 .1686021

heteroCTRL | -.063168 .0536945 -1.18 0.244 -.170731 .0443951

|

1.delta_parallel_ | -.0006414 .0438414 -0.01 0.988 -.0884662 .0871835

0.ar | 0 (omitted)

_cons | .0053963 .0438414 0.12 0.902 -.0824286 .0932211

-----------------------------------------------------------------------------------

**Diff X spline** | Coefficient Std. err. t P>|t| [95% conf. interval]

------------------+----------------------------------------------------------------

sigma2_ |

heteroINT | .063566 .0491777 1.29 0.199 -.0338456 .1609776

heteroCTRL | -.0401901 .0491777 -0.82 0.415 -.1376017 .0572215

|

1.delta_parallel_ | .0067023 .0401535 0.17 0.868 -.0728339 .0862386

1.ar | .0163883 .0401535 0.41 0.684 -.063148 .0959246

_cons | -.0117165 .0448929 -0.26 0.795 -.1006407 .0772077

Control as covariate

-----------------------------------------------------------------------------------

**Ols cov** | Coefficient Std. err. t P>|t| [95% conf. interval]

------------------+----------------------------------------------------------------

sigma2_ |

heteroINT | -1.72092 .3780546 -4.55 0.000 -2.478254 -.9635857

heteroCTRL | .9478249 .3780546 2.51 0.015 .1904906 1.705159

|

1.delta_parallel_ | -1.339823 .3086803 -4.34 0.000 -1.958184 -.7214622

0.ar | 0 (omitted)

_cons | -.7373466 .3086803 -2.39 0.020 -1.355708 -.1189857

-----------------------------------------------------------------------------------

**OLS cov Trend** | Coefficient Std. err. t P>|t| [95% conf. interval]

------------------+----------------------------------------------------------------

sigma2_ |

heteroINT | .0797908 .0389501 2.05 0.045 .0017644 .1578171

heteroCTRL | .0223921 .0389501 0.57 0.568 -.0556343 .1004185

|

1.delta_parallel_ | .0144217 .0318026 0.45 0.652 -.0492866 .07813

0.ar | 0 (omitted)

_cons | -1.007551 .0318026 -31.68 0.000 -1.071259 -.9438425

-----------------------------------------------------------------------------------

**OLS cov spline** | Coefficient Std. err. t P>|t| [95% conf. interval]

------------------+----------------------------------------------------------------

sigma2_ |

heteroINT | .0780443 .0390806 2.00 0.051 -.0002435 .1563321

heteroCTRL | .0225681 .0390806 0.58 0.566 -.0557197 .1008559

|

1.delta_parallel_ | .0145915 .0319092 0.46 0.649 -.0493302 .0785133

0.ar | 0 (omitted)

_cons | -1.007644 .0319092 -31.58 0.000 -1.071566 -.9437226

Uncontrolled ITS

-----------------------------------------------------------------------------------

**OLS** | Coefficient Std. err. t P>|t| [95% conf. interval]

------------------+----------------------------------------------------------------

sigma2_ |

heteroINT | .0221746 .5955566 0.04 0.970 -1.170869 1.215218

heteroCTRL | 3.560545 .5955566 5.98 0.000 2.367502 4.753588

|

1.delta_parallel_ | -5.01366 .4862699 -10.31 0.000 -5.987776 -4.039544

0.ar | 0 (omitted)

_cons | -2.191594 .4862699 -4.51 0.000 -3.16571 -1.217479

-----------------------------------------------------------------------------------

**OLS trend** | Coefficient Std. err. t P>|t| [95% conf. interval]

------------------+----------------------------------------------------------------

sigma2_ |

heteroINT | .0894145 .0391697 2.28 0.026 .0109482 .1678807

heteroCTRL | .0185999 .0391697 0.47 0.637 -.0598664 .0970661

|

1.delta_parallel_ | .0166535 .0319819 0.52 0.605 -.0474139 .0807209

0.ar | 0 (omitted)

_cons | -1.008697 .0319819 -31.54 0.000 -1.072764 -.9446296

-----------------------------------------------------------------------------------

**OLS spline** | Coefficient Std. err. t P>|t| [95% conf. interval]

------------------+----------------------------------------------------------------

sigma2_ |

heteroINT | .0876025 .038916 2.25 0.028 .0096445 .1655605

heteroCTRL | .0186586 .038916 0.48 0.633 -.0592994 .0966166

|

1.delta_parallel_ | .0164983 .0317747 0.52 0.606 -.0471542 .0801508

0.ar | 0 (omitted)

_cons | -1.008565 .0317747 -31.74 0.000 -1.072218 -.9449128

ARMA models for control as covariate - autoregressive scenario

-----------------------------------------------------------------------------------

**OLS MA** | Coefficient Std. err. t P>|t| [95% conf. interval]

------------------+----------------------------------------------------------------

sigma2_ |

heteroINT | -1.948795 .4140969 -4.71 0.000 -2.778331 -1.119259

heteroCTRL | .8378229 .4140969 2.02 0.048 .0082871 1.667359

|

1.delta_parallel_ | -1.93564 .3381087 -5.72 0.000 -2.612953 -1.258327

1.ar | 0 (omitted)

_cons | -.6255739 .3381087 -1.85 0.070 -1.302887 .0517392

-----------------------------------------------------------------------------------

**OLS trend MA** | Coefficient Std. err. t P>|t| [95% conf. interval]

------------------+----------------------------------------------------------------

sigma2_ |

heteroINT | .0792517 .0611896 1.30 0.201 -.0433257 .2018292

heteroCTRL | .0202781 .0611896 0.33 0.742 -.1022993 .1428556

|

1.delta_parallel_ | .0138434 .0499611 0.28 0.783 -.0862407 .1139274

1.ar | 0 (omitted)

_cons | -1.005948 .0499611 -20.13 0.000 -1.106032 -.905864

-----------------------------------------------------------------------------------

**OLS spline MA** | Coefficient Std. err. t P>|t| [95% conf. interval]

------------------+----------------------------------------------------------------

sigma2_ |

heteroINT | .0594363 .0554374 1.07 0.288 -.0516182 .1704908

heteroCTRL | .0407552 .0554374 0.74 0.465 -.0702993 .1518096

|

1.delta_parallel_ | .0219986 .0452645 0.49 0.629 -.068677 .1126742

1.ar | 0 (omitted)

_cons | -1.009916 .0452645 -22.31 0.000 -1.100592 -.9192405

-----------------------------------------------------------------------------------

ARMA models for uncontroleld models - autoregressive scenario

-----------------------------------------------------------------------------------

**OLS MA** | Coefficient Std. err. t P>|t| [95% conf. interval]

------------------+----------------------------------------------------------------

sigma2_ |

heteroINT | -.0101127 .5535566 -0.02 0.985 -1.11902 1.098795

heteroCTRL | 3.262655 .5535566 5.89 0.000 2.153747 4.371562

|

1.delta_parallel_ | -4.587902 .4519771 -10.15 0.000 -5.493321 -3.682483

1.ar | 0 (omitted)

_cons | -2.081937 .4519771 -4.61 0.000 -2.987356 -1.176518

-----------------------------------------------------------------------------------

**OLS trend MA** | Coefficient Std. err. t P>|t| [95% conf. interval]

------------------+----------------------------------------------------------------

sigma2_ |

heteroINT | .1019229 .0606291 1.68 0.098 -.0195319 .2233776

heteroCTRL | .0180755 .0606291 0.30 0.767 -.1033792 .1395302

|

1.delta_parallel_ | .0199622 .0495035 0.40 0.688 -.0792052 .1191296

1.ar | 0 (omitted)

_cons | -1.009125 .0495035 -20.38 0.000 -1.108292 -.9099574

-----------------------------------------------------------------------------------

**OLS spline MA** | Coefficient Std. err. t P>|t| [95% conf. interval]

------------------+----------------------------------------------------------------

sigma2_ |

heteroINT | .0970806 .0580538 1.67 0.100 -.0192153 .2133764

heteroCTRL | .0406887 .0580538 0.70 0.486 -.0756071 .1569846

|

1.delta_parallel_ | .0323542 .0474008 0.68 0.498 -.062601 .1273093

1.ar | 0 (omitted)

_cons | -1.01539 .0474008 -21.42 0.000 -1.110345 -.9204352

## A5. Data generation process.

1. A number of ordered observations (twice the length of the series) is created with half assigned to the treatment group ($D=1$) and half not ($D=0$). The number varied across simulations.
2. Within each group, the first half of observations are assigned to be in the pre-intervention period (Post=0) and the second half in the post-intervention (Post=1)
3. For each period in the treatment group, time-varying components of observed covariates are drawn from standard normal distributions X1 with mean_t_ 10 and standard deviation (sd_t_) 3.5, in line with mean and sd of the intervention group of our motivating example
4. A new variable series X2 correlated (ρ=0.2) with the one built in 3) is built with the following procedure: $X2=X1+\sqrt{\frac{{sd}_{t}^{2}}{\rho^{2}-1}}\cdot invnorm(uniform\left( 0,1 \right))$, where invnrom is the inverse cumulative standard normal distribution.
5. Standardisation of X1 and X2 to keep same variance and correlation across them
6. Building a time invariant variable U1 representing the difference between the intervention and control series with mean 7.3, in line with the difference between the series in the motivating example. The time invariant variable U1_t is built by two components which change in every simulation: U1=N(0,20) and S1=|N(7.3,7.3)|, U1_t=U1+S1 if D=1
7. In line with O’Neill et al. , a variable $\lambda_{t}$ is built representing the effect of unobserved components ($\mu_{i}$) that varying over time (both in pre and post intervention periods). The effect of the unobserved covariates are generated as a combination of a constant, a linear trend and a sinewave: $\lambda_{t}=\left( 1+\delta\left( 1-\frac{\left( t-T \right)}{Q} \right)+A*\sin\left( \frac{2\pi}{w} \right) \right)$. Where *A* is the amplitude, *w* is the wavelength and $\delta$ determines the slope of the linear trend while the inclusion of $\left( 1-\frac{\left( t-T \right)}{Q} \right)$ ensures that the maximum value of $\lambda_{t}$ does not change as the number of periods considered (*T*) changes. Whenever there was a parallel trend $\lambda_{t}$ was equal to 1. When the parallel trend assumption did not hold, T represent the total number of time points of the series (series length), Q was kept in being 1/3 of T, A was equal to 2, following a seasonality of the same amplitude of the effect, and w was equal to 26, hypothesising a yearly seasonality having a two-week time frame in our case study. $\delta$ was 0.55.
8. Building a variable con=Post+$\varphi$ , with $\varphi$=N(0,0.01). CON is a time invariable variable in both treatment and control series representing a constant unmeasured confound happening in the post intervention period (implying the need of a control).
9. Finally, a normally distributed idiosyncratic error, $\varepsilon_{it}$, with mean 0 and standard deviation $\sigma_{\varepsilon}$ is created. See Table 1 for different hypotheses around the error.
10. The variables created in steps (1) to (9) are then combined to generate the observed outcome variable:

$Y_{it}=X_{it}\beta+\tau\left( D\times Post \right)-con+\lambda_{t}\mu_{i}+\varepsilon_{it}$, where $\tau$ represent the 'effect' size. The process in steps (1) to (9) is repeated to create 300 datasets for each simulation scenario using the relevant parameters for that scenario. For the first scenarios, a larger sample of 1000 datasets was then simulated and checked using summary statistics. After noticing only minor differences with a simulation based on 300 datasets, we set the “burn in” period at 300 for time efficiency reasons.

## A6a. Ratio average model S.E. and empirical S.E.

A ratio close to 1 means an unbiased estimation of the variance, important to inference purposes (confidence intervals and significance tests). A ratio greater than 1 is an upward bias of the model S.E., e.g, the model enlarges the variance in the original dataset. Conversely, ratio smaller than 1 is a downward bias of the model S.E. Figures in Appendix A5 b-c, shows the models with the ratio closest to 1.

**Independent errors**

Under *parallel trends* and homoscedastic errors, most methods aligned with the DGP produced a variance ratio that converges to 1. However, models employing Driscoll-Kraay (DK) standard errors persistently underestimated the variance (ratio < 1), while specifications incorporating additional terms, such as linear time trends (but not splines), consistently overestimated it (ratio > 1). In homoscedastic, *unparallel* scenarios, DiD estimators generally underestimated the variance. Similarly, ITS of the difference tended to underestimate the variance unless spline functions are included, in which case the ratio converges to 1 as n increases.

The impact of heteroscedasticity did not vary significantly depending on whether it affects the intervention or the control series. Under heteroscedastic conditions, panel specifications utilizing DK or FGLS errors —in both CITS and DiD designs—yield variance ratios near 1. Similarly, designs incorporating splines exhibit ratios close to 1, except in cases of large sample sizes (n ≥ 184), which in this scenario corresponded to extreme shifts in variance. Single ITS models perform well under parallel trends; however, in non-parallel contexts, they required the inclusion of a linear trend to avoid underestimating the variance.

**Autocorrelated errors**

Aside from single ITS models which all incorporated ARIMA corrections for correlated errors, most methods tended to underestimate the variance. An exception was in panel models using DK standard errors which in CITS and DiD yielded a variance ratio converging to 1 as the sample size increased. GLS specifications exhibited similar asymptotic behaviour, though they had a slower rate of convergence to 1.

## A6b. Figure of ratio avgModelSE/EmpiricalSE – independent errors

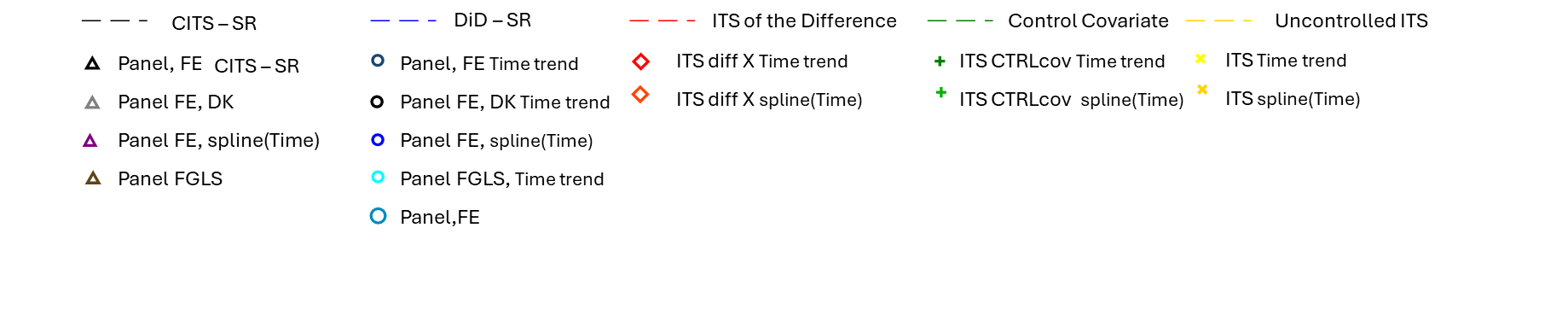


## A6c. Figure of ratio avgModelSE/EmpiricalSE – errors autocorrelated *

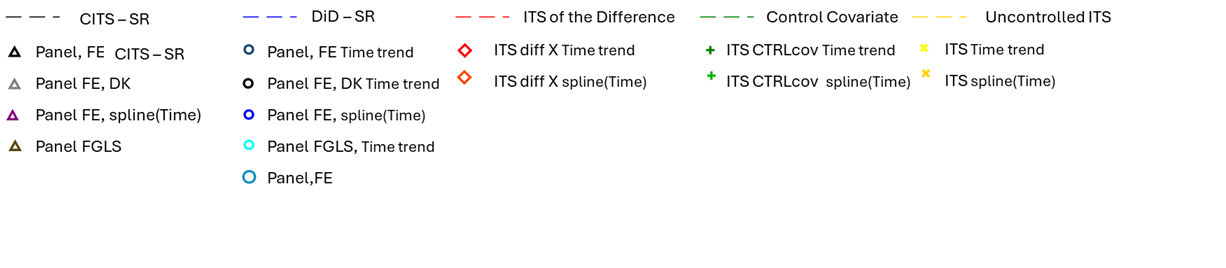


*= Control covariate and uncontrolled ITS models in this scenario include ARMA components for

## A7a. Coverage Independent error scenario

| Heteroscedastic scenario | Parallel scenario | # data points | **Controlled segmented regression (CITS)** | | | | **DiD framework segmented regression** | | | | | | **ITS of the difference** | | | | | | **control as covariate** | | | **Uncontrolled ITS** | | |
| --- | --- | --- | --- | --- | --- | --- | --- | --- | --- | --- | --- | --- | --- | --- | --- | --- | --- | --- | --- | --- | --- | --- | --- | --- |
|  |  |  | panel FE T | panelFE DK T | Panel FE splnT | panel FGLS T | panel FE | panelFE DK | panel FE T | panelFE DK T | Panel FE splnT | panel FGLS T | diff | diff X | diff T | diff X T | diff splnT | diff X splnT | Ols C | Ols C T | ols C splnT | Ols | Ols T | ols splnT |
| Homoscedastic | Parallel trend | 24 | 0.9500 | 0.8567 | 0.9433 | 0.9100 | 0.9567 | 0.9200 | **0.9600** | 0.9233 | 0.9300 | **0.9600** | 0.9433 | 0.9533 | 0.9533 | 0.9467 | 0.9500 | 0.9533 | 0.0000 | 0.0000 | 0.0000 | 0.0000 | 0.0000 | 0.0000 |
|  |  | 32 | 0.9467 | 0.8833 | 0.9500 | 0.9333 | 0.9333 | 0.8900 | 0.9333 | 0.8867 | 0.9133 | 0.9333 | 0.9467 | 0.9333 | 0.9433 | **0.9633** | 0.9500 | **0.9633** | 0.0000 | 0.0000 | 0.0000 | 0.0000 | 0.0000 | 0.0000 |
|  |  | 40 | 0.9500 | 0.8867 | 0.9500 | 0.9433 | 0.9467 | 0.8833 | 0.9467 | 0.8833 | 0.9133 | 0.9500 | 0.9600 | 0.9367 | **0.9667** | 0.9567 | **0.9667** | 0.9567 | 0.0000 | 0.0000 | 0.0000 | 0.0000 | 0.0000 | 0.0000 |
|  |  | 48 | 0.9533 | 0.9000 | 0.9533 | 0.9433 | 0.9433 | 0.9100 | 0.9433 | 0.9133 | 0.9133 | 0.9433 | 0.9300 | 0.9500 | 0.9500 | **0.9567** | 0.9533 | **0.9567** | 0.0000 | 0.0000 | 0.0000 | 0.0000 | 0.0000 | 0.0000 |
|  |  | 56 | 0.9500 | 0.9000 | 0.9500 | 0.9400 | 0.9700 | 0.9433 | 0.9700 | 0.9433 | 0.9667 | 0.9700 | 0.9600 | **0.9733** | 0.9300 | 0.9433 | 0.9333 | 0.9467 | 0.0000 | 0.0000 | 0.0000 | 0.0000 | 0.0000 | 0.0000 |
|  |  | 72 | 0.9433 | 0.9000 | 0.9433 | 0.9267 | 0.9267 | 0.9133 | 0.9233 | 0.9133 | 0.9267 | 0.9233 | **0.9700** | 0.9300 | **0.9700** | 0.9400 | **0.9700** | 0.9400 | 0.0000 | 0.0000 | 0.0000 | 0.0000 | 0.0000 | 0.0000 |
|  |  | 88 | 0.9500 | 0.9133 | 0.9500 | 0.9500 | 0.9533 | 0.9267 | 0.9533 | 0.9333 | 0.9400 | 0.9533 | **0.9667** | 0.9467 | 0.9433 | 0.9567 | 0.9467 | 0.9533 | 0.0000 | 0.0000 | 0.0000 | 0.0000 | 0.0000 | 0.0000 |
|  |  | 120 | 0.9567 | 0.9400 | 0.9567 | 0.9467 | 0.9300 | 0.9200 | 0.9300 | 0.9233 | 0.9333 | 0.9333 | 0.9367 | 0.9367 | **0.9600** | 0.9500 | **0.9600** | 0.9500 | 0.0000 | 0.0000 | 0.0000 | 0.0000 | 0.0000 | 0.0000 |
|  |  | 184 | 0.9533 | 0.9267 | 0.9533 | 0.9467 | 0.9467 | 0.9367 | 0.9467 | 0.9367 | 0.9433 | 0.9467 | **0.9700** | 0.9367 | 0.9467 | 0.9567 | 0.9467 | 0.9567 | 0.0000 | 0.0000 | 0.0000 | 0.0000 | 0.0000 | 0.0000 |
|  |  | 312 | 0.9500 | 0.9333 | 0.9500 | 0.9500 | **0.9633** | 0.9567 | **0.9633** | 0.9567 | 0.9600 | **0.9633** | 0.9600 | 0.9600 | 0.9233 | 0.9500 | 0.9233 | 0.9500 | 0.0000 | 0.0000 | 0.0000 | 0.0000 | 0.0000 | 0.0000 |
|  | Unparallel trend | 24 | 0.9500 | 0.8567 | 0.9433 | 0.9100 | 0.3900 | 0.0833 | 0.0100 | 0.0100 | 0.0100 | 0.0100 | 0.0900 | 0.0100 | **0.9533** | 0.9467 | 0.9500 | **0.9533** | 0.6300 | 0.0000 | 0.0000 | 0.0067 | 0.0000 | 0.0000 |
|  |  | 32 | 0.9467 | 0.8833 | 0.9500 | 0.9333 | 0.2967 | 0.0500 | 0.0100 | 0.0067 | 0.0067 | 0.0100 | 0.0567 | 0.0100 | 0.9433 | **0.9633** | 0.9500 | **0.9633** | 0.5833 | 0.0000 | 0.0000 | 0.0200 | 0.0000 | 0.0000 |
|  |  | 40 | 0.9500 | 0.8867 | 0.9500 | 0.9433 | 0.2667 | 0.0400 | 0.0067 | 0.0067 | 0.0067 | 0.0067 | 0.0633 | 0.0067 | **0.9667** | 0.9567 | **0.9667** | 0.9567 | 0.5533 | 0.0000 | 0.0000 | 0.0100 | 0.0000 | 0.0000 |
|  |  | 48 | 0.9533 | 0.9000 | 0.9533 | 0.9433 | 0.1967 | 0.0300 | 0.0067 | 0.0067 | 0.0067 | 0.0067 | 0.0500 | 0.0100 | 0.9500 | **0.9567** | 0.9533 | **0.9567** | 0.4800 | 0.0000 | 0.0000 | 0.0200 | 0.0000 | 0.0000 |
|  |  | 56 | **0.9500** | 0.9000 | **0.9500** | 0.9400 | 0.2233 | 0.0233 | 0.0067 | 0.0067 | 0.0067 | 0.0067 | 0.0433 | 0.0067 | 0.9300 | 0.9433 | 0.9333 | 0.9467 | 0.5033 | 0.0000 | 0.0000 | 0.0133 | 0.0000 | 0.0000 |
|  |  | 72 | 0.9433 | 0.9000 | 0.9433 | 0.9267 | 0.2033 | 0.0167 | 0.0067 | 0.0100 | 0.0100 | 0.0067 | 0.0533 | 0.0100 | **0.9700** | 0.9400 | **0.9700** | 0.9400 | 0.4933 | 0.0000 | 0.0000 | 0.0067 | 0.0000 | 0.0000 |
|  |  | 88 | 0.9500 | 0.9133 | 0.9500 | 0.9500 | 0.2367 | 0.0133 | 0.0033 | 0.0033 | 0.0033 | 0.0033 | 0.0433 | 0.0033 | 0.9433 | **0.9567** | 0.9467 | 0.9533 | 0.5067 | 0.0000 | 0.0000 | 0.0000 | 0.0000 | 0.0000 |
|  |  | 120 | 0.9567 | 0.9400 | 0.9567 | 0.9467 | 0.1467 | 0.0167 | 0.0133 | 0.0133 | 0.0133 | 0.0133 | 0.0400 | 0.0133 | **0.9600** | 0.9500 | **0.9600** | 0.9500 | 0.4267 | 0.0000 | 0.0000 | 0.0067 | 0.0000 | 0.0000 |
|  |  | 184 | 0.9533 | 0.9267 | 0.9533 | 0.9467 | 0.1300 | 0.0100 | 0.0033 | 0.0033 | 0.0033 | 0.0033 | 0.0300 | 0.0033 | 0.9467 | **0.9567** | 0.9467 | **0.9567** | 0.3633 | 0.0000 | 0.0000 | 0.0100 | 0.0000 | 0.0000 |
|  |  | 312 | **0.9500** | 0.9333 | **0.9500** | **0.9500** | 0.1100 | 0.0033 | 0.0000 | 0.0000 | 0.0000 | 0.0000 | 0.0267 | 0.0000 | 0.9233 | **0.9500** | 0.9233 | **0.9500** | 0.2833 | 0.0000 | 0.0000 | 0.0133 | 0.0000 | 0.0000 |
| Heteroscedastic (control higher variance) | Parallel trend | 24 | 0.4767 | 0.8400 | 0.9400 | 0.9067 | 0.5467 | 0.9033 | 0.5433 | 0.9033 | 0.9233 | 0.5433 | **0.9467** | **0.9467** | 0.9433 | 0.9167 | 0.9367 | 0.9400 | 0.6267 | 0.7967 | 0.8067 | 0.6467 | 0.8133 | 0.8100 |
|  |  | 32 | 0.4933 | 0.8533 | 0.9533 | 0.9367 | 0.4533 | 0.9167 | 0.4567 | 0.9200 | 0.9567 | 0.4933 | 0.9500 | **0.9667** | 0.9433 | 0.9367 | 0.9400 | 0.9533 | 0.6333 | 0.8300 | 0.8167 | 0.5900 | 0.8267 | 0.8233 |
|  |  | 40 | 0.4167 | 0.8900 | **0.9633** | 0.9400 | 0.4033 | 0.9067 | 0.4300 | 0.9067 | 0.9233 | 0.4167 | 0.9400 | 0.9267 | 0.9367 | 0.9400 | 0.9333 | 0.9600 | 0.5333 | 0.8100 | 0.8033 | 0.5300 | 0.7933 | 0.7867 |
|  |  | 48 | 0.4433 | 0.9167 | **0.9767** | 0.9633 | 0.4100 | 0.9033 | 0.4033 | 0.9033 | 0.9300 | 0.4267 | 0.9400 | 0.9367 | 0.9633 | 0.9600 | 0.9633 | **0.9767** | 0.4400 | 0.7067 | 0.7133 | 0.4200 | 0.7100 | 0.7067 |
|  |  | 56 | 0.4133 | 0.9067 | **0.9733** | 0.9467 | 0.4033 | 0.9300 | 0.3867 | 0.9333 | 0.9533 | 0.3933 | 0.9533 | 0.9667 | 0.9433 | 0.9467 | 0.9433 | 0.9633 | 0.4800 | 0.7667 | 0.7667 | 0.4833 | 0.7633 | 0.7533 |
|  |  | 72 | 0.3433 | 0.9300 | 0.9467 | 0.9300 | 0.3367 | 0.9433 | 0.3467 | 0.9433 | 0.9500 | 0.3467 | 0.9533 | 0.9567 | **0.9600** | 0.9500 | **0.9600** | 0.9433 | 0.3733 | 0.6867 | 0.6900 | 0.3500 | 0.6833 | 0.6800 |
|  |  | 88 | 0.3167 | 0.8967 | 0.9333 | 0.9300 | 0.3367 | 0.9333 | 0.3333 | 0.9333 | 0.9500 | 0.3300 | **0.9533** | **0.9533** | 0.9367 | 0.9233 | 0.9367 | 0.9500 | 0.3633 | 0.6900 | 0.6900 | 0.3600 | 0.6867 | 0.6867 |
|  |  | 120 | 0.3333 | 0.9400 | **0.9667** | 0.9600 | 0.3400 | 0.9300 | 0.3467 | 0.9300 | 0.9367 | 0.3433 | 0.9367 | 0.9367 | 0.9500 | 0.9633 | 0.9500 | 0.9567 | 0.2500 | 0.6300 | 0.6300 | 0.2400 | 0.6200 | 0.6200 |
|  |  | 184 | 0.2567 | 0.9400 | 0.9533 | 0.9467 | 0.2367 | 0.9433 | 0.2300 | 0.9433 | 0.9467 | 0.2367 | 0.9567 | 0.9533 | **0.9633** | 0.9433 | **0.9633** | 0.9567 | 0.1567 | 0.5100 | 0.5100 | 0.1433 | 0.5067 | 0.5067 |
|  |  | 312 | 0.2400 | 0.9467 | **0.9767** | 0.9633 | 0.1967 | 0.9300 | 0.1900 | 0.9300 | 0.9400 | 0.1933 | 0.9500 | 0.9367 | 0.9467 | 0.9567 | 0.9467 | 0.9667 | 0.0567 | 0.3967 | 0.4000 | 0.0633 | 0.4033 | 0.4033 |
|  | Unparallel trend | 24 | 0.4733 | 0.8467 | **0.9467** | 0.9067 | 0.2433 | 0.4700 | 0.2400 | 0.4800 | 0.4400 | 0.2200 | 0.4767 | 0.4867 | 0.9433 | 0.9167 | 0.9333 | 0.9367 | 0.2133 | 0.8700 | 0.8633 | 0.0633 | 0.8667 | 0.8733 |
|  |  | 32 | 0.5033 | 0.8567 | **0.9500** | 0.9367 | 0.1900 | 0.3533 | 0.1367 | 0.3533 | 0.3333 | 0.1533 | 0.3633 | 0.3500 | 0.9467 | 0.9300 | 0.9433 | 0.9467 | 0.1533 | 0.9133 | 0.9000 | 0.0533 | 0.9067 | 0.8967 |
|  |  | 40 | 0.3967 | 0.8967 | **0.9600** | 0.9467 | 0.1433 | 0.2533 | 0.1333 | 0.2633 | 0.2667 | 0.1367 | 0.2933 | 0.3000 | 0.9467 | 0.9433 | 0.9467 | **0.9600** | 0.1400 | 0.8633 | 0.8600 | 0.0567 | 0.8633 | 0.8600 |
|  |  | 48 | 0.4333 | 0.9133 | 0.9667 | 0.9567 | 0.1133 | 0.2300 | 0.0700 | 0.2300 | 0.2400 | 0.0700 | 0.2833 | 0.2500 | 0.9600 | 0.9633 | 0.9600 | **0.9733** | 0.1233 | 0.8500 | 0.8433 | 0.0733 | 0.8333 | 0.8300 |
|  |  | 56 | 0.4267 | 0.9133 | **0.9667** | 0.9500 | 0.1333 | 0.2733 | 0.1033 | 0.2767 | 0.2600 | 0.1167 | 0.2733 | 0.2700 | 0.9433 | 0.9500 | 0.9433 | 0.9633 | 0.0933 | 0.8733 | 0.8733 | 0.0667 | 0.8733 | 0.8767 |
|  |  | 72 | 0.3333 | 0.9267 | 0.9433 | 0.9300 | 0.0967 | 0.2633 | 0.0633 | 0.2600 | 0.2533 | 0.0667 | 0.2700 | 0.2567 | **0.9533** | **0.9533** | 0.9500 | 0.9433 | 0.0600 | 0.8333 | 0.8267 | 0.0433 | 0.8233 | 0.8167 |
|  |  | 88 | 0.3467 | 0.9000 | 0.9333 | 0.9300 | 0.0933 | 0.2467 | 0.0900 | 0.2400 | 0.2367 | 0.0800 | 0.2500 | 0.2433 | 0.9267 | 0.9267 | 0.9300 | **0.9433** | 0.0733 | 0.7800 | 0.7767 | 0.0400 | 0.7867 | 0.7800 |
|  |  | 120 | 0.3500 | 0.9400 | 0.9600 | **0.9633** | 0.0633 | 0.1933 | 0.0600 | 0.1933 | 0.1900 | 0.0567 | 0.1967 | 0.1967 | 0.9533 | **0.9633** | 0.9533 | 0.9600 | 0.0733 | 0.7700 | 0.7667 | 0.0500 | 0.7667 | 0.7667 |
|  |  | 184 | 0.2633 | 0.9433 | **0.9633** | 0.9467 | 0.0333 | 0.1767 | 0.0233 | 0.1767 | 0.1733 | 0.0233 | 0.1733 | 0.1767 | 0.9600 | 0.9433 | 0.9600 | **0.9633** | 0.0467 | 0.7067 | 0.7000 | 0.0300 | 0.6967 | 0.6967 |
|  |  | 312 | 0.2367 | 0.9567 | **0.9733** | 0.9700 | 0.0233 | 0.1167 | 0.0167 | 0.1167 | 0.1133 | 0.0167 | 0.1100 | 0.1133 | 0.9600 | 0.9667 | 0.9600 | 0.9700 | 0.0333 | 0.5867 | 0.5867 | 0.0200 | 0.5833 | 0.5833 |
| Heteroscedastic (intervention higher variance) | Parallel trend | 24 | 0.4967 | 0.8867 | 0.9500 | 0.9167 | 0.5600 | 0.9267 | 0.5500 | 0.9267 | 0.9333 | 0.5167 | **0.9567** | 0.9467 | 0.9100 | 0.9167 | 0.9033 | 0.9333 | 0.9100 | 0.8867 | 0.8900 | 0.9000 | 0.8933 | 0.9000 |
|  |  | 32 | 0.5000 | 0.8733 | 0.9533 | 0.9267 | 0.4533 | 0.9100 | 0.4533 | 0.9100 | 0.9500 | 0.4400 | **0.9733** | 0.9600 | 0.9433 | 0.9367 | 0.9533 | 0.9533 | 0.8733 | 0.9067 | 0.9100 | 0.8500 | 0.9000 | 0.9033 |
|  |  | 40 | 0.4300 | 0.8800 | **0.9667** | 0.9433 | 0.3900 | 0.8933 | 0.4067 | 0.8967 | 0.9067 | 0.4000 | 0.9300 | 0.9100 | 0.9567 | 0.9467 | 0.9533 | 0.9633 | 0.7967 | 0.9100 | 0.9100 | 0.7867 | 0.9100 | 0.9133 |
|  |  | 48 | 0.4233 | 0.9133 | **0.9667** | 0.9567 | 0.4200 | 0.9100 | 0.4267 | 0.9133 | 0.9367 | 0.4100 | 0.9433 | 0.9367 | 0.9500 | 0.9567 | 0.9500 | **0.9667** | 0.7867 | 0.9400 | 0.9333 | 0.7767 | 0.9400 | 0.9333 |
|  |  | 56 | 0.4033 | 0.9100 | 0.9633 | 0.9533 | 0.3867 | 0.9367 | 0.3933 | 0.9400 | 0.9567 | 0.4033 | 0.9433 | **0.9733** | 0.9467 | 0.9533 | 0.9500 | 0.9700 | 0.7867 | 0.8967 | 0.9033 | 0.7433 | 0.9067 | 0.9100 |
|  |  | 72 | 0.3333 | 0.9167 | 0.9400 | 0.9367 | 0.3767 | 0.9333 | 0.3667 | 0.9333 | 0.9433 | 0.3733 | **0.9500** | **0.9500** | 0.9400 | **0.9500** | 0.9400 | 0.9433 | 0.7300 | 0.8900 | 0.8933 | 0.6900 | 0.8800 | 0.8800 |
|  |  | 88 | 0.3367 | 0.9133 | 0.9467 | 0.9300 | 0.3200 | 0.9367 | 0.3200 | 0.9367 | 0.9500 | 0.3200 | 0.9433 | 0.9500 | 0.9300 | 0.9267 | 0.9300 | **0.9533** | 0.6933 | 0.8800 | 0.8800 | 0.6800 | 0.8633 | 0.8567 |
|  |  | 120 | 0.3100 | 0.9433 | 0.9567 | 0.9567 | 0.3100 | 0.9133 | 0.2967 | 0.9133 | 0.9367 | 0.2833 | **0.9633** | 0.9400 | **0.9633** | 0.9600 | **0.9633** | 0.9567 | 0.6367 | 0.8467 | 0.8500 | 0.6367 | 0.8367 | 0.8400 |
|  |  | 184 | 0.3067 | 0.9467 | **0.9567** | 0.9500 | 0.2667 | 0.9500 | 0.2733 | 0.9533 | 0.9533 | 0.2700 | 0.9533 | **0.9567** | **0.9567** | 0.9400 | **0.9567** | 0.9533 | 0.5300 | 0.8167 | 0.8133 | 0.5333 | 0.8267 | 0.8267 |
|  |  | 312 | 0.2667 | 0.9567 | **0.9733** | 0.9667 | 0.2300 | 0.9300 | 0.2333 | 0.9300 | 0.9400 | 0.2333 | 0.9467 | 0.9400 | 0.9633 | 0.9667 | 0.9633 | 0.9667 | 0.3633 | 0.7033 | 0.7033 | 0.3333 | 0.6833 | 0.6833 |
|  | Unparallel trend | 24 | 0.4833 | 0.8833 | **0.9500** | 0.9200 | 0.2700 | 0.4667 | 0.2233 | 0.4667 | 0.4267 | 0.2267 | 0.4933 | 0.4867 | 0.9167 | 0.9167 | 0.9167 | 0.9367 | 0.6133 | 0.9133 | 0.9100 | 0.1200 | 0.9100 | 0.9100 |
|  |  | 32 | 0.4933 | 0.8733 | **0.9533** | 0.9267 | 0.1900 | 0.3333 | 0.1533 | 0.3400 | 0.3367 | 0.1633 | 0.3667 | 0.3500 | 0.9433 | 0.9333 | 0.9433 | 0.9500 | 0.6033 | 0.9100 | 0.9133 | 0.1167 | 0.9167 | 0.9167 |
|  |  | 40 | 0.4233 | 0.8733 | **0.9633** | 0.9500 | 0.1467 | 0.2800 | 0.0867 | 0.2800 | 0.2767 | 0.0867 | 0.3033 | 0.3000 | 0.9467 | 0.9500 | 0.9467 | 0.9600 | 0.5200 | 0.9333 | 0.9333 | 0.0667 | 0.9367 | 0.9333 |
|  |  | 48 | 0.4200 | 0.9167 | **0.9700** | 0.9600 | 0.1000 | 0.2533 | 0.0767 | 0.2433 | 0.2333 | 0.0800 | 0.2400 | 0.2500 | 0.9633 | 0.9600 | 0.9633 | **0.9700** | 0.4567 | 0.9600 | 0.9633 | 0.0833 | 0.9600 | 0.9600 |
|  |  | 56 | 0.4100 | 0.9133 | 0.9633 | 0.9500 | 0.1167 | 0.2833 | 0.1167 | 0.2833 | 0.2633 | 0.1100 | 0.2800 | 0.2700 | 0.9533 | 0.9500 | 0.9533 | **0.9667** | 0.4733 | 0.9200 | 0.9167 | 0.0667 | 0.9233 | 0.9267 |
|  |  | 72 | 0.3500 | 0.9233 | 0.9400 | 0.9367 | 0.1067 | 0.2567 | 0.0700 | 0.2467 | 0.2433 | 0.0767 | 0.2600 | 0.2567 | 0.9400 | **0.9500** | 0.9400 | 0.9433 | 0.3633 | 0.9233 | 0.9200 | 0.0300 | 0.9200 | 0.9133 |
|  |  | 88 | 0.3467 | 0.9133 | 0.9467 | 0.9267 | 0.0967 | 0.2433 | 0.0833 | 0.2433 | 0.2400 | 0.0900 | 0.2400 | 0.2467 | 0.9233 | 0.9333 | 0.9267 | **0.9500** | 0.3600 | 0.8900 | 0.8933 | 0.0333 | 0.9000 | 0.8967 |
|  |  | 120 | 0.3067 | 0.9367 | 0.9600 | 0.9600 | 0.0767 | 0.1900 | 0.0433 | 0.1833 | 0.1867 | 0.0500 | 0.1833 | 0.1933 | **0.9633** | **0.9633** | **0.9633** | 0.9600 | 0.2600 | 0.8967 | 0.9033 | 0.0367 | 0.9033 | 0.9033 |
|  |  | 184 | 0.2900 | 0.9433 | 0.9500 | 0.9500 | 0.0367 | 0.1767 | 0.0233 | 0.1767 | 0.1733 | 0.0267 | 0.1700 | 0.1800 | 0.9500 | 0.9433 | 0.9500 | **0.9567** | 0.2033 | 0.8900 | 0.8900 | 0.0333 | 0.8900 | 0.8900 |
|  |  | 312 | 0.2600 | 0.9600 | **0.9733** | 0.9667 | 0.0133 | 0.1167 | 0.0200 | 0.1167 | 0.1133 | 0.0200 | 0.1133 | 0.1133 | 0.9667 | 0.9633 | 0.9667 | 0.9700 | 0.1533 | 0.8200 | 0.8200 | 0.0300 | 0.8267 | 0.8233 |

Colours rank the performance relative to each sample size, from green (Coverage closest to 1 -optimal situation) to red (coverage further from 0).

## A7b. Coverage autocorrelated error scenario *

| Heteroscedastic scenario | Parallel scenario | # data points | **Controlled segmented regression (CITS)** | | | | **DiD framework segmented regression** | | | | | | **ITS of the difference** | | | **control as covariate*** | | | **Uncontrolled ITS*** | | |
| --- | --- | --- | --- | --- | --- | --- | --- | --- | --- | --- | --- | --- | --- | --- | --- | --- | --- | --- | --- | --- | --- |
|  |  |  | panel FE T | panelFE DK T | Panel FE splnT | panel FGLS T | panel FE | panelFE DK | panel FE T | panelFE DK T | Panel FE splnT | panel FGLS T | diff X | diff X T | diff X splnT | Ols C | Ols C T | ols C splnT | Ols | Ols T | ols splnT |
| Homoscedastic | Parallel trend | 24 | 0.8267 | 0.8100 | 0.8167 | 0.7733 | 0.8633 | 0.8700 | 0.8600 | **0.8733** | 0.8033 | 0.8567 | 0.8533 | 0.8333 | 0.8133 | 0.0000 | 0.0000 | 0.0000 | 0.0000 | 0.0000 | 0.0000 |
|  |  | 32 | 0.8600 | 0.8133 | 0.8567 | 0.8300 | 0.7900 | 0.8633 | 0.7867 | 0.8633 | 0.7733 | 0.7833 | 0.7967 | **0.8700** | 0.8667 | 0.0000 | 0.0000 | 0.0000 | 0.0000 | 0.0000 | 0.0000 |
|  |  | 40 | **0.8833** | 0.8633 | **0.8833** | 0.8500 | 0.8233 | 0.8600 | 0.8267 | 0.8633 | 0.7867 | 0.8267 | 0.8333 | 0.8667 | 0.8633 | 0.0000 | 0.0000 | 0.0000 | 0.0000 | 0.0000 | 0.0000 |
|  |  | 48 | 0.8667 | 0.8667 | 0.8667 | 0.8567 | 0.8267 | **0.8833** | 0.8100 | 0.8633 | 0.8000 | 0.8300 | 0.8367 | 0.8667 | **0.8833** | 0.0000 | 0.0000 | 0.0000 | 0.0000 | 0.0000 | 0.0000 |
|  |  | 56 | 0.8733 | 0.8867 | 0.8700 | 0.8567 | 0.8533 | 0.9100 | 0.8533 | **0.9133** | 0.8500 | 0.8500 | 0.8567 | 0.8767 | 0.8700 | 0.0000 | 0.0000 | 0.0000 | 0.0000 | 0.0000 | 0.0000 |
|  |  | 72 | 0.8533 | 0.8833 | 0.8500 | 0.8167 | 0.8033 | 0.8933 | 0.8033 | **0.8967** | 0.7933 | 0.8033 | 0.8167 | 0.8400 | 0.8367 | 0.0000 | 0.0000 | 0.0000 | 0.0000 | 0.0000 | 0.0000 |
|  |  | 88 | 0.8367 | 0.8900 | 0.8367 | 0.8133 | 0.8100 | 0.8967 | 0.8133 | **0.9000** | 0.7967 | 0.8133 | 0.8000 | 0.8333 | 0.8333 | 0.0000 | 0.0000 | 0.0000 | 0.0000 | 0.0000 | 0.0000 |
|  |  | 120 | 0.8700 | **0.9300** | 0.8733 | 0.8700 | 0.8100 | 0.9000 | 0.8033 | 0.8967 | 0.8033 | 0.8100 | 0.8233 | 0.8667 | 0.8667 | 0.0000 | 0.0000 | 0.0000 | 0.0000 | 0.0000 | 0.0000 |
|  |  | 184 | 0.8367 | 0.9133 | 0.8333 | 0.8300 | 0.8300 | **0.9200** | 0.8300 | 0.9167 | 0.8333 | 0.8300 | 0.8400 | 0.8333 | 0.8333 | 0.0000 | 0.0000 | 0.0000 | 0.0000 | 0.0000 | 0.0000 |
|  |  | 312 | 0.8300 | 0.9100 | 0.8300 | 0.8167 | 0.8600 | **0.9500** | 0.8600 | **0.9500** | 0.8500 | 0.8600 | 0.8500 | 0.8267 | 0.8267 | 0.0000 | 0.0000 | 0.0000 | 0.0000 | 0.0000 | 0.0000 |
|  | Unparallel trend | 24 | **0.8267** | 0.8100 | 0.8167 | 0.7733 | 0.3933 | 0.0767 | 0.0033 | 0.0133 | 0.0067 | 0.0067 | 0.0100 | 0.8167 | 0.8133 | 0.5367 | 0.0033 | 0.0000 | 0.0067 | 0.0033 | 0.0000 |
|  |  | 32 | 0.8600 | 0.8133 | 0.8567 | 0.8300 | 0.2967 | 0.0500 | 0.0033 | 0.0133 | 0.0133 | 0.0133 | 0.0133 | **0.8700** | 0.8667 | 0.5600 | 0.0100 | 0.0000 | 0.0200 | 0.0033 | 0.0000 |
|  |  | 40 | **0.8833** | 0.8633 | **0.8833** | 0.8500 | 0.2700 | 0.0400 | 0.0000 | 0.0067 | 0.0067 | 0.0067 | 0.0067 | 0.8733 | 0.8633 | 0.5267 | 0.0000 | 0.0000 | 0.0133 | 0.0000 | 0.0000 |
|  |  | 48 | 0.8667 | 0.8667 | 0.8667 | 0.8567 | 0.1900 | 0.0300 | 0.0033 | 0.0100 | 0.0100 | 0.0100 | 0.0100 | 0.8567 | **0.8833** | 0.4600 | 0.0033 | 0.0000 | 0.0233 | 0.0000 | 0.0000 |
|  |  | 56 | 0.8733 | **0.8867** | 0.8700 | 0.8567 | 0.2267 | 0.0200 | 0.0033 | 0.0067 | 0.0067 | 0.0067 | 0.0067 | 0.8767 | 0.8700 | 0.4967 | 0.0033 | 0.0000 | 0.0133 | 0.0000 | 0.0000 |
|  |  | 72 | 0.8533 | **0.8833** | 0.8500 | 0.8167 | 0.2033 | 0.0200 | 0.0000 | 0.0100 | 0.0100 | 0.0100 | 0.0100 | 0.8267 | 0.8367 | 0.5133 | 0.0000 | 0.0000 | 0.0067 | 0.0000 | 0.0000 |
|  |  | 88 | 0.8367 | **0.8900** | 0.8367 | 0.8133 | 0.2367 | 0.0133 | 0.0000 | 0.0033 | 0.0033 | 0.0033 | 0.0033 | 0.8300 | 0.8333 | 0.4833 | 0.0000 | 0.0000 | 0.0033 | 0.0000 | 0.0000 |
|  |  | 120 | 0.8700 | **0.9300** | 0.8733 | 0.8700 | 0.1467 | 0.0200 | 0.0067 | 0.0133 | 0.0133 | 0.0133 | 0.0133 | 0.8667 | 0.8667 | 0.4300 | 0.0000 | 0.0000 | 0.0100 | 0.0000 | 0.0000 |
|  |  | 184 | 0.8367 | **0.9133** | 0.8333 | 0.8300 | 0.1267 | 0.0133 | 0.0000 | 0.0033 | 0.0033 | 0.0033 | 0.0033 | 0.8233 | 0.8333 | 0.3567 | 0.0000 | 0.0000 | 0.0100 | 0.0000 | 0.0000 |
|  |  | 312 | 0.8300 | **0.9100** | 0.8300 | 0.8167 | 0.1100 | 0.0033 | 0.0000 | 0.0000 | 0.0000 | 0.0000 | 0.0000 | 0.8233 | 0.8267 | 0.2667 | 0.0000 | 0.0000 | 0.0133 | 0.0000 | 0.0000 |
| Heteroscedastic (control higher variance) | Parallel trend | 24 | 0.8533 | 0.8233 | 0.8533 | 0.8033 | 0.4600 | **0.9133** | 0.4833 | **0.9133** | 0.8400 | 0.4833 | 0.8900 | 0.8500 | 0.8333 | 0.6300 | 0.6533 | 0.6400 | 0.6733 | 0.6700 | 0.6567 |
|  |  | 32 | 0.8400 | 0.8033 | 0.8367 | 0.7933 | 0.3700 | **0.8700** | 0.3833 | **0.8700** | 0.8133 | 0.3900 | 0.8500 | 0.8433 | 0.8467 | 0.6833 | 0.7300 | 0.6900 | 0.6767 | 0.7267 | 0.7000 |
|  |  | 40 | 0.8767 | 0.8667 | 0.8733 | 0.8467 | 0.3400 | 0.8767 | 0.3433 | **0.8800** | 0.7667 | 0.3433 | 0.8100 | 0.8700 | 0.8733 | 0.6733 | 0.7967 | 0.7867 | 0.6533 | 0.7767 | 0.7733 |
|  |  | 48 | 0.8667 | **0.8800** | 0.8600 | 0.8333 | 0.3567 | 0.8767 | 0.3667 | 0.8767 | 0.7933 | 0.3567 | 0.8300 | 0.8667 | 0.8733 | 0.6067 | 0.7433 | 0.7267 | 0.6167 | 0.7400 | 0.7433 |
|  |  | 56 | 0.8467 | 0.8967 | 0.8433 | 0.8367 | 0.2600 | **0.9200** | 0.2633 | **0.9200** | 0.8533 | 0.2500 | 0.8600 | 0.8533 | 0.8600 | 0.6767 | 0.7933 | 0.7867 | 0.6700 | 0.7900 | 0.7733 |
|  |  | 72 | 0.8767 | 0.8967 | 0.8767 | 0.8633 | 0.3067 | **0.9133** | 0.2900 | **0.9133** | 0.8200 | 0.2967 | 0.8333 | 0.8800 | 0.8733 | 0.5700 | 0.8100 | 0.7967 | 0.5667 | 0.7900 | 0.7767 |
|  |  | 88 | 0.8433 | 0.8700 | 0.8433 | 0.8400 | 0.2200 | **0.9100** | 0.2300 | **0.9100** | 0.8433 | 0.2300 | 0.8567 | 0.8233 | 0.8467 | 0.5767 | 0.7500 | 0.7433 | 0.5833 | 0.7500 | 0.7433 |
|  |  | 120 | 0.8833 | **0.9333** | 0.8800 | 0.8700 | 0.2333 | 0.9100 | 0.2267 | 0.9100 | 0.8167 | 0.2400 | 0.8233 | 0.8733 | 0.8700 | 0.5200 | 0.7567 | 0.7500 | 0.5033 | 0.7433 | 0.7433 |
|  |  | 184 | 0.8800 | **0.9400** | 0.8733 | 0.8667 | 0.1633 | 0.9233 | 0.1533 | 0.9233 | 0.8300 | 0.1567 | 0.8333 | 0.8600 | 0.8700 | 0.4100 | 0.7033 | 0.7067 | 0.4067 | 0.7033 | 0.7033 |
|  |  | 312 | 0.8767 | **0.9400** | 0.8767 | 0.8767 | 0.1767 | 0.9267 | 0.1800 | 0.9267 | 0.8367 | 0.1733 | 0.8400 | 0.8733 | 0.8767 | 0.2600 | 0.6133 | 0.6100 | 0.2733 | 0.6167 | 0.6167 |
|  | Unparallel trend | 24 | 0.8600 | 0.8300 | 0.8533 | 0.8033 | 0.1767 | **0.9267** | 0.2400 | 0.5867 | 0.4667 | 0.1733 | 0.8400 | 0.8500 | 0.8400 | 0.2600 | 0.7233 | 0.6100 | 0.2733 | 0.7233 | 0.6167 |
|  |  | 32 | 0.8400 | 0.8033 | 0.8333 | 0.7900 | 0.2133 | 0.4433 | 0.1867 | 0.4433 | 0.3700 | 0.1867 | 0.3933 | 0.8433 | **0.8533** | 0.2400 | 0.7700 | 0.7167 | 0.1367 | 0.7800 | 0.7233 |
|  |  | 40 | **0.8833** | 0.8700 | 0.8767 | 0.8533 | 0.1233 | 0.4167 | 0.1267 | 0.4233 | 0.3000 | 0.1333 | 0.3467 | 0.8767 | 0.8700 | 0.2133 | 0.8167 | 0.8267 | 0.1200 | 0.8267 | 0.8233 |
|  |  | 48 | 0.8667 | **0.8833** | 0.8600 | 0.8433 | 0.1333 | 0.3800 | 0.1033 | 0.3833 | 0.2733 | 0.1067 | 0.2933 | 0.8733 | 0.8767 | 0.1667 | 0.8100 | 0.8067 | 0.1200 | 0.8267 | 0.8200 |
|  |  | 56 | 0.8533 | **0.8933** | 0.8533 | 0.8467 | 0.1367 | 0.3733 | 0.1200 | 0.3733 | 0.3000 | 0.1267 | 0.3067 | 0.8567 | 0.8667 | 0.1367 | 0.8433 | 0.8300 | 0.0933 | 0.8367 | 0.8400 |
|  |  | 72 | 0.8700 | **0.8933** | 0.8667 | 0.8533 | 0.1133 | 0.3967 | 0.1033 | 0.3933 | 0.3167 | 0.1000 | 0.3200 | 0.8767 | 0.8733 | 0.1000 | 0.8433 | 0.8400 | 0.0733 | 0.8533 | 0.8433 |
|  |  | 88 | 0.8533 | **0.8733** | 0.8500 | 0.8333 | 0.1133 | 0.3333 | 0.1000 | 0.3400 | 0.2667 | 0.1000 | 0.2767 | 0.8333 | 0.8500 | 0.0867 | 0.8200 | 0.8167 | 0.0633 | 0.8267 | 0.8233 |
|  |  | 120 | 0.8833 | **0.9300** | 0.8833 | 0.8700 | 0.0767 | 0.2867 | 0.0667 | 0.2867 | 0.2367 | 0.0700 | 0.2400 | 0.8767 | 0.8733 | 0.0900 | 0.8267 | 0.8267 | 0.0733 | 0.8367 | 0.8333 |
|  |  | 184 | 0.8767 | **0.9400** | 0.8733 | 0.8633 | 0.0367 | 0.2300 | 0.0200 | 0.2300 | 0.1900 | 0.0233 | 0.1933 | 0.8667 | 0.8700 | 0.0667 | 0.8067 | 0.8100 | 0.0533 | 0.8067 | 0.8100 |
|  |  | 312 | 0.8833 | **0.9367** | 0.8833 | 0.8733 | 0.0267 | 0.1967 | 0.0200 | 0.1967 | 0.1533 | 0.0200 | 0.1500 | 0.8900 | 0.8800 | 0.0533 | 0.7900 | 0.7900 | 0.0433 | 0.7800 | 0.7800 |
| Heteroscedastic (intervention higher variance) | Parallel trend | 24 | 0.8400 | 0.8333 | 0.8333 | 0.8000 | 0.4767 | 0.8933 | 0.4667 | **0.8967** | 0.8200 | 0.4367 | 0.8700 | 0.8433 | 0.8333 | 0.8333 | 0.7767 | 0.7433 | 0.8433 | 0.7900 | 0.7633 |
|  |  | 32 | 0.8500 | 0.8300 | 0.8433 | 0.8100 | 0.3633 | 0.8833 | 0.3700 | **0.8867** | 0.8200 | 0.3800 | 0.8600 | 0.8400 | 0.8500 | 0.8467 | 0.8133 | 0.7933 | 0.8267 | 0.8300 | 0.8000 |
|  |  | 40 | **0.8867** | 0.8667 | 0.8800 | 0.8533 | 0.2967 | 0.8567 | 0.3233 | 0.8600 | 0.7833 | 0.3167 | 0.8067 | 0.8633 | 0.8667 | 0.8133 | 0.8367 | 0.8333 | 0.8500 | 0.8433 | 0.8433 |
|  |  | 48 | 0.8567 | 0.8800 | 0.8567 | 0.8400 | 0.3400 | 0.8633 | 0.3333 | 0.8700 | 0.8033 | 0.3567 | 0.8300 | 0.8700 | 0.8767 | 0.8600 | 0.8800 | 0.8833 | 0.8600 | 0.8900 | **0.8967** |
|  |  | 56 | 0.8733 | 0.8933 | 0.8700 | 0.8633 | 0.3433 | **0.9100** | 0.3500 | **0.9100** | 0.8467 | 0.3300 | 0.8567 | 0.8567 | 0.8667 | 0.8633 | 0.8567 | 0.8533 | 0.8600 | 0.8700 | 0.8567 |
|  |  | 72 | 0.8700 | 0.9000 | 0.8700 | 0.8633 | 0.3167 | **0.9167** | 0.2933 | **0.9167** | 0.8167 | 0.3000 | 0.8333 | 0.8667 | 0.8667 | 0.8600 | 0.8800 | 0.8767 | 0.8433 | 0.8767 | 0.8767 |
|  |  | 88 | 0.8433 | 0.8667 | 0.8433 | 0.8333 | 0.2200 | **0.9200** | 0.2033 | **0.9200** | 0.8300 | 0.2000 | 0.8567 | 0.8200 | 0.8533 | 0.8400 | 0.8833 | 0.8800 | 0.8267 | 0.8867 | 0.8800 |
|  |  | 120 | 0.8800 | **0.9233** | 0.8800 | 0.8733 | 0.2400 | 0.9000 | 0.2433 | 0.9033 | 0.8133 | 0.2400 | 0.8200 | 0.8767 | 0.8767 | 0.8000 | 0.9033 | 0.8967 | 0.7967 | 0.8933 | 0.8833 |
|  |  | 184 | 0.8800 | **0.9333** | 0.8767 | 0.8700 | 0.1900 | **0.9333** | 0.1900 | **0.9333** | 0.8400 | 0.1867 | 0.8367 | 0.8733 | 0.8800 | 0.7367 | 0.8900 | 0.8900 | 0.7367 | 0.8867 | 0.8867 |
|  |  | 312 | 0.8833 | **0.9467** | 0.8833 | 0.8767 | 0.1567 | 0.9267 | 0.1600 | 0.9267 | 0.8467 | 0.1600 | 0.8433 | 0.8733 | 0.8833 | 0.6133 | 0.8467 | 0.8433 | 0.6133 | 0.8467 | 0.8400 |
|  | Unparallel trend | 24 | **0.8467** | **0.8467** | **0.8467** | 0.8000 | 0.2967 | 0.6000 | 0.2467 | 0.5967 | 0.4733 | 0.2167 | 0.5000 | **0.8467** | 0.8400 | 0.6300 | 0.7933 | 0.7567 | 0.3267 | 0.8133 | 0.7800 |
|  |  | 32 | 0.8433 | 0.8300 | 0.8400 | 0.8033 | 0.2100 | 0.4500 | 0.1833 | 0.4500 | 0.3633 | 0.1900 | 0.3833 | 0.8433 | **0.8500** | 0.5633 | 0.8300 | 0.8067 | 0.2433 | 0.8367 | 0.8100 |
|  |  | 40 | **0.8833** | 0.8700 | **0.8833** | 0.8667 | 0.1567 | 0.4200 | 0.1200 | 0.4167 | 0.3033 | 0.1333 | 0.3500 | 0.8667 | 0.8733 | 0.5033 | 0.8233 | 0.8167 | 0.1867 | 0.8400 | 0.8433 |
|  |  | 48 | 0.8633 | **0.8833** | 0.8633 | 0.8500 | 0.1267 | 0.3733 | 0.0933 | 0.3767 | 0.2833 | 0.0933 | 0.2900 | 0.8667 | **0.8833** | 0.4133 | 0.8700 | 0.8667 | 0.1633 | 0.8800 | 0.8733 |
|  |  | 56 | 0.8733 | **0.8933** | 0.8800 | 0.8567 | 0.1133 | 0.3800 | 0.1133 | 0.3767 | 0.3033 | 0.1167 | 0.3100 | 0.8567 | 0.8667 | 0.4333 | 0.8400 | 0.8367 | 0.1333 | 0.8667 | 0.8733 |
|  |  | 72 | 0.8667 | **0.9000** | 0.8633 | 0.8600 | 0.1067 | 0.3867 | 0.0867 | 0.3933 | 0.3033 | 0.0967 | 0.3200 | 0.8700 | 0.8667 | 0.3867 | 0.8933 | 0.8900 | 0.0967 | 0.8800 | 0.8867 |
|  |  | 88 | 0.8467 | 0.8800 | 0.8467 | 0.8367 | 0.1167 | 0.3467 | 0.0967 | 0.3467 | 0.2667 | 0.1000 | 0.2733 | 0.8267 | 0.8400 | 0.3300 | 0.8900 | 0.8967 | 0.0800 | **0.9000** | 0.8933 |
|  |  | 120 | 0.8733 | **0.9233** | 0.8733 | 0.8733 | 0.0767 | 0.2867 | 0.0633 | 0.2833 | 0.2367 | 0.0633 | 0.2400 | 0.8767 | 0.8800 | 0.2100 | 0.9200 | 0.9200 | 0.0567 | 0.9133 | 0.9167 |
|  |  | 184 | 0.8767 | **0.9367** | 0.8767 | 0.8733 | 0.0433 | 0.2367 | 0.0367 | 0.2333 | 0.1900 | 0.0433 | 0.1900 | 0.8700 | 0.8767 | 0.1333 | 0.9133 | 0.9133 | 0.0433 | 0.9200 | 0.9200 |
|  |  | 312 | 0.8767 | **0.9533** | 0.8767 | 0.8667 | 0.0133 | 0.1867 | 0.0167 | 0.1867 | 0.1533 | 0.0200 | 0.1500 | 0.8767 | 0.8833 | 0.1100 | 0.9067 | 0.9033 | 0.0433 | 0.9067 | 0.9067 |

*= Control covariate and uncontrolled ITS models in this scenario include ARMA components for autocorrelated error structure

Colours rank the performance relative to each sample size, from green (Coverage closest to 1 -optimal situation) to red (coverage further from 0).

## A7c. Figure of Coverage for error autocorrelated scenario*

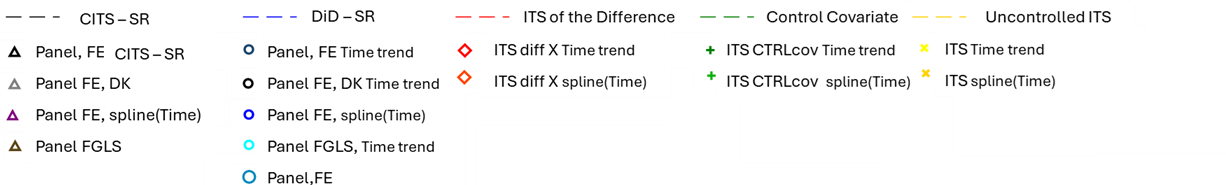


*= Control covariate and uncontrolled ITS models in this scenario include ARMA components for autocorrelated error structure

## A8. Unparallel trend detection.

| **error** | **parallel trend in DGP** | **series length**** | **Homoscedastic** | **Heteroscedastic CTRL** | **Heteroscedastic INT** |
| --- | --- | --- | --- | --- | --- |
| no error autocorrelation | Parallel | 24 | 8.00% | 9.33% | 9.33% |
|  |  | 312 | 8.33% | 7.00% | 4.00% |
|  | Unparallel | 24 | 80.00% | 23.67% | 27.00% |
|  |  | 312 | 95.00% | 65.33% | 68.33% |
| error autocorrelation | Parallel | 24 | 6.67% | 21.00% | 18.33% |
|  |  | 312 | 8.00% | 13.67% | 13.00% |
|  | Unparallel | 24 | 80.33% | 34.00% | 32.67% |
|  |  | 312 | 95.00% | 60.00% | 62.33% |
| **=the test is only on the pre-intervention periods, which in our DGP equals to half of the length of the series | | | | | |

## A9a. DGP with alternative unparallel trend

$$Y_{it}=X_{it}\beta+\tau\left( D\times Post \right)+con+2conf+\lambda_{t}\mu_{i}+\varepsilon_{it}$$

2conf is a variable being +1 for the first 1/3 of the data points, an increasing trend with unitary increase in every data point in the second third of the data points and -1 for the last 1/3 of the data points

Figure A9. Representation of intervention (red) and control (blue) series in the alternative unparallel scenario; the variable 2conf was -1 in the first 104 data points, it had a unitary increase in correspondence of every data point from the 105^th^ to the 208^th^ data points and was -1 after 208^th^. Intervention was at the 156^th^ data point.

## A9b. Results on different unparallel trend (independent error scenario) – sensitivity analysis

**Bias**

|  |  | **Controlled segmented regression (CITS)** | | | **DiD framework segmented regression** | | | | | **ITS of the difference** | | | | | | | **control as covariate** | | | | **uncontrolled** | | |  |
| --- | --- | --- | --- | --- | --- | --- | --- | --- | --- | --- | --- | --- | --- | --- | --- | --- | --- | --- | --- | --- | --- | --- | --- | --- |
| Parallel scenario | # data points | Panel FE/DK* T | Panel FE splnT | Panel FGLS T | panel FE/DK* | panel FE/DK* T | panel FGLS T | Panel FE splnT | diff | | diff T | diff X | diff X T | diff splnT | diff X splnT | Ols C | | Ols C T | ols C splnT | ols | | ols T | ols splnT | |
| UnParallel trend | 24 | 1.5712 | 1.2283 | 1.5703 | 6.1665 | 6.1786 | 6.1732 | 6.1808 | 6.1526 | | 1.1664 | 6.1891 | 1.2397 | 2.2263 | 2.2582 | 2.9854 | | 0.9946 | 0.9860 | 8.1801 | | 0.9954 | 0.9880 | |
|  | 32 | 1.4977 | 1.9625 | 1.4971 | 7.5772 | 7.5705 | 7.5747 | 7.5676 | 7.5893 | | 1.9763 | 7.5790 | 1.9629 | 0.0722 | 0.0116 | 3.8455 | | 1.0027 | 1.0013 | 8.3408 | | 1.0032 | 1.0002 | |
|  | 40 | 1.4831 | 0.9484 | 1.4846 | 7.3356 | 7.3475 | 7.3498 | 7.3477 | 7.3059 | | 0.8897 | 7.3258 | 0.9477 | 0.9400 | 0.8526 | 3.1588 | | 1.0061 | 0.9988 | 8.6301 | | 1.0062 | 0.9979 | |
|  | 48 | 1.6260 | 1.3030 | 1.6276 | 7.5005 | 7.4533 | 7.4560 | 7.4536 | 7.5325 | | 1.3419 | 7.4976 | 1.3013 | 0.3833 | 0.3746 | 3.7218 | | 0.9963 | 0.9954 | 9.1044 | | 0.9972 | 0.9944 | |
|  | 56 | 1.3971 | 2.8529 | 1.3952 | 8.5355 | 8.2379 | 8.2292 | 8.2312 | 8.5482 | | 2.8687 | 8.5433 | 2.8649 | 2.0124 | 1.9182 | 5.9324 | | 0.9980 | 0.9946 | 6.1678 | | 0.9985 | 0.9931 | |
|  | 72 | 2.8626 | 1.3964 | 2.8369 | 7.4854 | 7.3840 | 7.3864 | 7.3848 | 7.4831 | | 1.3731 | 7.4805 | 1.3972 | 0.1681 | 0.2644 | 3.4698 | | 1.0023 | 1.0023 | 8.6273 | | 1.0022 | 1.0011 | |
|  | 88 | 1.3050 | 1.6266 | 1.3054 | 7.5892 | 7.4860 | 7.4856 | 7.4866 | 7.6146 | | 1.6694 | 7.6017 | 1.6240 | 0.1908 | 0.1131 | 3.6899 | | 0.9989 | 1.0069 | 8.9866 | | 0.9987 | 1.0061 | |
|  | 120 | 0.9476 | 1.4834 | 0.9455 | 7.8798 | 7.7888 | 7.7900 | 7.7886 | 7.8988 | | 1.5268 | 7.8809 | 1.4841 | 0.1250 | 0.1486 | 3.8745 | | 1.0031 | 1.0055 | 9.7190 | | 1.0031 | 1.0052 | |
|  | 184 | 1.9718 | 1.4971 | 1.9673 | 7.6141 | 7.5812 | 7.5811 | 7.5810 | 7.6142 | | 1.4754 | 7.6205 | 1.4978 | 0.0644 | 0.1501 | 3.5327 | | 1.0002 | 0.9994 | 7.1826 | | 1.0003 | 0.9994 | |
|  | 312 | 1.2248 | 1.5710 | 1.2259 | 7.6295 | 7.4000 | 7.3997 | 7.4002 | 7.6147 | | 1.5198 | 7.6310 | 1.5721 | 0.0525 | 0.0117 | 4.0346 | | 0.9988 | 0.9947 | 9.0329 | | 0.9989 | 0.9948 | |

Colours rank the performance relative to each sample size, from green (bias closest to 0 -optimal situation) to red (bias furthest from 0).

**Ratio avgModelSE/EmpSE***

|  |  | | **Controlled segmented regression (CITS)** | | | | | **DiD framework segmented regression** | | | | **ITS of the difference** | | | | | | **control as covariate**  **uncontrolled** | | | **uncontrolled** | | |
| --- | --- | --- | --- | --- | --- | --- | --- | --- | --- | --- | --- | --- | --- | --- | --- | --- | --- | --- | --- | --- | --- | --- | --- |
| Parallel scenario | | # data points | | Panel FE T | Panel FE,DK T | panel FGLS T | Panel FE splnT | Panel FE T | Panel FE,DK T | panel FGLS T | Panel FE splnT | diff | diff | diff T | diff X T | diff splnT | diff X splnT | ols | Ols C T | ols C splnT | ols | Ols C T | ols C splnT |
| UnParallel trend | | 24 | | 0.2722 | 20.0517 | 6.8006 | 5.0785 | 0.1783 | 0.3266 | 0.2046 | 0.1689 | 0.2405 | 0.2213 | 1.4877 | 4.2006 | 1.1152 | 3.1458 | 0.4706 | 0.9850 | 1.0341 | 0.1382 | 0.9807 | 1.0091 |
|  |  | 32 | | 0.3215 | 18.0865 | 6.8486 | 5.9095 | 0.1493 | 0.3228 | 0.1768 | 0.1488 | 0.2127 | 0.1944 | 1.4666 | 5.0368 | 1.1092 | 3.9229 | 0.3460 | 1.0075 | 0.9725 | 0.1166 | 1.0149 | 0.9981 |
|  |  | 40 | | 0.3783 | 17.3620 | 7.0139 | 5.0947 | 0.1283 | 0.3067 | 0.1616 | 0.1297 | 0.1882 | 0.1727 | 1.3694 | 4.8483 | 1.3089 | 3.7678 | 0.3200 | 1.0336 | 0.9932 | 0.1033 | 1.0304 | 1.0153 |
|  |  | 48 | | 0.4954 | 13.9086 | 6.9534 | 5.2624 | 0.1167 | 0.2825 | 0.1472 | 0.1184 | 0.1743 | 0.1575 | 1.2708 | 4.6505 | 1.1358 | 4.2381 | 0.2774 | 1.0832 | 1.0502 | 0.0964 | 1.0743 | 1.0438 |
|  |  | 56 | | 0.4822 | 13.9995 | 6.6242 | 10.5687 | 0.4604 | 0.8363 | 0.4555 | 0.3066 | 0.3493 | 0.3454 | 6.3563 | 6.5731 | 1.5450 | 3.1066 | 0.0921 | 1.0385 | 3.1260 | 0.0616 | 1.0401 | 3.1160 |
|  |  | 72 | | 1.0129 | 25.1635 | 57.2458 | 5.1337 | 0.0897 | 0.2245 | 0.1151 | 0.0900 | 0.1355 | 0.1215 | 1.3398 | 5.3260 | 1.1096 | 4.0082 | 0.2632 | 1.0495 | 1.0021 | 0.0759 | 1.0304 | 1.0067 |
|  |  | 88 | | 0.5497 | 12.7400 | 7.2014 | 5.3202 | 0.0813 | 0.2042 | 0.1042 | 0.0800 | 0.1210 | 0.1086 | 1.2921 | 5.6010 | 1.1423 | 3.9579 | 0.1977 | 0.9606 | 1.0297 | 0.0686 | 0.9624 | 1.0300 |
|  |  | 120 | | 0.6010 | 11.7527 | 7.2079 | 5.4318 | 0.0698 | 0.1985 | 0.0907 | 0.0685 | 0.1036 | 0.0937 | 1.2311 | 6.1145 | 1.1181 | 4.0269 | 0.1767 | 1.0598 | 1.0117 | 0.0594 | 1.0639 | 1.0223 |
|  |  | 184 | | 0.7749 | 10.9261 | 8.9418 | 5.4223 | 0.0557 | 0.1599 | 0.0725 | 0.0535 | 0.0822 | 0.0737 | 1.2359 | 6.2992 | 1.1089 | 4.1021 | 0.1451 | 1.0081 | 1.0649 | 0.0455 | 1.0111 | 1.0657 |
|  |  | 312 | | 0.8253 | 9.2462 | 8.3583 | 5.3445 | 0.0418 | 0.1320 | 0.0545 | 0.0421 | 0.0640 | 0.0573 | 1.1790 | 6.3386 | 1.0812 | 4.5946 | 0.1037 | 0.9966 | 0.9603 | 0.0362 | 0.9825 | 0.9712 |

*=In this table the colour red is for figures far to 1 and green are for figures closer to 1. As 1 is the precise estimate of variance. Values lower than 1 are underestimation of the real variance and over 1 are over estimations.

**Coverage**

|  |  | **Controlled segmented regression (CITS)** | | | | **DiD framework segmented regression** | | | | **ITS of the difference** | | | | | | **control as covariate**  **uncontrolled** | | | **uncontrolled** | | |
| --- | --- | --- | --- | --- | --- | --- | --- | --- | --- | --- | --- | --- | --- | --- | --- | --- | --- | --- | --- | --- | --- |
| Parallel scenario | # data points | Panel FE T | Panel FE,DK T | panel FGLS T | Panel FE splnT | Panel FE T | Panel FE,DK T | panel FGLS T | Panel FE splnT | diff | diff | diff T | diff X T | diff splnT | diff X splnT | ols | Ols C T | ols C splnT | ols | Ols C T | ols C splnT |
| UnParallel trend | 24 | 0.3433 | 1.0000 | 1.0000 | 1.0000 | 0.1400 | 0.1633 | 0.1333 | 0.1233 | 0.1733 | 0.1433 | 0.9600 | 1.0000 | 0.8200 | 0.2633 | 0.4233 | 0.0000 | 0.0000 | 0.0000 | 0.0000 | 0.0000 |
|  | 32 | 0.1467 | 1.0000 | 0.0900 | 0.0233 | 0.0600 | 0.1000 | 0.0533 | 0.0500 | 0.1033 | 0.0767 | 0.7533 | 0.6800 | 0.9700 | 1.0000 | 0.2867 | 0.0000 | 0.0000 | 0.0000 | 0.0000 | 0.0000 |
|  | 40 | 0.1733 | 1.0000 | 0.9967 | 0.9100 | 0.0600 | 0.0867 | 0.0567 | 0.0467 | 0.0867 | 0.0733 | 0.9300 | 0.9967 | 0.9667 | 0.9900 | 0.3633 | 0.0000 | 0.0000 | 0.0000 | 0.0000 | 0.0000 |
|  | 48 | 0.0700 | 1.0000 | 0.0167 | 0.0000 | 0.0233 | 0.0533 | 0.0233 | 0.0200 | 0.0600 | 0.0433 | 0.7400 | 0.2033 | 0.9633 | 1.0000 | 0.2467 | 0.0000 | 0.0000 | 0.0000 | 0.0000 | 0.0000 |
|  | 56 | 0.4233 | 1.0000 | 1.0000 | 1.0000 | 0.2633 | 0.5500 | 0.2333 | 0.1600 | 0.3733 | 0.3800 | 1.0000 | 1.0000 | 1.0000 | 1.0000 | 0.0167 | 0.0000 | 0.0000 | 0.0000 | 0.0000 | 0.0000 |
|  | 72 | 0.0167 | 1.0000 | 0.0000 | 0.0000 | 0.0100 | 0.0467 | 0.0100 | 0.0100 | 0.0467 | 0.0167 | 0.6000 | 0.0000 | 0.9567 | 1.0000 | 0.2233 | 0.0000 | 0.0000 | 0.0000 | 0.0000 | 0.0000 |
|  | 88 | 0.0067 | 1.0000 | 0.0000 | 0.0000 | 0.0033 | 0.0267 | 0.0033 | 0.0000 | 0.0167 | 0.0033 | 0.3100 | 0.0000 | 0.9700 | 1.0000 | 0.1967 | 0.0000 | 0.0000 | 0.0000 | 0.0000 | 0.0000 |
|  | 120 | 0.0000 | 1.0000 | 0.0000 | 0.0000 | 0.0000 | 0.0333 | 0.0000 | 0.0000 | 0.0200 | 0.0000 | 0.2133 | 0.0000 | 0.9633 | 1.0000 | 0.1600 | 0.0000 | 0.0000 | 0.0000 | 0.0000 | 0.0000 |
|  | 184 | 0.0000 | 0.9467 | 0.0000 | 0.0000 | 0.0000 | 0.0133 | 0.0000 | 0.0000 | 0.0067 | 0.0000 | 0.0633 | 0.0000 | 0.9700 | 1.0000 | 0.1767 | 0.0000 | 0.0000 | 0.0000 | 0.0000 | 0.0000 |
|  | 312 | 0.0000 | 0.0000 | 0.0000 | 0.0000 | 0.0000 | 0.0000 | 0.0000 | 0.0000 | 0.0000 | 0.0000 | 0.0033 | 0.0000 | 0.9533 | 1.0000 | 0.0933 | 0.0000 | 0.0000 | 0.0000 | 0.0000 | 0.0000 |

Colours rank the performance relative to each sample size, from green (Coverage closest to 1 -optimal situation) to red (coverage further from 0).

## A10. Estimation of the effect (and bias) from the separate analysis of the intervention and control series.

| Autocorrelated error scenario | | | | | | | | | |  | Independent error scenario | | | | | | | | | | | |  | irregular unparallel trend between groups scenario | | | | | | | | | | | | | | | | |
| --- | --- | --- | --- | --- | --- | --- | --- | --- | --- | --- | --- | --- | --- | --- | --- | --- | --- | --- | --- | --- | --- | --- | --- | --- | --- | --- | --- | --- | --- | --- | --- | --- | --- | --- | --- | --- | --- | --- | --- | --- |
|  | estimates | | | | |  | |  | | Heteroscedastic scenario | | estimates | | | | |  | | |  | | Heteroscedastic scenario | | estimates | | | | | | | | | |  |  |  |  |  |  |  |
| Heteroscedastic scenario | Parallel scenario | # data points | single ITS intervention | single ITS control | difference |  | bias | |  |  |  | Parallel scenario | # data points | single ITS intervention (arima MA (1)) | single ITS control (arima MA (1)) | difference | |  | bias | |  |  |  | Parallel scenario | # data points | | single ITS intervention | | | single ITS control | | | difference | |  | | bias | | | |
| Homoscedastic | Parallel trend | 24 | -2.9992 | -0.9979 | -2.0013 |  | -0.0013 | |  | Homoscedastic | | Parallel trend | 24 | -2.9971 | -0.9949 | -2.0023 | |  | -0.0023 | |  | Homoscedastic | | Irregular unparallel trend | 24 | | -10.1801 | | | -2.0501 | | | -8.1300 | |  | | -6.1300 | | | |
|  |  | 32 | -3.0017 | -0.9991 | -2.0026 |  | -0.0026 | |  |  |  |  | 32 | -3.0038 | -0.9982 | -2.0056 | |  | -0.0056 | |  |  |  |  | 32 | | -10.3408 | | | -0.7867 | | | -9.5542 | |  | | -7.5542 | | | |
|  |  | 40 | -3.0023 | -1.0008 | -2.0015 |  | -0.0015 | |  |  |  |  | 40 | -3.0043 | -1.0003 | -2.0040 | |  | -0.0040 | |  |  |  |  | 40 | | -10.6301 | | | -1.2816 | | | -9.3485 | |  | | -7.3485 | | | |
|  |  | 48 | -2.9952 | -1.0005 | -1.9946 |  | 0.0054 | |  |  |  |  | 48 | -2.9909 | -1.0019 | -1.9891 | |  | 0.0109 | |  |  |  |  | 48 | | -11.1044 | | | -1.6650 | | | -9.4394 | |  | | -7.4394 | | | |
|  |  | 56 | -2.9991 | -0.9985 | -2.0007 |  | -0.0007 | |  |  |  |  | 56 | -2.9971 | -0.9967 | -2.0004 | |  | -0.0004 | |  |  |  |  | 56 | | -8.1678 | | | 2.0484 | | | -10.2162 | |  | | -8.2162 | | | |
|  |  | 72 | -2.9991 | -0.9997 | -1.9993 |  | 0.0007 | |  |  |  |  | 72 | -2.9993 | -0.9997 | -1.9996 | |  | 0.0004 | |  |  |  |  | 72 | | -10.6273 | | | -1.2435 | | | -9.3838 | |  | | -7.3838 | | | |
|  |  | 88 | -3.0004 | -1.0009 | -1.9995 |  | 0.0005 | |  |  |  |  | 88 | -3.0007 | -1.0004 | -2.0002 | |  | -0.0002 | |  |  |  |  | 88 | | -10.9866 | | | -1.5090 | | | -9.4776 | |  | | -7.4776 | | | |
|  |  | 120 | -3.0000 | -1.0004 | -1.9995 |  | 0.0005 | |  |  |  |  | 120 | -2.9996 | -1.0007 | -1.9988 | |  | 0.0012 | |  |  |  |  | 120 | | -11.7190 | | | -1.9345 | | | -9.7845 | |  | | -7.7845 | | | |
|  |  | 184 | -3.0008 | -1.0004 | -2.0004 |  | -0.0004 | |  |  |  |  | 184 | -3.0011 | -1.0006 | -2.0005 | |  | -0.0005 | |  |  |  |  | 184 | | -9.1826 | | | 0.3940 | | | -9.5766 | |  | | -7.5766 | | | |
|  |  | 312 | -2.9993 | -0.9997 | -1.9996 |  | 0.0004 | |  |  |  |  | 312 | -2.9987 | -0.9996 | -1.9991 | |  | 0.0009 | |  |  |  |  | 312 | | -11.0329 | | | -1.6305 | | | -9.4023 | |  | | -7.4023 | | | |
|  | Unparallel trend | 24 | -10.1801 | -1.6481 | -8.5320 |  | -6.5320 | |  |  |  | Unparallel trend | 24 | -9.2740 | -1.5370 | -7.7370 | |  | -5.7370 | |  |  | | | |  | | |  | | |  | | | |  | | |  |  |
|  |  | 32 | -10.3408 | -0.8999 | -9.4409 |  | -7.4409 | |  |  |  |  | 32 | -9.4787 | -0.8852 | -8.5935 | |  | -6.5935 | |  |  | | | | | |  | | |  | | | | |  | |  | |  |
|  |  | 40 | -10.6301 | -1.2070 | -9.4231 |  | -7.4231 | |  |  |  |  | 40 | -9.7974 | -1.1856 | -8.6118 | |  | -6.6118 | |  |  | | | | | |  | | |  | | | | |  | |  | |  |
|  |  | 48 | -11.1044 | -1.8144 | -9.2900 |  | -7.2900 | |  |  |  |  | 48 | -10.2637 | -1.6980 | -8.5657 | |  | -6.5657 | |  |  | | | | | |  | | |  | | | | |  | |  | |  |
|  |  | 56 | -8.1678 | 1.0656 | -9.2334 |  | -7.2334 | |  |  |  |  | 56 | -7.6626 | 0.8567 | -8.5193 | |  | -6.5193 | |  |  | | | | | |  | | |  | | | | |  | |  | |  |
|  |  | 72 | -10.6273 | -1.5440 | -9.0833 |  | -7.0833 | |  |  |  |  | 72 | -9.9945 | -1.4964 | -8.4981 | |  | -6.4981 | |  |  | | | | | |  | | |  | | | | |  | |  | |  |
|  |  | 88 | -10.9866 | -1.9681 | -9.0185 |  | -7.0185 | |  |  |  |  | 88 | -10.3684 | -1.8952 | -8.4732 | |  | -6.4732 | |  |  | | | | | |  | | |  | | | | |  | |  | |  |
|  |  | 120 | -11.7190 | -2.3544 | -9.3646 |  | -7.3646 | |  |  |  |  | 120 | -11.1162 | -2.2623 | -8.8539 | |  | -6.8539 | |  |  | | | | | |  | | |  | | | | |  | |  | |  |
|  |  | 184 | -9.1826 | -0.0722 | -9.1104 |  | -7.1104 | |  |  |  |  | 184 | -8.8344 | -0.1196 | -8.7148 | |  | -6.7148 | |  |  | | | | | |  | | |  | | | | |  | |  | |  |
|  |  | 312 | -11.0329 | -2.1673 | -8.8656 |  | -6.8656 | |  |  |  |  | 312 | -10.6785 | -2.1185 | -8.5600 | |  | -6.5600 | |  |  | | | | | |  | | |  | | | | |  | |  | |  |
| Heteroscedastic (control higher variance) | Parallel trend | 24 | -3.0871 | -1.0052 | -2.0819 |  | -0.0819 | |  | Heteroscedastic (control higher variance) | | Parallel trend | 24 | -3.0994 | -1.0156 | -2.0837 | |  | -0.0837 | |  |  | | | | | |  | | |  | | | | |  | |  | |  |
|  |  | 32 | -2.9846 | -0.9397 | -2.0450 |  | -0.0450 | |  |  |  |  | 32 | -2.9816 | -0.8522 | -2.1294 | |  | -0.1294 | |  |  | | | | | |  | | |  | | | | |  | |  | |  |
|  |  | 40 | -2.9907 | -0.9466 | -2.0441 |  | -0.0441 | |  |  |  |  | 40 | -2.9765 | -0.9643 | -2.0123 | |  | -0.0123 | |  |  | | | | | |  | | |  | | | | |  | |  | |  |
|  |  | 48 | -3.0426 | -1.0263 | -2.0163 |  | -0.0163 | |  |  |  |  | 48 | -3.1116 | -0.9628 | -2.1487 | |  | -0.1487 | |  |  | | | | | |  | | |  | | | | |  | |  | |  |
|  |  | 56 | -2.9836 | -1.0592 | -1.9243 |  | 0.0757 | |  |  |  |  | 56 | -2.9743 | -1.0824 | -1.8919 | |  | 0.1081 | |  |  | | | | | |  | | |  | | | | |  | |  | |  |
|  |  | 72 | -3.0192 | -1.0022 | -2.0171 |  | -0.0171 | |  |  |  |  | 72 | -3.0147 | -1.0216 | -1.9932 | |  | 0.0068 | |  |  | | | | | |  | | |  | | | | |  | |  | |  |
|  |  | 88 | -2.9767 | -0.9471 | -2.0296 |  | -0.0296 | |  |  |  |  | 88 | -3.0147 | -1.0216 | -1.9932 | |  | 0.0068 | |  |  | | | | | |  | | |  | | | | |  | |  | |  |
|  |  | 120 | -3.0077 | -0.9483 | -2.0595 |  | -0.0595 | |  |  |  |  | 120 | -3.0147 | -0.9320 | -2.0827 | |  | -0.0827 | |  |  | | | | | |  | | |  | | | | |  | |  | |  |
|  |  | 184 | -3.0024 | -0.9768 | -2.0256 |  | -0.0256 | |  |  |  |  | 184 | -3.0026 | -0.9641 | -2.0386 | |  | -0.0386 | |  |  | | | | | |  | | |  | | | | |  | |  | |  |
|  |  | 312 | -2.9908 | -0.9910 | -1.9998 |  | 0.0002 | |  |  |  |  | 312 | -2.9818 | -0.9861 | -1.9957 | |  | 0.0043 | |  |  | | | | | |  | | |  | | | | |  | |  | |  |
|  | Unparallel trend | 24 | -3.7526 | -8.2572 | 4.5046 |  | 6.5046 | |  |  |  | Unparallel trend | 24 | -3.8676 | -7.2915 | 3.4239 | |  | 5.4239 | |  |  | | | | | |  | | |  | | | | |  | |  | |  |
|  |  | 32 | -2.8618 | -8.2582 | 5.3964 |  | 7.3964 | |  |  |  |  | 32 | -2.9075 | -7.3215 | 4.4140 | |  | 6.4140 | |  |  | | | | | |  | | |  | | | | |  | |  | |  |
|  |  | 40 | -3.2075 | -8.5133 | 5.3057 |  | 7.3057 | |  |  |  |  | 40 | -3.1941 | -7.7396 | 4.5456 | |  | 6.5456 | |  |  | | | | | |  | | |  | | | | |  | |  | |  |
|  |  | 48 | -3.8639 | -9.1530 | 5.2890 |  | 7.2890 | |  |  |  |  | 48 | -3.9363 | -8.4130 | 4.4766 | |  | 6.4766 | |  |  | | | | | |  | | |  | | | | |  | |  | |  |
|  |  | 56 | -0.8989 | -6.2568 | 5.3580 |  | 7.3580 | |  |  |  |  | 56 | -1.1089 | -5.8228 | 4.7139 | |  | 6.7139 | |  |  | | | | | |  | | |  | | | | |  | |  | |  |
|  |  | 72 | -3.5787 | -8.6505 | 5.0718 |  | 7.0718 | |  |  |  |  | 72 | -3.5326 | -8.0990 | 4.5664 | |  | 6.5664 | |  |  | | | | | |  | | |  | | | | |  | |  | |  |
|  |  | 88 | -3.9194 | -8.8962 | 4.9768 |  | 6.9768 | |  |  |  |  | 88 | -3.8881 | -8.3913 | 4.5032 | |  | 6.5032 | |  |  | | | | | |  | | |  | | | | |  | |  | |  |
|  |  | 120 | -4.3629 | -9.6521 | 5.2892 |  | 7.2892 | |  |  |  |  | 120 | -4.3418 | -9.2717 | 4.9299 | |  | 6.9299 | |  |  | | | | | |  | | |  | | | | |  | |  | |  |
|  |  | 184 | -2.0715 | -7.1586 | 5.0872 |  | 7.0872 | |  |  |  |  | 184 | -2.1513 | -6.9528 | 4.8015 | |  | 6.8015 | |  |  | | | | | |  | | |  | | | | |  | |  | |  |
|  |  | 312 | -4.1548 | -9.0100 | 4.8552 |  | 6.8552 | |  |  |  |  | 312 | -4.1492 | -8.8463 | 4.6971 | |  | 6.6971 | |  |  | | | | | |  | | |  | | | | |  | |  | |  |
| Heteroscedastic (intervention higher variance) | Parallel trend | 24 | -3.0106 | -1.0860 | -1.9247 |  | 0.0753 | |  | Heteroscedastic (intervention higher variance) | | Parallel trend | 24 | -3.0625 | -1.1384 | -1.9241 | |  | 0.0759 | |  |  | | | | | |  | | |  | | | | |  | |  | |  |
|  |  | 32 | -2.9272 | -0.9934 | -1.9337 |  | 0.0663 | |  |  |  |  | 32 | -2.9374 | -0.9631 | -1.9743 | |  | 0.0257 | |  |  | | | | | |  | | |  | | | | |  | |  | |  |
|  |  | 40 | -2.9378 | -0.9921 | -1.9456 |  | 0.0544 | |  |  |  |  | 40 | -2.9495 | -0.9960 | -1.9535 | |  | 0.0465 | |  |  | | | | | |  | | |  | | | | |  | |  | |  |
|  |  | 48 | -3.0152 | -1.0451 | -1.9701 |  | 0.0299 | |  |  |  |  | 48 | -2.9665 | -1.0964 | -1.8701 | |  | 0.1299 | |  |  | | | | | |  | | |  | | | | |  | |  | |  |
|  |  | 56 | -3.0597 | -0.9781 | -2.0816 |  | -0.0816 | |  |  |  |  | 56 | -3.0881 | -0.9714 | -2.1167 | |  | -0.1167 | |  |  | | | | | |  | | |  | | | | |  | |  | |  |
|  |  | 72 | -3.0186 | -1.0170 | -2.0016 |  | -0.0016 | |  |  |  |  | 72 | -3.0316 | -1.0278 | -2.0037 | |  | -0.0037 | |  |  | | | | | |  | | |  | | | | |  | |  | |  |
|  |  | 88 | -2.9435 | -0.9795 | -1.9639 |  | 0.0361 | |  |  |  |  | 88 | -2.9038 | -0.9530 | -1.9507 | |  | 0.0493 | |  |  | | | | | |  | | |  | | | | |  | |  | |  |
|  |  | 120 | -2.9578 | -1.0052 | -1.9526 |  | 0.0474 | |  |  |  |  | 120 | -2.9447 | -1.0104 | -1.9343 | |  | 0.0657 | |  |  | | | | | |  | | |  | | | | |  | |  | |  |
|  |  | 184 | -2.9782 | -1.0021 | -1.9761 |  | 0.0239 | |  |  |  |  | 184 | -2.9686 | -1.0029 | -1.9657 | |  | 0.0343 | |  |  | | | | | |  | | |  | | | | |  | |  | |  |
|  |  | 312 | -2.9896 | -0.9906 | -1.9990 |  | 0.0010 | |  |  |  |  | 312 | -2.9796 | -0.9826 | -1.9970 | |  | 0.0030 | |  |  | | | | | |  | | |  | | | | |  | |  | |  |
|  | Unparallel trend | 24 | -10.1893 | -1.7853 | -8.4041 |  | -6.4041 | |  |  |  | Unparallel trend | 24 | -9.1575 | -1.7853 | -7.3722 | |  | -5.3722 | |  |  | | | | | |  | | |  | | | | |  | |  | |  |
|  |  | 32 | -10.2347 | -0.8895 | -9.3453 |  | -7.3453 | |  |  |  |  | 32 | -9.3012 | -0.8937 | -8.4075 | |  | -6.4075 | |  |  | | | | | |  | | |  | | | | |  | |  | |  |
|  |  | 40 | -10.5153 | -1.1949 | -9.3204 |  | -7.3204 | |  |  |  |  | 40 | -9.7999 | -1.1804 | -8.6194 | |  | -6.6194 | |  |  | | | | | |  | | |  | | | | |  | |  | |  |
|  |  | 48 | -11.1374 | -1.8835 | -9.2538 |  | -7.2538 | |  |  |  |  | 48 | -10.3086 | -1.9093 | -8.3993 | |  | -6.3993 | |  |  | | | | | |  | | |  | | | | |  | |  | |  |
|  |  | 56 | -8.2380 | 1.0990 | -9.3371 |  | -7.3371 | |  |  |  |  | 56 | -7.7439 | 0.9119 | -8.6558 | |  | -6.6558 | |  |  | | | | | |  | | |  | | | | |  | |  | |  |
|  |  | 72 | -10.6665 | -1.5686 | -9.0978 |  | -7.0978 | |  |  |  |  | 72 | -10.1370 | -1.5362 | -8.6008 | |  | -6.6008 | |  |  | | | | | |  | | |  | | | | |  | |  | |  |
|  |  | 88 | -10.8868 | -1.9323 | -8.9545 |  | -6.9545 | |  |  |  |  | 88 | -10.3642 | -1.8484 | -8.5158 | |  | -6.5158 | |  |  | | | | | |  | | |  | | | | |  | |  | |  |
|  |  | 120 | -11.6535 | -2.3618 | -9.2917 |  | -7.2917 | |  |  |  |  | 120 | -11.2852 | -2.3410 | -8.9442 | |  | -6.9442 | |  |  | | | | | |  | | |  | | | | |  | |  | |  |
|  |  | 184 | -9.1506 | -0.0746 | -9.0760 |  | -7.0760 | |  |  |  |  | 184 | -8.9459 | -0.1469 | -8.7990 | |  | -6.7990 | |  |  | | | | | |  | | |  | | | | |  | |  | |  |
|  |  | 312 | -11.0146 | -2.1537 | -8.8609 |  | -6.8609 | |  |  |  |  | 312 | -10.8419 | -2.1496 | -8.6924 | |  | -6.6924 | |  |  | | | | | |  | | |  | | | | |  | |  | |  |

## A11. STATA code for base case scenario and 24 observations: parallel trend, no error autocorrelation, homoscedastic

clear

capture program drop beta

program beta, rclass

clear

*24 periods, 12 pre and 12 post in 2 groups

set obs 48

gen seq=_n

*every period per group

egen seq_=seq(), f(1) t(24)

gen treat=1 if seq<=24

replace treat=0 if seq>24

gen post=1 if seq_>12

replace post=0 if post==.

*for each period, time-varying components of observed covariates are drawn from standard normal disrtibutions with mean the same mean of interveniton group and sd same sd (10, 3.5)

drawnorm x1_t, mean(10) sds(3.5)

replace x1_t=. if treat==0

*building the control based on the correlation between the series

local corr=0.2

local s2=3.5^2*(1/`corr'^2-1)

gen x2_t=x1_t+sqrt(`s2')*invnorm(uniform())

corr x1_t x2_t

*standardisation of varibales to keep same variance

quietly sum x1_t

local sz1=r(sd)

local mz1=r(mean)

g x1_tsd=(x1_t-`mz1')/`sz1'

quietly sum x2_t

local sz2=r(sd)

local mz2=r(mean)

g x2_tsd=(x2_t-`mz2')/`sz2'

corr x1_tsd x2_tsd

sort seq_ treat

replace x2_tsd=x2_tsd[_n+1] if x2_tsd==.

replace x1_tsd=x2_tsd if x1_tsd==.

drop x1_t x2_t

rename x1_tsd x1_t

drop x2_t

*building the time invariant varibale based on the average of Glasgow city (10.48) and Edinburgh City series (3.16) pre intervention on average, difference of 7.3

*norm with average Glasgow

gen u1_t_=rnormal(0,20)

gen stack=abs(rnormal(7.3,7.3))

replace u1_t_=. if seq!=1

replace stack=. if seq!=1

egen u1_t__=max(u1_t_)

egen stack_=max(stack)

replace u1_t__=u1_t__+stack_ if treat==1

drop u1_t_ stack_ stack_

rename u1_t__ u1_t

*lambda

gen lambda_t=1/*(1+0.55*(1-(seq_-24)/16)+.2*sin(2*_pi/26))*/

drawnorm e_it, mean(0) sd(0.1)

gen p_t=(post*treat)

tsset treat seq_

****************************************************

*build confounders

g conf=post+rnormal(0,0.01)

*data generating process

*intervention

gen Y_it=1*x1_t-2*(p_t)-1*conf+lambda_t*u1_t+e_it

*introducing underlying slope

g inter=(seq_*treat)

g trend= seq_-1

*cubic splines

mkspline T3_= seq_, cubic nknot(3)

*CITS panel FEspln

*Panel FE spln

xtreg Y_it x1_t T3_* inter p_t post trend, fe

return scalar b_panelFEs1=_b[p_t]

return scalar e_panelFEs1=_se[p_t]

return scalar b_panelFEsLB1=_r_lb[p_t]

return scalar b_panelFEsUB1=_r_ub[p_t]

*Panel FE

xtreg Y_it x1_t inter p_t post trend, fe

return scalar b_panelFE1=_b[p_t]

return scalar e_panelFE1=_se[p_t]

return scalar b_panelFELB1=_r_lb[p_t]

return scalar b_panelFEUB1=_r_ub[p_t]

*Panel FGLS

xtgls Y_it x1_t p_t post treat inter trend, panels(correlated)

return scalar b_gls1=_b[p_t]

return scalar e_gls1=_se[p_t]

return scalar b_glsLB1=_r_lb[p_t]

return scalar b_glsUB1=_r_ub[p_t]

*Panel Driscoll-Kraay

xtscc Y_it x1_t p_t post inter trend, fe

return scalar b_DK1=_b[p_t]

return scalar e_DK1=_se[p_t]

return scalar b_DKLB1=_r_lb[p_t]

return scalar b_DKUB1=_r_ub[p_t]

*panel data structure DiD

*panel FEspln

xtreg Y_it x1_t T3_* p_t post, fe

return scalar b_panelFEs=_b[p_t]

return scalar e_panelFEs=_se[p_t]

return scalar b_panelFEsLB=_r_lb[p_t]

return scalar b_panelFEsUB=_r_ub[p_t]

*panel FE

xtreg Y_it x1_t p_t post, fe

return scalar b_panelFE=_b[p_t]

return scalar e_panelFE=_se[p_t]

return scalar b_panelFELB=_r_lb[p_t]

return scalar b_panelFEUB=_r_ub[p_t]

*panel FGLS

xtgls Y_it x1_t p_t post treat trend, panels(correlated)

return scalar b_gls=_b[p_t]

return scalar e_gls=_se[p_t]

return scalar b_glsLB=_r_lb[p_t]

return scalar b_glsUB=_r_ub[p_t]

*panel Driscoll-Kraay

xtscc Y_it x1_t p_t post, fe

return scalar b_DK=_b[p_t]

return scalar e_DK=_se[p_t]

return scalar b_DKLB=_r_lb[p_t]

return scalar b_DKUB=_r_ub[p_t]

*difference

*make it as a time series

drop seq u1_t e_it p_t conf stack T* inter

reshape wide Y_it x1_t, i(seq_) j(treat)

gen diff=Y_it1-Y_it0

gen diff_X=x1_t1-x1_t0

*difference ols

reg diff post trend

return scalar b_diff=_b[post]

return scalar e_diff=_se[post]

return scalar b_diffLB=_r_lb[post]

return scalar b_diffUB=_r_ub[post]

*difference ols with covariateas

reg diff diff_X post trend

return scalar b_diffX=_b[post]

return scalar e_diffX=_se[post]

return scalar b_diffXLB=_r_lb[post]

return scalar b_diffXUB=_r_ub[post]

*cubic splines

mkspline T3= seq_, cubic nknot(3)

tsset seq_

*Difference OLS controlled

reg diff T3* post

return scalar b_diff_spln=_b[post]

return scalar e_diff_spln=_se[post]

return scalar b_diff_splnLB=_r_lb[post]

return scalar b_diff_splnUB=_r_ub[post]

*cubic spline with differnce in covariates

reg diff T3* diff_X post

return scalar b_diffX_spln=_b[post]

return scalar e_diffX_spln=_se[post]

return scalar b_diffX_splnLB=_r_lb[post]

return scalar b_diffX_splnUB=_r_ub[post]

*control as covariate with previous covariates

*OLS

reg Y_it1 Y_it0 x1_t1 post trend

return scalar b_con_cov=_b[post]

return scalar e_con_cov=_se[post]

return scalar b_con_covLB=_r_lb[post]

return scalar b_con_covUB=_r_ub[post]

*control as covariate without previous covariates

reg Y_it1 Y_it0 post trend

return scalar b_con_covX=_b[post]

return scalar e_con_covX=_se[post]

return scalar b_con_covLBX=_r_lb[post]

return scalar b_con_covUBX=_r_ub[post]

*cubic splines with previous covariates

reg Y_it1 T3* x1_t1 Y_it0 post

return scalar b_spl_cov=_b[post]

return scalar e_spl_cov=_se[post]

return scalar b_spl_covLB=_r_lb[post]

return scalar b_spl_covUB=_r_ub[post]

*cubic splines without previous covariates

reg Y_it1 T3* Y_it0 post

return scalar b_spl_covX=_b[post]

return scalar e_spl_covX=_se[post]

return scalar b_spl_covLBX=_r_lb[post]

return scalar b_spl_covUBX=_r_ub[post]

*cubic splines and spline of control as covariate

mkspline C3= Y_it0, cubic nknot(3)

reg Y_it1 T3* x1_t1 C3* post trend

estat ic

return scalar b_spl_splscov=_b[post]

return scalar e_spl_splcov=_se[post]

return scalar b_spl_splcovLB=_r_lb[post]

return scalar b_spl_splcovUB=_r_ub[post]

reg Y_it1 T3* C3* post trend

estat ic

return scalar b_spl_splscovX=_b[post]

return scalar e_spl_splcovX=_se[post]

return scalar b_spl_splcovLBX=_r_lb[post]

return scalar b_spl_splcovUBX=_r_ub[post]

*uncontrolled

*control as covariate

*OLS

reg Y_it1 x1_t1 post trend

return scalar b_con_covu=_b[post]

return scalar e_con_covu=_se[post]

return scalar b_con_covuLB=_r_lb[post]

return scalar b_con_covuUB=_r_ub[post]

tsset seq_

*cubic splines

reg Y_it1 T3* x1_t1 post

return scalar b_spl_covu=_b[post]

return scalar e_spl_covu=_se[post]

return scalar b_spl_covuLB=_r_lb[post]

return scalar b_spl_covuUB=_r_ub[post]

end

simulate /*panelFE CITS*/ b_panelFE1=r(b_panelFE1) e_panelFE1=r(e_panelFE1) b_panelFELB1=r(b_panelFELB1) b_panelFEUB1=r(b_panelFEUB1) /*panelFEs CITS */ b_panelFEs1=r(b_panelFEs1) e_panelFEs1=r(e_panelFEs1) b_panelFEsLB1=r(b_panelFEsLB1) b_panelFEsUB1=r(b_panelFEsUB1) /*gls CITS (use heteroskedastic and correlated error structure)*/ b_gls1=r(b_gls1) e_gls1=r(e_gls1) b_glsLB1=r(b_glsLB1) b_glsUB1=r(b_glsUB1) /*Driscoll-Kraay CITS */ b_DK1=r(b_DK1) e_DK1=r(e_DK1) b_DKLB1=r(b_DKLB1) b_DKUB1=r(b_DKUB1)/*panelFE*/ b_panelFE=r(b_panelFE) e_panelFE=r(e_panelFE) b_panelFELB=r(b_panelFELB) b_panelFEUB=r(b_panelFEUB) /*panelFEs*/ b_panelFEs=r(b_panelFEs) e_panelFEs=r(e_panelFEs) b_panelFEsLB=r(b_panelFEsLB) b_panelFEsUB=r(b_panelFEsUB) /*gls (use heteroskedastic and correlated error structure)*/ b_gls=r(b_gls) e_gls=r(e_gls) b_glsLB=r(b_glsLB) b_glsUB=r(b_glsUB) /*Driscoll-Kraay*/ b_DK=r(b_DK) e_DK=r(e_DK) b_DKLB=r(b_DKLB) b_DKUB=r(b_DKUB) /*ols on the difference*/ b_diff=r(b_diff) e_diff=r(e_diff) b_diffLB=r(b_diffLB) b_diffUB=r(b_diffUB) /*ols on the difference with covariate difference*/ b_diffX=r(b_diffX) e_diffX=r(e_diffX) b_diffXLB=r(b_diffXLB) b_diffXUB=r(b_diffXUB) /*ols difference with spline of time*/ b_diff_spln=r(b_diff_spln) e_diff_spln=r(e_diff_spln) b_diff_splnLB=r(b_diff_splnLB) b_diff_splnUB=r(b_diff_splnUB) /*difference with spline of time and difference in covariates*/ b_diffX_spln=r(b_diffX_spln) e_diffX_spln=r(e_diffX_spln) b_diffX_splnLB=r(b_diffX_splnLB) b_diffX_splnUB=r(b_diffX_splnUB) /*ols control as covariate*/ b_con_cov=r(b_con_cov) e_con_cov=r(e_con_cov) b_con_covLB=r(b_con_covLB) b_con_covUB=r(b_con_covUB) /*ols control as covariate*/ b_con_covX=r(b_con_covX) e_con_covX=r(e_con_covX) b_con_covLBX=r(b_con_covLBX) b_con_covUBX=r(b_con_covUBX) /*ols using spline of time*/ b_spl_covX=r(b_spl_covX) e_spl_covX=r(e_spl_covX) b_spl_covLBX=r(b_spl_covLBX) b_spl_covUBX=r(b_spl_covUBX) /*ols using spline of time*/ b_spl_cov=r(b_spl_cov) e_spl_cov=r(e_spl_cov) b_spl_covLB=r(b_spl_covLB) b_spl_covUB=r(b_spl_covUB) /*ols using spline of time and spline of control*/ b_spl_splscov=r(b_spl_splscov) e_spl_splcov=r(e_spl_splcov) b_spl_splcovLB=r(b_spl_splcovLB) b_spl_splcovUB=r(b_spl_splcovUB)/*ols using spline of time and spline of control*/ b_spl_splscovX=r(b_spl_splscovX) e_spl_splcovX=r(e_spl_splcovX) b_spl_splcovLBX=r(b_spl_splcovLBX) b_spl_splcovUBX=r(b_spl_splcovUBX) /*uncontrolled ols control as covariate*/ b_con_covu=r(b_con_covu) e_con_covu=r(e_con_covu) b_con_covuLB=r(b_con_covuLB) b_con_covuUB=r(b_con_covuUB) /*ols using spline of time*/ b_spl_covu=r(b_spl_covu) e_spl_covu=r(e_spl_covu) b_spl_covuLB=r(b_spl_covuLB) b_spl_covuUB=r(b_spl_covuUB) , reps(300) seed(8391): beta

*bias

gen theta=-2

local estiamtes b_panelFE1 b_panelFEs1 b_gls1 b_DK1 b_panelFE b_panelFEs b_gls b_DK b_diff b_diffX b_diff_spln b_diffX_spln b_con_cov b_spl_cov b_spl_splscov b_con_covX b_spl_covX b_spl_splscovX b_con_covu b_spl_covu

foreach x of local estiamtes{

gen bias_`x'=`x'-theta

egen bias_`x'_=mean(bias_`x')

}

sum bias_*

*empMSE

local estiamtes b_panelFE1 b_panelFEs1 b_gls1 b_DK1 b_panelFE b_panelFEs b_gls b_DK b_diff b_diffX b_diff_spln b_diffX_spln b_con_cov b_spl_cov b_spl_splscov b_con_covX b_spl_covX b_spl_splscovX b_con_covu b_spl_covu

foreach x of local estiamtes{

egen mean_`x'=mean(`x')

gen _MSE_`x'_=(`x'-mean_`x')^2

egen _MSE_`x'=total(_MSE_`x'_)

gen MSE_`x'=sqrt(_MSE_`x'/(300-1))

}

drop mean* _MSE*

sum MSE*

*avgModSE

local se e_panelFE1 e_panelFEs1 e_gls1 e_DK1 e_panelFE e_panelFEs e_gls e_DK e_diff e_diffX e_diff_spln e_diffX_spln e_con_cov e_spl_cov e_spl_splcov e_con_covX e_spl_covX e_spl_splcovX e_con_covu e_spl_covu

foreach x of local se{

gen e2_`x'=(`x')^2

egen sum_`x'=total(`x')

gen avgModSE_`x'=sqrt(sum_`x'/(300-1))

}

*Coverage

gen coverage_panelFE1=1 if theta>=b_panelFELB1 & theta<=b_panelFEUB1

gen coverage_panelFEs1=1 if theta>=b_panelFEsLB1 & theta<=b_panelFEsUB1

gen coverage_gls1=1 if theta>=b_glsLB1 & theta<=b_glsUB1

gen coverage_DK1=1 if theta>=b_DKLB1 & theta<=b_DKUB1

gen coverage_panelFE=1 if theta>=b_panelFELB & theta<=b_panelFEUB

gen coverage_panelFEs=1 if theta>=b_panelFEsLB & theta<=b_panelFEsUB

gen coverage_gls=1 if theta>=b_glsLB & theta<=b_glsUB

gen coverage_DK=1 if theta>=b_DKLB & theta<=b_DKUB

gen coverage_diff=1 if theta>=b_diffLB & theta<=b_diffUB

gen coverage_diffX=1 if theta>=b_diffXLB & theta<=b_diffXUB

gen coverage_diff_spl=1 if theta>=b_diff_splnLB & theta<=b_diff_splnUB

gen coverage_diffX_spl=1 if theta>=b_diffX_splnLB & theta<=b_diffX_splnUB

gen coverage_con_cov=1 if theta>=b_con_covLB & theta<=b_con_covUB

gen coverage_spl_cov=1 if theta>=b_spl_covLB & theta<=b_spl_covUB

gen coverage_spl_splcov=1 if theta>=b_spl_splcovLB & theta<=b_spl_splcovUB

gen coverage_con_covX=1 if theta>=b_con_covLBX & theta<=b_con_covUBX

gen coverage_spl_covX=1 if theta>=b_spl_covLBX & theta<=b_spl_covUBX

gen coverage_spl_splcovX=1 if theta>=b_spl_splcovLBX & theta<=b_spl_splcovUBX

gen coverage_covu=1 if theta>=b_con_covuLB & theta<=b_con_covuUB

gen coverage_spl_covu=1 if theta>=b_spl_covuLB & theta<=b_spl_covuUB

local coverage coverage_panelFE coverage_panelFEs coverage_gls coverage_DK coverage_diff coverage_diffX coverage_diff_spl coverage_diffX_spl coverage_con_cov coverage_spl_cov coverage_spl_splcov coverage_con_covX coverage_spl_covX coverage_spl_splcovX coverage_spl_covu coverage_covu

foreach x of local coverage{

replace `x'=0 if `x'==.

egen `x'_=mean(`x')

}

sum coverage*

gen incremental_unit_diff=.

gen sigma2="0.1"

gen ar=0

gen delta_parallel=0

gen sample_size=24

collapse MSE* avgModSE_* *_ ,by(sample_size delta_parallel ar sigma2 incremental_unit_diff theta)

## A12. Montecarlo SE of Bias - independent errors scenario.

| Heteroscedastic scenario | Parallel scenario | # data points | **Controlled segmented regression (CITS)** | | | | **DiD framework segmented regression** | | | | | | **ITS of the difference** | | | | | | **control as covariate** | | | **Uncontrolled ITS** | | |
| --- | --- | --- | --- | --- | --- | --- | --- | --- | --- | --- | --- | --- | --- | --- | --- | --- | --- | --- | --- | --- | --- | --- | --- | --- |
|  |  |  | panel FE T | panelFE DK T | Panel FE splnT | panel FGLS T | panel FE | panelFE DK | panel FE T | panelFE DK T | Panel FE splnT | panel FGLS T | diff | diff X | diff T | diff X T | diff splnT | diff X splnT | Ols C | Ols C T | ols C splnT | Ols | Ols T | ols splnT |
| Homoscedastic | Parallel trend | 24 | 0.0071 | 0.0071 | 0.0071 | 0.0071 | 0.0032 | 0.0032 | 0.0032 | 0.0032 | 0.0032 | 0.0032 | 0.0299 | 0.0033 | 0.0592 | 0.0071 | 0.0592 | 0.0071 | 0.0028 | 0.0054 | 0.0054 | 0.0023 | 0.0049 | 0.0049 |
|  |  | 32 | 0.0059 | 0.0059 | 0.0059 | 0.0059 | 0.0031 | 0.0031 | 0.0031 | 0.0031 | 0.0031 | 0.0031 | 0.0256 | 0.0031 | 0.0527 | 0.0060 | 0.0527 | 0.0060 | 0.0025 | 0.0044 | 0.0044 | 0.0021 | 0.0042 | 0.0042 |
|  |  | 40 | 0.0050 | 0.0050 | 0.0050 | 0.0050 | 0.0026 | 0.0026 | 0.0026 | 0.0026 | 0.0026 | 0.0026 | 0.0220 | 0.0027 | 0.0438 | 0.0050 | 0.0438 | 0.0050 | 0.0020 | 0.0037 | 0.0038 | 0.0018 | 0.0036 | 0.0036 |
|  |  | 48 | 0.0046 | 0.0046 | 0.0046 | 0.0046 | 0.0024 | 0.0024 | 0.0024 | 0.0024 | 0.0024 | 0.0024 | 0.0218 | 0.0024 | 0.0427 | 0.0046 | 0.0427 | 0.0046 | 0.0019 | 0.0033 | 0.0033 | 0.0016 | 0.0032 | 0.0032 |
|  |  | 56 | 0.0043 | 0.0043 | 0.0043 | 0.0043 | 0.0021 | 0.0021 | 0.0021 | 0.0021 | 0.0021 | 0.0021 | 0.0197 | 0.0021 | 0.0414 | 0.0044 | 0.0414 | 0.0044 | 0.0018 | 0.0032 | 0.0032 | 0.0016 | 0.0030 | 0.0030 |
|  |  | 72 | 0.0040 | 0.0040 | 0.0040 | 0.0040 | 0.0021 | 0.0021 | 0.0021 | 0.0021 | 0.0021 | 0.0021 | 0.0171 | 0.0021 | 0.0329 | 0.0039 | 0.0329 | 0.0039 | 0.0016 | 0.0028 | 0.0028 | 0.0015 | 0.0027 | 0.0027 |
|  |  | 88 | 0.0036 | 0.0036 | 0.0036 | 0.0036 | 0.0018 | 0.0018 | 0.0018 | 0.0018 | 0.0018 | 0.0018 | 0.0151 | 0.0018 | 0.0312 | 0.0036 | 0.0312 | 0.0036 | 0.0014 | 0.0026 | 0.0027 | 0.0013 | 0.0026 | 0.0026 |
|  |  | 120 | 0.0029 | 0.0029 | 0.0029 | 0.0029 | 0.0016 | 0.0016 | 0.0016 | 0.0016 | 0.0016 | 0.0016 | 0.0140 | 0.0016 | 0.0275 | 0.0029 | 0.0275 | 0.0029 | 0.0012 | 0.0021 | 0.0021 | 0.0011 | 0.0020 | 0.0020 |
|  |  | 184 | 0.0024 | 0.0024 | 0.0024 | 0.0024 | 0.0012 | 0.0012 | 0.0012 | 0.0012 | 0.0012 | 0.0012 | 0.0104 | 0.0012 | 0.0221 | 0.0024 | 0.0221 | 0.0024 | 0.0010 | 0.0018 | 0.0018 | 0.0009 | 0.0017 | 0.0017 |
|  |  | 312 | 0.0019 | 0.0019 | 0.0019 | 0.0019 | 0.0009 | 0.0009 | 0.0009 | 0.0009 | 0.0009 | 0.0009 | 0.0080 | 0.0009 | 0.0178 | 0.0019 | 0.0178 | 0.0019 | 0.0007 | 0.0014 | 0.0014 | 0.0006 | 0.0013 | 0.0013 |
|  | Unparallel trend | 24 | 0.0071 | 0.0071 | 0.0071 | 0.0071 | 0.2690 | 0.2690 | 0.2682 | 0.2682 | 0.2682 | 0.2681 | 0.2688 | 0.2687 | 0.0592 | 0.0071 | 0.0592 | 0.0071 | 0.1505 | 0.0054 | 0.0054 | 0.9339 | 0.0049 | 0.0049 |
|  |  | 32 | 0.0059 | 0.0059 | 0.0059 | 0.0059 | 0.2930 | 0.2930 | 0.2901 | 0.2901 | 0.2901 | 0.2906 | 0.2884 | 0.2898 | 0.0527 | 0.0060 | 0.0527 | 0.0060 | 0.1686 | 0.0044 | 0.0044 | 0.9712 | 0.0042 | 0.0042 |
|  |  | 40 | 0.0050 | 0.0050 | 0.0050 | 0.0050 | 0.2851 | 0.2851 | 0.2844 | 0.2844 | 0.2844 | 0.2845 | 0.2855 | 0.2843 | 0.0438 | 0.0050 | 0.0438 | 0.0050 | 0.1773 | 0.0037 | 0.0038 | 1.0557 | 0.0036 | 0.0036 |
|  |  | 48 | 0.0046 | 0.0046 | 0.0046 | 0.0046 | 0.2767 | 0.2767 | 0.2767 | 0.2767 | 0.2767 | 0.2769 | 0.2759 | 0.2766 | 0.0427 | 0.0046 | 0.0427 | 0.0046 | 0.1823 | 0.0033 | 0.0033 | 0.9326 | 0.0032 | 0.0032 |
|  |  | 56 | 0.0043 | 0.0043 | 0.0043 | 0.0043 | 0.2849 | 0.2849 | 0.2855 | 0.2855 | 0.2855 | 0.2853 | 0.2866 | 0.2858 | 0.0414 | 0.0044 | 0.0414 | 0.0044 | 0.1705 | 0.0032 | 0.0032 | 1.0076 | 0.0030 | 0.0030 |
|  |  | 72 | 0.0040 | 0.0040 | 0.0040 | 0.0040 | 0.2895 | 0.2895 | 0.2896 | 0.2896 | 0.2896 | 0.2898 | 0.2888 | 0.2894 | 0.0329 | 0.0039 | 0.0329 | 0.0039 | 0.1501 | 0.0028 | 0.0028 | 0.9959 | 0.0027 | 0.0027 |
|  |  | 88 | 0.0036 | 0.0036 | 0.0036 | 0.0036 | 0.2875 | 0.2875 | 0.2872 | 0.2872 | 0.2872 | 0.2870 | 0.2871 | 0.2874 | 0.0312 | 0.0036 | 0.0312 | 0.0036 | 0.1924 | 0.0026 | 0.0027 | 1.0320 | 0.0026 | 0.0026 |
|  |  | 120 | 0.0029 | 0.0029 | 0.0029 | 0.0029 | 0.2933 | 0.2933 | 0.2933 | 0.2933 | 0.2933 | 0.2935 | 0.2939 | 0.2931 | 0.0275 | 0.0029 | 0.0275 | 0.0029 | 0.1839 | 0.0021 | 0.0021 | 1.0285 | 0.0020 | 0.0020 |
|  |  | 184 | 0.0024 | 0.0024 | 0.0024 | 0.0024 | 0.2919 | 0.2919 | 0.2919 | 0.2919 | 0.2919 | 0.2919 | 0.2917 | 0.2919 | 0.0221 | 0.0024 | 0.0221 | 0.0024 | 0.1708 | 0.0018 | 0.0018 | 1.0135 | 0.0017 | 0.0017 |
|  |  | 312 | 0.0019 | 0.0019 | 0.0019 | 0.0019 | 0.2914 | 0.2914 | 0.2915 | 0.2915 | 0.2915 | 0.2915 | 0.2921 | 0.2915 | 0.0178 | 0.0019 | 0.0178 | 0.0019 | 0.1861 | 0.0014 | 0.0014 | 0.9973 | 0.0013 | 0.0013 |
| Heteroscedastic (control higher variance) | Parallel trend | 24 | 0.2305 | 0.2305 | 0.2302 | 0.2305 | 0.1022 | 0.1022 | 0.1021 | 0.1021 | 0.1021 | 0.1005 | 0.1032 | 0.1030 | 0.2324 | 0.2374 | 0.2324 | 0.2372 | 0.0483 | 0.1117 | 0.1126 | 0.0461 | 0.1083 | 0.1086 |
|  |  | 32 | 0.1927 | 0.1927 | 0.1927 | 0.1923 | 0.0879 | 0.0879 | 0.0879 | 0.0879 | 0.0880 | 0.0880 | 0.0905 | 0.0895 | 0.1940 | 0.1974 | 0.1940 | 0.1972 | 0.0420 | 0.0820 | 0.0830 | 0.0403 | 0.0807 | 0.0810 |
|  |  | 40 | 0.1679 | 0.1679 | 0.1680 | 0.1676 | 0.0932 | 0.0932 | 0.0931 | 0.0931 | 0.0931 | 0.0937 | 0.0962 | 0.0939 | 0.1724 | 0.1673 | 0.1724 | 0.1673 | 0.0455 | 0.0793 | 0.0794 | 0.0451 | 0.0790 | 0.0791 |
|  |  | 48 | 0.1411 | 0.1411 | 0.1411 | 0.1423 | 0.0822 | 0.0822 | 0.0823 | 0.0823 | 0.0824 | 0.0820 | 0.0834 | 0.0814 | 0.1471 | 0.1388 | 0.1471 | 0.1389 | 0.0353 | 0.0727 | 0.0727 | 0.0343 | 0.0712 | 0.0712 |
|  |  | 56 | 0.1510 | 0.1510 | 0.1510 | 0.1510 | 0.0706 | 0.0706 | 0.0706 | 0.0706 | 0.0706 | 0.0706 | 0.0730 | 0.0712 | 0.1548 | 0.1505 | 0.1548 | 0.1506 | 0.0371 | 0.0654 | 0.0655 | 0.0357 | 0.0643 | 0.0642 |
|  |  | 72 | 0.1267 | 0.1267 | 0.1267 | 0.1269 | 0.0610 | 0.0610 | 0.0610 | 0.0610 | 0.0611 | 0.0610 | 0.0628 | 0.0614 | 0.1302 | 0.1276 | 0.1302 | 0.1276 | 0.0282 | 0.0535 | 0.0535 | 0.0277 | 0.0541 | 0.0541 |
|  |  | 88 | 0.1238 | 0.1238 | 0.1238 | 0.1241 | 0.0634 | 0.0634 | 0.0634 | 0.0634 | 0.0634 | 0.0635 | 0.0657 | 0.0637 | 0.1253 | 0.1239 | 0.1253 | 0.1238 | 0.0315 | 0.0647 | 0.0648 | 0.0311 | 0.0640 | 0.0641 |
|  |  | 120 | 0.1019 | 0.1019 | 0.1020 | 0.1017 | 0.0539 | 0.0539 | 0.0539 | 0.0539 | 0.0539 | 0.0537 | 0.0564 | 0.0541 | 0.1071 | 0.1036 | 0.1071 | 0.1037 | 0.0245 | 0.0467 | 0.0466 | 0.0243 | 0.0462 | 0.0462 |
|  |  | 184 | 0.0732 | 0.0732 | 0.0731 | 0.0730 | 0.0387 | 0.0387 | 0.0387 | 0.0387 | 0.0387 | 0.0386 | 0.0397 | 0.0386 | 0.0770 | 0.0738 | 0.0770 | 0.0738 | 0.0180 | 0.0326 | 0.0327 | 0.0178 | 0.0328 | 0.0328 |
|  |  | 312 | 0.0554 | 0.0554 | 0.0554 | 0.0554 | 0.0322 | 0.0322 | 0.0322 | 0.0322 | 0.0322 | 0.0321 | 0.0331 | 0.0323 | 0.0581 | 0.0555 | 0.0581 | 0.0555 | 0.0146 | 0.0287 | 0.0287 | 0.0148 | 0.0288 | 0.0288 |
|  | Unparallel trend | 24 | 0.3583 | 0.3583 | 0.3578 | 0.3586 | 0.3200 | 0.3200 | 0.3168 | 0.3168 | 0.3168 | 0.3151 | 0.3180 | 0.3171 | 0.3567 | 0.3687 | 0.3567 | 0.3684 | 0.5636 | 0.1744 | 0.1760 | 0.8900 | 0.1689 | 0.1693 |
|  |  | 32 | 0.2984 | 0.2984 | 0.2984 | 0.2981 | 0.3291 | 0.3291 | 0.3298 | 0.3298 | 0.3301 | 0.3305 | 0.3351 | 0.3302 | 0.2976 | 0.3057 | 0.2976 | 0.3052 | 0.5548 | 0.1272 | 0.1289 | 0.9329 | 0.1254 | 0.1259 |
|  |  | 40 | 0.2621 | 0.2621 | 0.2623 | 0.2617 | 0.3124 | 0.3124 | 0.3121 | 0.3121 | 0.3122 | 0.3118 | 0.3126 | 0.3119 | 0.2632 | 0.2609 | 0.2632 | 0.2610 | 0.5987 | 0.1229 | 0.1228 | 1.0073 | 0.1226 | 0.1227 |
|  |  | 48 | 0.2206 | 0.2206 | 0.2205 | 0.2226 | 0.3152 | 0.3152 | 0.3150 | 0.3150 | 0.3150 | 0.3151 | 0.3172 | 0.3136 | 0.2245 | 0.2171 | 0.2245 | 0.2172 | 0.5497 | 0.1133 | 0.1133 | 0.8921 | 0.1110 | 0.1110 |
|  |  | 56 | 0.2328 | 0.2328 | 0.2328 | 0.2329 | 0.3185 | 0.3185 | 0.3178 | 0.3178 | 0.3178 | 0.3175 | 0.3178 | 0.3192 | 0.2349 | 0.2319 | 0.2349 | 0.2321 | 0.5943 | 0.1009 | 0.1009 | 0.9685 | 0.0993 | 0.0993 |
|  |  | 72 | 0.1967 | 0.1967 | 0.1967 | 0.1971 | 0.3103 | 0.3103 | 0.3099 | 0.3099 | 0.3099 | 0.3097 | 0.3115 | 0.3098 | 0.1989 | 0.1981 | 0.1989 | 0.1981 | 0.5791 | 0.0829 | 0.0829 | 0.9434 | 0.0840 | 0.0840 |
|  |  | 88 | 0.1928 | 0.1928 | 0.1928 | 0.1934 | 0.3098 | 0.3098 | 0.3101 | 0.3101 | 0.3101 | 0.3101 | 0.3105 | 0.3107 | 0.1928 | 0.1928 | 0.1928 | 0.1927 | 0.6001 | 0.1015 | 0.1015 | 0.9868 | 0.1001 | 0.1002 |
|  |  | 120 | 0.1585 | 0.1585 | 0.1586 | 0.1581 | 0.3078 | 0.3078 | 0.3075 | 0.3075 | 0.3075 | 0.3074 | 0.3079 | 0.3076 | 0.1624 | 0.1612 | 0.1624 | 0.1612 | 0.5810 | 0.0726 | 0.0725 | 0.9536 | 0.0719 | 0.0719 |
|  |  | 184 | 0.1138 | 0.1138 | 0.1138 | 0.1136 | 0.2995 | 0.2995 | 0.2994 | 0.2994 | 0.2994 | 0.2994 | 0.2998 | 0.2991 | 0.1165 | 0.1148 | 0.1165 | 0.1148 | 0.5834 | 0.0509 | 0.0509 | 0.9527 | 0.0510 | 0.0511 |
|  |  | 312 | 0.0860 | 0.0860 | 0.0860 | 0.0862 | 0.2954 | 0.2954 | 0.2952 | 0.2952 | 0.2952 | 0.2952 | 0.2949 | 0.2952 | 0.0877 | 0.0862 | 0.0877 | 0.0862 | 0.5970 | 0.0448 | 0.0448 | 0.9673 | 0.0449 | 0.0449 |
| Heteroscedastic (intervention higher variance) | Parallel trend | 24 | 0.2334 | 0.1987 | 0.2337 | 0.2321 | 0.0992 | 0.0992 | 0.0992 | 0.0992 | 0.0993 | 0.0988 | 0.1050 | 0.1020 | 0.2405 | 0.2366 | 0.2405 | 0.2365 | 0.0996 | 0.2048 | 0.2059 | 0.0907 | 0.1917 | 0.1922 |
|  |  | 32 | 0.1940 | 0.1940 | 0.1942 | 0.1942 | 0.0890 | 0.0890 | 0.0891 | 0.0891 | 0.0890 | 0.0887 | 0.0929 | 0.0894 | 0.2040 | 0.1963 | 0.2040 | 0.1962 | 0.0875 | 0.1760 | 0.1771 | 0.0842 | 0.1767 | 0.1770 |
|  |  | 40 | 0.1628 | 0.1628 | 0.1627 | 0.1627 | 0.0940 | 0.0940 | 0.0940 | 0.0940 | 0.0940 | 0.0936 | 0.0960 | 0.0936 | 0.1687 | 0.1660 | 0.1687 | 0.1660 | 0.0813 | 0.1441 | 0.1434 | 0.0783 | 0.1426 | 0.1422 |
|  |  | 48 | 0.1386 | 0.1386 | 0.1387 | 0.1402 | 0.0788 | 0.0788 | 0.0788 | 0.0788 | 0.0787 | 0.0789 | 0.0823 | 0.0805 | 0.1470 | 0.1381 | 0.1470 | 0.1381 | 0.0733 | 0.1270 | 0.1269 | 0.0712 | 0.1220 | 0.1220 |
|  |  | 56 | 0.1497 | 0.1497 | 0.1498 | 0.1499 | 0.0707 | 0.0707 | 0.0707 | 0.0707 | 0.0707 | 0.0707 | 0.0729 | 0.0707 | 0.1570 | 0.1491 | 0.1570 | 0.1492 | 0.0711 | 0.1378 | 0.1383 | 0.0649 | 0.1336 | 0.1341 |
|  |  | 72 | 0.1269 | 0.1269 | 0.1270 | 0.1260 | 0.0611 | 0.0611 | 0.0610 | 0.0610 | 0.0610 | 0.0609 | 0.0635 | 0.0611 | 0.1310 | 0.1269 | 0.1310 | 0.1268 | 0.0597 | 0.1132 | 0.1130 | 0.0585 | 0.1153 | 0.1152 |
|  |  | 88 | 0.1225 | 0.1225 | 0.1226 | 0.1224 | 0.0625 | 0.0625 | 0.0625 | 0.0625 | 0.0625 | 0.0620 | 0.0631 | 0.0629 | 0.1277 | 0.1226 | 0.1277 | 0.1225 | 0.0573 | 0.1120 | 0.1120 | 0.0551 | 0.1099 | 0.1099 |
|  |  | 120 | 0.1018 | 0.1018 | 0.1017 | 0.1016 | 0.0532 | 0.0532 | 0.0532 | 0.0532 | 0.0532 | 0.0531 | 0.0538 | 0.0537 | 0.1024 | 0.1023 | 0.1024 | 0.1023 | 0.0477 | 0.0920 | 0.0919 | 0.0464 | 0.0905 | 0.0905 |
|  |  | 184 | 0.0731 | 0.0731 | 0.0731 | 0.0729 | 0.0380 | 0.0380 | 0.0380 | 0.0380 | 0.0380 | 0.0380 | 0.0397 | 0.0381 | 0.0750 | 0.0733 | 0.0750 | 0.0733 | 0.0358 | 0.0657 | 0.0657 | 0.0350 | 0.0648 | 0.0648 |
|  |  | 312 | 0.0547 | 0.0547 | 0.0547 | 0.0546 | 0.0320 | 0.0320 | 0.0320 | 0.0320 | 0.0320 | 0.0320 | 0.0330 | 0.0321 | 0.0571 | 0.0551 | 0.0571 | 0.0550 | 0.0282 | 0.0515 | 0.0515 | 0.0279 | 0.0512 | 0.0512 |
|  | Unparallel trend | 24 | 0.3625 | 0.3625 | 0.3631 | 0.3604 | 0.3149 | 0.3149 | 0.3150 | 0.3150 | 0.3150 | 0.3142 | 0.3172 | 0.3167 | 0.3653 | 0.3680 | 0.3653 | 0.3677 | 0.4568 | 0.3144 | 0.3160 | 0.9432 | 0.2981 | 0.2989 |
|  |  | 32 | 0.3018 | 0.3018 | 0.3020 | 0.3021 | 0.3337 | 0.3337 | 0.3318 | 0.3318 | 0.3314 | 0.3319 | 0.3300 | 0.3299 | 0.3080 | 0.3049 | 0.3080 | 0.3045 | 0.4291 | 0.2756 | 0.2776 | 0.9933 | 0.2752 | 0.2755 |
|  |  | 40 | 0.2551 | 0.2551 | 0.2548 | 0.2550 | 0.3125 | 0.3125 | 0.3122 | 0.3122 | 0.3120 | 0.3122 | 0.3132 | 0.3121 | 0.2601 | 0.2599 | 0.2601 | 0.2600 | 0.4474 | 0.2250 | 0.2239 | 1.0537 | 0.2236 | 0.2230 |
|  |  | 48 | 0.2176 | 0.2176 | 0.2177 | 0.2200 | 0.3134 | 0.3134 | 0.3133 | 0.3133 | 0.3133 | 0.3143 | 0.3137 | 0.3132 | 0.2248 | 0.2165 | 0.2248 | 0.2167 | 0.4191 | 0.1990 | 0.1991 | 0.9458 | 0.1914 | 0.1915 |
|  |  | 56 | 0.2324 | 0.2324 | 0.2325 | 0.2326 | 0.3185 | 0.3185 | 0.3189 | 0.3189 | 0.3189 | 0.3188 | 0.3190 | 0.3191 | 0.2378 | 0.2310 | 0.2378 | 0.2312 | 0.4514 | 0.2121 | 0.2129 | 1.0273 | 0.2073 | 0.2081 |
|  |  | 72 | 0.1978 | 0.1978 | 0.1978 | 0.1962 | 0.3095 | 0.3095 | 0.3096 | 0.3096 | 0.3096 | 0.3097 | 0.3090 | 0.3098 | 0.2001 | 0.1976 | 0.2001 | 0.1975 | 0.4137 | 0.1761 | 0.1757 | 1.0002 | 0.1796 | 0.1794 |
|  |  | 88 | 0.1919 | 0.1919 | 0.1920 | 0.1917 | 0.3101 | 0.3101 | 0.3097 | 0.3097 | 0.3097 | 0.3095 | 0.3090 | 0.3104 | 0.1956 | 0.1919 | 0.1956 | 0.1917 | 0.4212 | 0.1749 | 0.1747 | 1.0388 | 0.1713 | 0.1714 |
|  |  | 120 | 0.1594 | 0.1594 | 0.1594 | 0.1591 | 0.3071 | 0.3071 | 0.3073 | 0.3073 | 0.3073 | 0.3073 | 0.3073 | 0.3073 | 0.1579 | 0.1604 | 0.1579 | 0.1604 | 0.4313 | 0.1432 | 0.1431 | 1.0325 | 0.1417 | 0.1418 |
|  |  | 184 | 0.1141 | 0.1141 | 0.1142 | 0.1138 | 0.2990 | 0.2990 | 0.2991 | 0.2991 | 0.2991 | 0.2992 | 0.2991 | 0.2991 | 0.1146 | 0.1145 | 0.1146 | 0.1145 | 0.4270 | 0.1023 | 0.1023 | 1.0163 | 0.1014 | 0.1013 |
|  |  | 312 | 0.0853 | 0.0853 | 0.0853 | 0.0851 | 0.2948 | 0.2948 | 0.2950 | 0.2950 | 0.2950 | 0.2950 | 0.2955 | 0.2949 | 0.0867 | 0.0859 | 0.0867 | 0.0859 | 0.3878 | 0.0799 | 0.0800 | 0.9974 | 0.0797 | 0.0797 |
